# Inhibitors of malaria parasite cyclic nucleotide phosphodiesterases block asexual blood-stage development and mosquito transmission

**DOI:** 10.1126/sciadv.adq1383

**Published:** 2024-12-06

**Authors:** Paula-Josefina Gomez-Gonzalez, Antima Gupta, Laura G. Drought, Avnish Patel, John Okombo, Mariëtte van der Watt, Ryan Walker-Gray, Kyra A. Schindler, Anna Y. Burkhard, Tomas Yeo, Sunil K. Narwal, Talia S. Bloxham, Christian Flueck, Eloise M. Walker, Joshua A. Rey, Kate J. Fairhurst, Janette Reader, Heekuk Park, Harry G. Pollard, Lindsay B. Stewart, Luke Brandner-Garrod, Mojca Kristan, Geert-Jan Sterk, Youri M. van Nuland, Emilia Manko, Donelly A. van Schalkwyk, Yang Zheng, Rob Leurs, Koen J. Dechering, Anna Caroline C. Aguiar, Rafael V. C. Guido, Dhelio B. Pereira, Patrick K. Tumwebaze, Samuel L. Nosbya, Philip J. Rosenthal, Roland A. Cooper, Mike Palmer, Tanya Parkinson, Jeremy N. Burrows, Anne-Catrin Uhlemann, Lyn-Marié Birkholtz, Jennifer L. Small-Saunders, James Duffy, David A. Fidock, Alan Brown, Mark Gardner, David A. Baker

**Affiliations:** ^1^ Faculty of Infectious and Tropical Diseases, London School of Hygiene & Tropical Medicine, London, UK.; ^2^ Department of Microbiology and Immunology, Columbia University Irving Medical Center, New York, NY, USA.; ^3^ Center for Malaria Therapeutics and Antimicrobial Resistance, Columbia University Irving Medical Center, New York, NY, USA.; ^4^ Department of Biochemistry, Genetics and Microbiology, University of Pretoria, Pretoria, South Africa.; ^5^ Division of Infectious Diseases, Department of Medicine, Columbia University Irving Medical Center, New York, NY, USA.; ^6^ Institute for Sustainable Malaria Control, University of Pretoria, Pretoria, South Africa.; ^7^ Vrije Universiteit Amsterdam, Amsterdam, Netherlands.; ^8^ TropIQ Health Sciences, Nijmegen, Netherlands.; ^9^ Federal University of São Paulo, São Paulo, Brazil.; ^10^ Sao Carlos Institute of Physics, University of São Paulo, São Carlos, Brazil.; ^11^ Research Center for Tropical Medicine of Rondonia, Porto Velho, Brazil.; ^12^ Infectious Diseases Research Collaboration, Kampala, Uganda.; ^13^ University of California, San Francisco, CA, USA.; ^14^ Dominican University of California, San Rafael, CA, USA.; ^15^ Salvensis, Sandwich, UK.; ^16^ Medicines for Malaria Venture, Geneva, Switzerland.; ^17^ Liverpool School of Tropical Medicine, Liverpool, UK.

## Abstract

Cyclic nucleotide–dependent phosphodiesterases (PDEs) play essential roles in regulating the malaria parasite life cycle, suggesting that they may be promising antimalarial drug targets. PDE inhibitors are used safely to treat a range of noninfectious human disorders. Here, we report three subseries of fast-acting and potent *Plasmodium falciparum* PDEβ inhibitors that block asexual blood-stage parasite development and that are also active against human clinical isolates. Two of the inhibitor subseries also have potent transmission-blocking activity by targeting PDEs expressed during sexual parasite development. In vitro drug selection experiments generated parasites with moderately reduced susceptibility to the inhibitors. Whole-genome sequencing of these parasites detected no mutations in PDEβ but rather mutations in downstream effectors: either the catalytic or regulatory subunits of cyclic adenosine monophosphate–dependent protein kinase (PKA) or in the 3-phosphoinositide-dependent protein kinase that is required for PKA activation. Several properties of these *P. falciparum* PDE inhibitor series make them attractive for further progression through the antimalarial drug discovery pipeline.

## Teaser

Viagra-like compounds have the potential to cure patients with malaria and block transmission to mosquitoes.

## INTRODUCTION

There were an estimated 249 million clinical cases of malaria worldwide resulting in 608,000 deaths in 2022 (*1*). Between 2000 and 2013, sharp decreases in malaria morbidity and mortality were achieved through increased use of effective mosquito control strategies and the improved diagnosis and treatment of patients with artemisinin-based combination treatments. However, progress plateaued after 2013, partly due to increases in mosquito insecticide resistance and malaria parasite drug resistance. This was followed by increased malaria cases and deaths since 2020, associated with challenges imposed by the COVID-19 pandemic (*1*). Thus, additional antimalarial drugs are needed urgently as components of new combination treatments for malaria control and elimination. Several promising compound series have been identified either by high-throughput screening (*2*–*4*) or via chemistry programs that focus on inhibitors of individual targets within essential biochemical pathways (*5*, *6*). However, the drug discovery pipeline needs to remain active because of the high attrition rate of compounds reaching the clinic.

The life cycle of the most lethal malaria parasite, *Plasmodium falciparum*, is complex, with phases in both the human host and mosquito vector. Malaria pathology is caused exclusively by the asexual blood-stage (ABS) parasites, which undergo multiple ~48-hour cycles of replication within erythrocytes. A subpopulation of ABS parasites differentiates into sexual precursors called gametocytes. Transmission occurs when an *Anopheles* mosquito ingests mature male and female gametocytes from an infected person during a blood meal. About 2 weeks of parasite development then occurs in the mosquito following gamete fertilization, and the cycle is continued when the infected mosquito bites a new human host, injecting sporozoites that invade liver cells. Parasites then multiply there for around 7 days before initiation of ABS infection.

We and others have previously shown that progression of all key stages of the malaria parasite life cycle is controlled by cyclic nucleotide signaling, mediated by the intracellular messenger molecules cyclic adenosine monophosphate (cAMP) and cyclic guanosine monophosphate (cGMP) (*7*, *8*). Cellular levels of cyclic nucleotides are balanced by the opposing action of two classes of enzymes: synthesis by cyclases and hydrolysis by phosphodiesterases (PDEs). On reaching threshold levels, cAMP and cGMP activate their respective cyclic nucleotide–dependent protein kinases, PKA and PKG, that phosphorylate numerous target proteins to mediate changes in cell function. We showed that cGMP signaling is essential for blood-stage merozoite egress from erythrocytes (*9*–*12*), whereas cAMP signaling is essential for erythrocyte invasion and early ring-stage development (*13*–*16*). We reasoned that selectively targeting the enzymatic components of the cyclic nucleotide signaling pathway with drugs might be a viable strategy to prevent ABS replication and thereby cure disease with the additional possibility of targeting sexual-stage parasites to block transmission. In support of this approach, we previously reported in vivo proof of concept for inhibiting the *P. falciparum* PKG by developing a highly potent inhibitor that blocks blood-stage egress, reduces infection to undetectable levels in a *P. falciparum* severe combined immunodeficient rodent model engrafted with human red blood cells (RBCs), and blocks transmission to mosquitoes (*5*). In the present study, we focused on targeting the malaria parasite PDEs with small-molecule inhibitors, given the strong precedent for their safe and effective use in treating other human conditions. For example, the human PDE5 inhibitors, sildenafil (Viagra) and tadalafil are approved to treat disorders such as erectile dysfunction and pulmonary arterial hypertension (*17*).

The *P. falciparum* genome encodes four PDEs that are each expressed at defined stages of the life cycle (*7*). These four isoforms all have six predicted transmembrane domains, and previous work has confirmed that the enzymes are membrane associated (*18*–*21*). PDEα is expressed in ABS parasites and mature gametocytes but can be deleted without apparent *P. falciparum* ABS (*20*) or *Plasmodium berghei* gametocyte phenotypes (*22*). In *Plasmodium yoelii*, PDEγ has been shown to play an essential role in the migration of sporozoites to the mosquito salivary glands (*23*). PDEδ in *P. falciparum* and *P. berghei* is essential for sexual development and transmission to the mosquito (*19*, *22*, *24*). Although these three enzymes have been previously reported to hydrolyze only cGMP (*19*, *20*, *22*, *23*), there is a recent report of dual activity for PDEδ in *P. falciparum* (*25*). We recently demonstrated using conditional knockout that the fourth enzyme, PDEβ, is the only isoform that is essential for *P. falciparum* ABS development. Furthermore, pull-down assays using an epitope-tagged form of PDEβ demonstrated that it is a dual-specific PDE that can hydrolyze both cAMP and cGMP (*18*).

Here, we report the antimalarial properties of newly synthesized derivatives of three chemical series based on compounds originally designed by Pfizer as part of drug discovery programs targeting human PDEs. These compounds have potent activity against *P. falciparum* ABS development with a fast/moderate killing rate. The compounds are also active against a multidrug-resistant laboratory line [Dd2 (*26*, *27*)] and clinical isolates from Africa and South America. Analogs of two of these series also have potent activity in cell-based transmission-blocking assays, where we measured the effects on gametocyte viability, gamete formation, and mosquito infection. Some of the new compounds showed >100-fold selectivity against human PDE5 and/or PDE6, which are the most relevant to our human PDE5 inhibitor–derived structures. In vitro drug selection experiments combined with whole-genome sequencing revealed that mutations in one of three downstream effectors of cAMP signaling reduced susceptibility to inhibitors in ABS parasites, adding further strong evidence that their target in ABS parasites is PDEβ. In summary, these new *P. falciparum* PDE inhibitors are attractive candidates for further development with the potential for inclusion in future antimalarial combination treatments or chemoprevention.

## RESULTS

### Three chemical series have potent activity against early ABS development and against cAMP hydrolysis by PDEβ

**Fig. 1.**
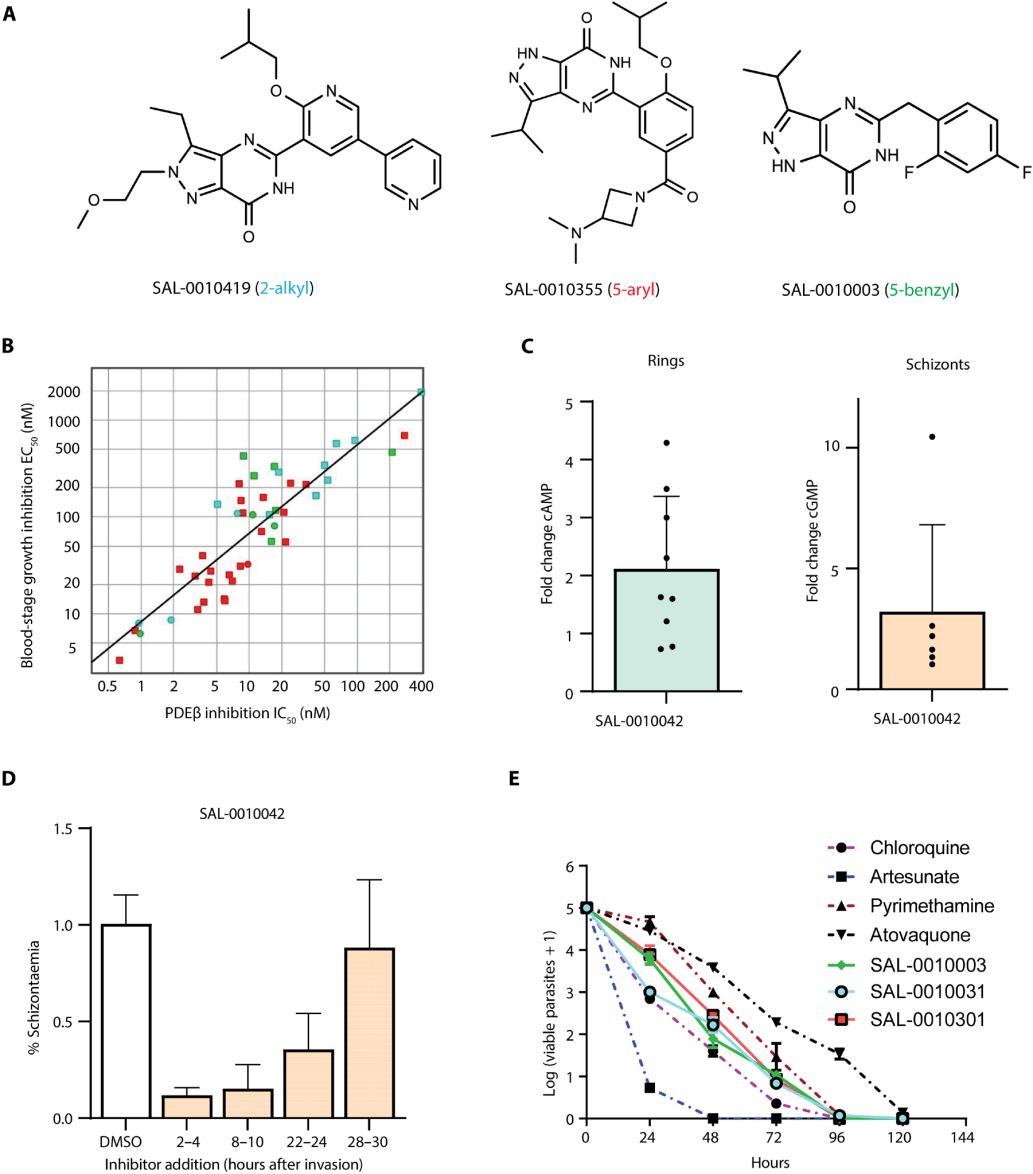
Structures and activity of the PDE inhibitor series against ABS parasites in biochemical and cell-based assays. (**A**) Structure of an example of the 2-alkyl, 5-aryl, and 5-benzyl PDE inhibitor subseries. (**B**) Plot showing the correlation between IC_50_ values for inhibition of cAMP hydrolysis by PDEβ in *P. falciparum* schizont lysates and EC_50_ values for inhibition of *P. falciparum* ABS growth for all three subseries. Green, 5-benzyl; red, 5-aryl; blue, 2-alkyl. Circles correspond to the main compounds in this study, and squares indicate compounds tested but not further described herein. Pearson’s correlation coefficient, *r* = 0.841. (**C**) Bar charts showing the fold change in elevated cAMP levels in rings (*n* = 9) and elevated cGMP levels in schizonts (*n* = 6) [measured by enzyme-linked immunosorbent assay (ELISA)] following treatment with a 5-benzyl compound (SAL-0010042) compared to dimethyl sulfoxide (DMSO) treatment. Error bars show the means ± SD with individual replicate measurements shown as black dots. (**D**) Bar chart showing the effects of a 5-benzyl compound (SAL-0010042) on schizont formation (assessed by measuring DNA content by flow cytometry) when added to ring-stage parasites at four different time windows after erythrocyte invasion. (**E**) Plot showing the *P. falciparum* ABS killing profiles for representatives of the three subseries generated using parasite reduction ratio (PRR) assays. The reduction in the numbers of viable parasites measured is shown for each test compound (colored lines), over time compared to control antimalarials (colored hatched lines). All compounds were used at a concentration of 10× EC_50_.

We identified three PDE inhibitor chemical series generated by Pfizer, with structures in the public domain, which have activity against malaria parasites (see the “Chemistry strategy summary” section). These series were originally designed to inhibit human PDEs and were chosen as chemistry start points to optimize activity against malaria parasites. More than 400 new analogs were generated (full details of their properties will be reported in a separate paper, in preparation) through iterative rounds of synthesis and testing. The chemistry start points for each of the series were SAL-0010042 (5-benzyl), SAL-0010043 (5-aryl), and SAL-0010034 (2-alkyl) (see Materials and Methods and fig. S1A). Chemical structures of the 15 compounds on which this study is focused are shown in fig. S2. Analytical data for these compounds are provided in data S1. All newly synthesized compounds were tested for inhibition of native cAMP-PDE activity in mature schizont crude membrane preparations and inhibition of *P. falciparum* ABS growth. Examples of curves used to generate median inhibitory concentration (IC_50_) data for each subseries are shown in fig. S3A. We previously showed, using conditional gene knockout, that PDEβ is the only enzyme that can hydrolyze cAMP in *P. falciparum* ABS parasites; therefore, measurement of inhibition of cAMP hydrolysis at this life cycle stage is a measurement of inhibition of PDEβ activity in the schizont extract. To confirm this, we tested the effects of the compounds for inhibition of immunoprecipitated hemagglutinin (HA)–tagged PDEβ and found them to be very similar to results of assays using parasite extracts (examples shown in fig. S3B). All three series (example structures shown in Fig. 1A) showed potent activity against cAMP hydrolysis in schizont extracts, with IC_50_ values down to single-digit nanomolar concentrations (Table 1). The 5-aryl series had the most potent activity against ABS parasites [median effective concentration (EC_50_) values down to 21 nM]. The best EC_50_ value for the 2-alkyl series was 67 nM, whereas the 5-benzyl series had somewhat lower potency against ABS parasites, with a best EC_50_ value of 142 nM. Figure 1B shows the strong correlation between ABS growth inhibition (expressed as a free concentration, i.e., corrected for protein binding in the assay to which AlbuMAX is added) and inhibition of cAMP hydrolysis by PDEβ across all three chemical series, consistent with the mode of action of the compounds being through inhibition of PDEβ activity. The unbound EC_50_ values for the compounds in the parasite growth inhibition assay were consistently calculated to be sevenfold higher than the IC_50_ values in the biochemical assay. Given that PDEβ is dual specific (i.e., can hydrolyze both cAMP and cGMP), we also determined IC_50_ values for cGMP hydrolysis. However, PDEα also hydrolyzes cGMP; therefore, IC_50_ values encompass inhibition of both enzymes. The IC_50_ values measured for cGMP hydrolysis in blood-stage schizonts were generally similar to those for cAMP hydrolysis (Table 1). The simplest interpretation is that the compounds have additional activity against PDEα. Our previous alignment of the catalytic domains of the four *P. falciparum* PDEs (PfPDEs) (*18*) shows the high levels of identity in binding pocket residues.

**Table 1.** Key activity data for the three main chemical subseries against ABS parasites, parasite fractions, and HepG2 cells. ND, not determined; ×/÷ is the geometric mean with geometric SD.

Compound name	Subseries	3D7 EC_50_ blood stage (nM)	Dd2 EC_50_ blood stage (nM)	Schizont IC_50_ cAMP hydrolysis (nM)	Schizont IC_50_ cGMP hydrolysis (nM)	Gametocyte IC_50_ cGMP hydrolysis (nM)	HepG2 EC_50_ (nM) (selectivity index)
SAL-0010042	5-benzyl	142 ×/÷ 1.0	218 ×/÷ 1.6	10.8 ×/÷ 4.3	13.1 ×/÷ 1.4	48.9 ×/÷ 2.3	ND
SAL-0010003	5-benzyl	327 ×/÷ 1.3	ND	17.3 ×/÷ 1.6	10.6 ×/÷ 4.9	85.1 ×/÷ 2.1	>25,000 (77)
SAL-0010007	5-benzyl	191 ×/÷ 1.3	ND	20.6 ×/÷ 1.2	29.5 ×/÷ 1.2	333 ×/÷ 1.4	ND
SAL-0010028	2-alkyl	144 ×/÷ 2.3	ND	1.9 ×/÷ 5.8	164 ×/÷ 172	>3,680 ×/÷ 7.4	ND
SAL-0010039	2-alkyl	131 ×/÷ 1.1	ND	2.9 ×/÷ 1.4	< 0.7 ×/÷ 1.7	>10,000 ×/÷ 1.0	ND
SAL-0010255	2-alkyl	114 ×/÷ 1.2	126 (*n* = 1)	8.3 ×/÷ 1.9	< 8.9 ×/÷ 12.0	>5,880 ± 2.5	ND
SAL-0010031	2-alkyl	175 ×/÷ 1.1	ND	7.8 ×/÷ 1.2	< 14.3 ×/÷ 72.2	>10,000 ×/÷ 1.0	>25,000 (143)
SAL-0010419	2-alkyl	66.5 ×/÷ 1.2	ND	15.9 ×/÷ 3.1	28.7 ×/÷ 4.8	>10,000 ± 1.0	ND
SAL-0010243	5-aryl	39.6 ×/÷ 1.0	ND	8.1 ×/÷ 3.1	16.8 ×/÷ 2.4	444 ×/÷ 4.5	ND
SAL-0010203	5-aryl	260 ×/÷ 1.5	ND	2.4 ×/÷ 9.3	< 1.9 ×/÷ 9.8	>2,350 ×/÷ 3.6	ND
SAL-0010283	5-aryl	58.6 ×/÷ 1.8	ND	21.6 ×/÷ 2.9	41.4 ×/÷ 1.2	>3,009 ×/÷ 10.5	ND
SAL-0010284	5-aryl	58.8 ×/÷ 1.1	28.9 ×/÷ 1.0	36.1 ×/÷ 5.5	85.8 ×/÷ 11.4	>2,520 ×/÷ 10.9	ND
SAL-0010301	5-aryl	90.2 ×/÷ 1.3	ND	9.8 ×/÷ 1.7	< 4.6 ×/÷ 7.0	92.5 ×/÷ 1.5	>25,000 (277)
SAL-0010355	5-aryl	21.1 ×/÷ 1.0	41.4 ×/÷ 1.2	11.5 ×/÷ 2.1	5.2 ×/÷ 3.6	1,200 ×/÷ 5.3	>25,000 (1,185)
SAL-0010333	5-aryl	34.4 ×/÷ 1.1	46.9 ×/÷ 1.3	8.89 ×/÷ 1.9	77.4 ×/÷ 43.0	77.2 ± 1.8	>25,000 (727)

Further confirmation of the mechanism of action was obtained by showing that addition of a PDE inhibitor to *P. falciparum* ABS parasites leads to elevated cAMP and cGMP levels that were most reproducibly detected in rings and schizonts, respectively (Fig. 1C). This is consistent with the known role of cAMP in RBC invasion/early ring-stage development and cGMP in egress and likely reflects the distinct peaks of cAMP and cGMP hydrolysis at those stages.

We previously showed, using conditional gene knockout in *P. falciparum*, that the absence of PDEβ blocks ABS development. The PDEβ null phenotype comprises a ~70% reduction in RBC invasion. The remaining ~30% of merozoites can invade but then degenerate and do not progress beyond early ring stage (*18*).

To examine the effects of the PDEβ inhibitors on ring-stage development, we added the 5-benzyl chemistry start point, SAL-0010042 (1 μM), to highly synchronized *P. falciparum* cultures at four different time points after invasion. We then measured the numbers of schizonts that formed the next day. This experiment showed that PDEβ inhibitors have the most pronounced effect on the development of ring stages into schizonts when added up to 10 hours after invasion, but they still have a marked effect when added 22 to 24 hours after invasion (Fig. 1D). These results are consistent with the phenotype of the PDEβ conditional knockout and suggest that ABS parasites are susceptible to PDE inhibitors for approximately half of the ABS cycle.

### PDE inhibitors display a fast/moderate speed of kill against *P. falciparum* ABSs

Artemisinin derivatives kill ABS parasites very rapidly, leading to quick recovery times in patients and reduced chances of recrudescence. This is a desirable characteristic for new antimalarial drugs; thus, the speed of kill is usually tested early in a drug discovery project. Examples of each subseries, SAL-0010301 (2-aryl), SAL-0010031 (2-alkyl), and SAL-0010003 (5-benzyl), were therefore tested in the gold standard parasite reduction ratio (PRR) assays to obtain killing profiles for the three distinct chemical series. The test compounds displayed a fast/moderate killing profile that is faster than pyrimethamine and slower than chloroquine (Fig. 1E and fig. S4). Most of the parasite population was cleared after 96 hours of treatment. Unlike pyrimethamine and atovaquone, the PDE inhibitors did not have a delayed effect (lag phase). Table 2 shows the log PRR and PCT_99.9%_ (the number of hours taken to kill 99.9% of the parasites) values for the test compounds.

**Table 2.** PPR data derived from killing profile experiments carried out with an example of each of the three subseries and four antimalarial controls. The lag phase is the time needed to observe the maximal killing effect of the drug being tested, *R*^2^ is the coefficient of determination, and PCT_99.9%_ is the number of hours taken to kill 99.9% of the parasites. NA, not applicable.

Compound	Dose (μM)	Lag phase (hours)	Slope	*R* ^2^	Log PRR	PCT_99.9%_ (hours)
SAL-0010003	2.012	0	−0.0647 ± 0.0047	0.95	3.11	47.1
SAL-0010031	1.148	0	−0.0579 ± 0.0058	0.909	2.78	51.2
SAL-0010301	0.599	0	−0.0532 ± 0.0042	0.942	2.55	55.1
Controls						
Pyrimethamine	0.27	24	−0.0667 ± 0.0059	0.928	3.2	63.1
Artesunate	0.223	0	NA	NA	>8	<24
Chloroquine	0.224	0	−0.0717 ± 0.0037	0.974	3.44	39.0
Atovaquone	0.005	48	−0.0470 ± 0.0042	0.927	2.26	73.0

### Two of the chemical series also had potent activity against cGMP hydrolysis in mature *P. falciparum* gametocytes and in transmission blocking assays

**Fig. 2.**
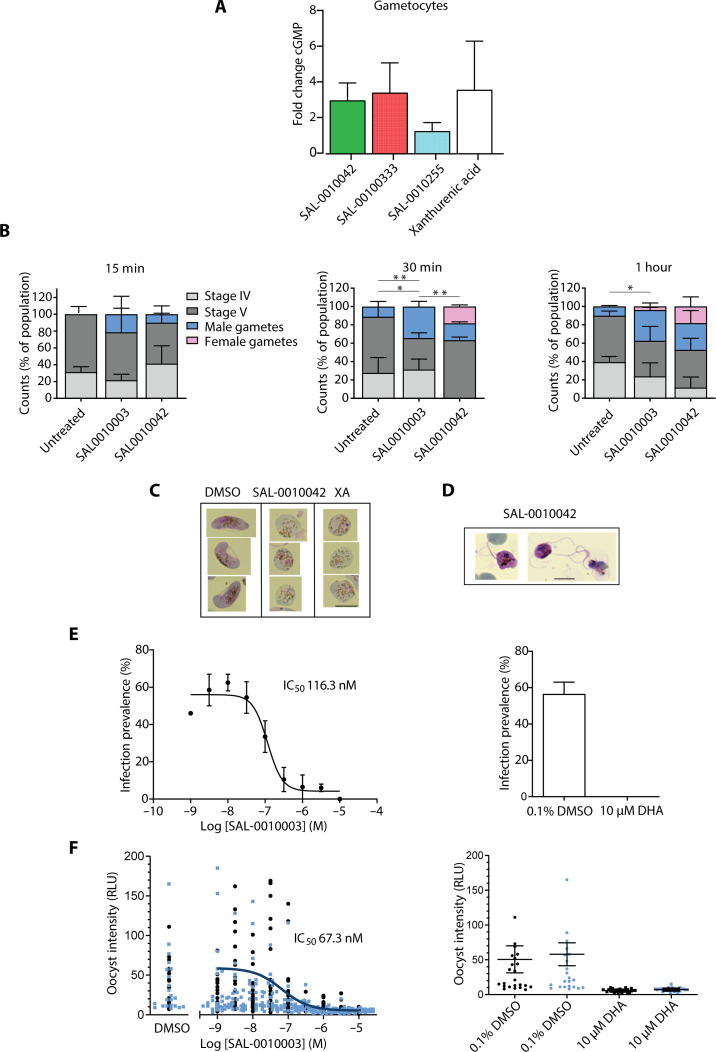
Activity of the inhibitors against sexual-stage parasites. (**A**) Bar chart showing the fold change in elevated cGMP levels (measured by ELISA) in stage IV to V gametocytes following treatment with representatives of the three subseries or with XA compared to DMSO treatment. Experiments were carried out three times in duplicate. Error bars show the means ± SD. Compounds subseries are indicated by the following colors: green, 5-benzyl; red, 5-aryl; blue, 2-alkyl. (**B**) Bar charts showing the ability of representatives of each subseries to stimulate gametogenesis following three different treatment durations. The proportions of the treated cultures that were stage IV gametocytes, stage V gametocytes, and male and female gametes, indicated by the color of the bars, were identified by examination of parasite morphology in Giemsa-stained blood films. *P < 0.05 and **P < 0.01 using an unpaired Student’s *t* test. (**C**) Images of Giemsa-stained blood films containing stage V gametocytes or “rounded-up” gametocytes/gametes following treatment with DMSO, the 5-benzyl compound SAL-0010042, or XA. Scale bar, 7 μm. (**D**) Images of Giemsa-stained blood films containing exflagellating male gametocytes/gametes following treatment with the 5-benzyl compound SAL-0010042. Scale bar, 7 μm. (**E** and **F**) Plots showing infection prevalence and intensity, respectively, for test and control compounds. Experiments were carried out with stage V gametocytes of the *P. falciparum* NF54 HGL reporter strain following incubation with the 5-benzyl compound SAL-0010003 for 48 hours before feeding to *A. stephensi* mosquitoes. Eight days after feeding, infection status was assessed by luminescence analysis of individual mosquitoes. All conditions were tested in duplicate feeders (respective intensities distinguished by blue or black dots) with 24 mosquitoes analyzed per feeder. Plots showing the results of SMFA data for SAL-0010003 compared to dihydroartemisinin (DHA) and DMSO controls. IC_50_ values for both the prevalence and intensity of infection are indicated. RLU, relative light units.

cGMP signaling is known to play an essential role in gametogenesis in *Plasmodium* (*19*, *20*, *28*). We therefore tested the effects of all newly synthesized analogs for their ability to inhibit cGMP hydrolysis in crude gametocyte membrane preparations. The 5-benzyl series generally had the best potency in these assays with IC_50_ values as low as 48.9 nM. The 5-aryl series also had good levels of activity with IC_50_ values down to 77.2 nM, whereas the 2-alkyl series was markedly less active with IC_50_ values of >3.7 μM (Table 1). Examples of dose-response curves for cGMP-PDE activity in gametocyte extracts are shown in fig. S5A. These findings were also consistent with our enzyme-linked immunosorbent assay (ELISA)–based measurement of elevated cGMP levels in gametocytes following incubation with an example of each subseries (Fig. 2A). The elevated levels of cGMP measured after adding either a 5-benzyl or 5-aryl compound (10× IC_50_ for inhibition of PDE activity in gametocyte lysates; Table 1) were similar to those obtained by adding 100 μM xanthurenic acid (XA) (a tryptophan derivative that stimulates gametogenesis in the mosquito), whereas the elevated cGMP levels were lower on addition of a 2-alkly compound (Fig. 2A). The levels of cAMP detected in gametocytes were similar in dimethyl sulfoxide (DMSO)–, XA-, and inhibitor-treated samples, and we could detect little or no cAMP hydrolysis in mature gametocytes (fig. S5B).

Both PDEα and PDEδ are expressed in gametocytes, can both hydrolyze cGMP, and are, therefore, potential targets for our compounds (fig. S5C). Selectivity for cGMP has previously been shown for PDEα, but there are conflicting data regarding whether PDEδ is selective for cGMP or is dual specific. To investigate this further, we immunoprecipitated HA-tagged PDEδ from a transgenic line and showed that it could only hydrolyze cGMP (fig. S6A). Immunoprecipitation of HA-tagged PDEβ from a transgenic line assayed in parallel was able to hydrolyze both cAMP and cGMP as previously reported (fig. S6A).

Because gametogenesis is stimulated by elevated cGMP levels and activation of PKG, we next tested whether two of the 5-benzyl series could stimulate this process. The short-term effects of SAL-0010042 and SAL-0010003 on gamete induction were measured over time (15, 30, and 60 min). Both compounds stimulated mature stage V gametocytes (>90%) to undergo gametogenesis within 15 min (Fig. 2B), with the effect pronounced for male gamete formation (*P* < 0.01) for both compounds. This induction was stimulated without addition of XA or a reduction in temperature (factors that are normally required to trigger gametogenesis in the mosquito midgut). The effects of SAL-0010042 on mature gametocytes were comparable to triggering male gamete exflagellation with XA (Fig. 2, C and D), and its effects were reversed in the presence of a PKG inhibitor, compound 2 (fig. S6B). Together, these findings are consistent with the hypothesis that the compounds trigger elevated cGMP levels and PKG activation, which, in turn, leads to gamete formation.

We then tested examples of each subseries for their ability to inhibit male and female gamete formation following prolonged exposure to inhibitors. Consistent with the trends observed with inhibition of cGMP-PDE activity in gametocyte lysates, the 5-benzyl and 5-aryl series generally showed the highest levels of inhibition of male and female gamete formation (90 to 100% inhibition when tested at a single concentration of 2 μM) with relatively low activity observed with the 2-alkyl series (6 to 66% at 2 μM). The IC_50_ values for two 5-benzyl compounds were determined. With SAL-0010003, the IC_50_ values for inhibition of male and female gamete formation were 404 and 273 nM, respectively. For SAL-0010042, these values were 386 and 238 nM (fig. S7).

To determine whether the compounds have any effect on gametocyte development before gamete formation, we tested examples of each of the three inhibitor subseries in a *P. falciparum* lactate dehydrogenase (LDH)–based stage IV to V gametocyte viability assay at 15 μM. High levels of inhibition of gametocyte viability were obtained with the 5-benzyl and 5-aryl series, but markedly less inhibition was measured with the 2-alkyl series (Table 3), which is in agreement with the results of the inhibition of PDE activity in gametocyte lysates. These data indicate that the PDE inhibitors have inhibitory activity not only on gamete formation in the mosquito but also on gametocytes themselves in the human host.

**Table 3.** Key activity data for the three main chemical subseries against human PDEs and sexual-stage parasites. Compounds tested in other key assays are indicated.

Compound name	IC_50_ human PDE5A activity (nM)	IC_50_ human PDE6C activity (nM)	% inhibition gametocyte viability at 15 μM	% inhibition of exflagellation at 2 μM	% inhibition of female gamete formation at 2 μM	Parasites selected under drug pressure	Clinical isolates tested	PRR value generated
SAL-0010042	632 ×/÷ 1.7	73 ×/÷ 1.4	99.6 ± 1.3	95.4 ± 18.0	97.7 ± 0.4		Yes	
SAL-0010003	793 ×/÷ 1.6	60.6 ×/÷ 1.4	65.4 ± 20.3	88.4 ± 17	71.3 ± 1.5	Yes		Yes
SAL-0010007	2,270 ×/÷ 1.6	223 ×/÷ 2.5	100 ± 0	100 ± 0	50.6 ± 1.9			
SAL-0010028	1,470 ×/÷ 2.4	237 ×/÷ 4.2	57.1 ± 27.0	18.6 ± 15.8	6.9 ± 2.6			
SAL-0010039	121 ×/÷ 1.9	981 ×/÷ 3.6	0	0	20.7 ± 2.3			
SAL-0010255	374 ×/÷ 1.6	93.6 ×/÷ 1.2	40.4 ± 3.1	48.8 ± 25.3	66.7 ± 1.8		Yes	
SAL-0010031	92.8 ×/÷ 1.2	448 ×/÷ 1.3	ND	ND	ND			Yes
SAL-0010419	326 ×/÷ 2.6	74.6 ×/÷ 1.1	ND	ND	ND	Yes		
SAL-0010243	37.2 ×/÷ 1.3	34.7 ×/÷ 2.5	100 ± 0	95.4 ± 12.3	80.5 ± 0.9			
SAL-0010203	42.5 ×/÷ 1.7	3.28 ×/÷ 2.2	0	76.7 ± 13.6	44.8 ± 1.7			
SAL-0010283	7.5 ×/÷ 1.0	< 45.9 ×/÷ 4.6	100 ± 0	72.1 ± 26.0	44.8 ± 2.5			
SAL-0010284	59.6 ×/÷ 8.6	< 47.0 ×/÷ 4.5	100 ± 0	60.5 ± 18.0	58.6 ± 2.1			
SAL-0010301	<6.75 ×/÷ 1.5	<7.78 ×/÷ 1.8	ND	ND	ND			Yes
SAL-0010355	10.1 ×/÷ 1.2	0.777 ×/÷ 1.8	ND	ND	ND	Yes		
SAL-0010333	<1.76 ×/÷ 5.8	< 0.016 ×/÷ 5.1	ND	ND	ND		Yes	

Last, we tested two of the 5-benzyl compounds (SAL-0010003 and SAL-0010042) in a standard membrane-feeding assay (SMFA), which is the gold standard for evaluation of transmission blocking activity of drugs and antibodies (*29*). Both compounds were initially tested at three concentrations, with a proportion of the mosquitoes examined for oocyst formation and a proportion examined 4 days later for sporozoite formation. In the absence of inhibitor (DMSO vehicle), the infection prevalence (the proportion of mosquitoes infected) and infection intensity were relatively high. Few or no oocysts or sporozoites were seen at the two higher concentrations (0.5 and 1 μM). At the lowest concentration (100 nM), the infection prevalence and intensity were reduced with SAL-0010003 (fig. S8).

A full dose response assay was then carried out for oocyst formation, and the IC_50_ value for SAL-0010003 was 116 nM for transmission prevalence (Fig. 2E) and 67 nM for transmission intensity (Fig. 2F). Parallel control experiments were carried out in which mosquitoes were fed either with 0.1% DMSO (negative control) or 10 μM dihydroartemisinin (DHA) (positive control), yielding high and low levels of transmission prevalence and intensity, respectively (Fig. 2, E and F). Overall, the results demonstrate the transmission blocking potential of PDE inhibitors. The SMFA results were consistent with the IC_50_ of SAL-0010003 in the cGMP hydrolysis (PDE) inhibition assay using gametocyte extracts, providing further evidence that the compounds also specifically target PDE activity in sexual-stage parasites. Together, the results show that the activity of the compounds against gametocytes and gamete formation is mediated by inhibition of cGMP hydrolysis.

### The PDE inhibitors were active against ex vivo *P. falciparum* cultures from Uganda and Brazil and also against a multidrug-resistant laboratory isolate

**Fig. 3.**
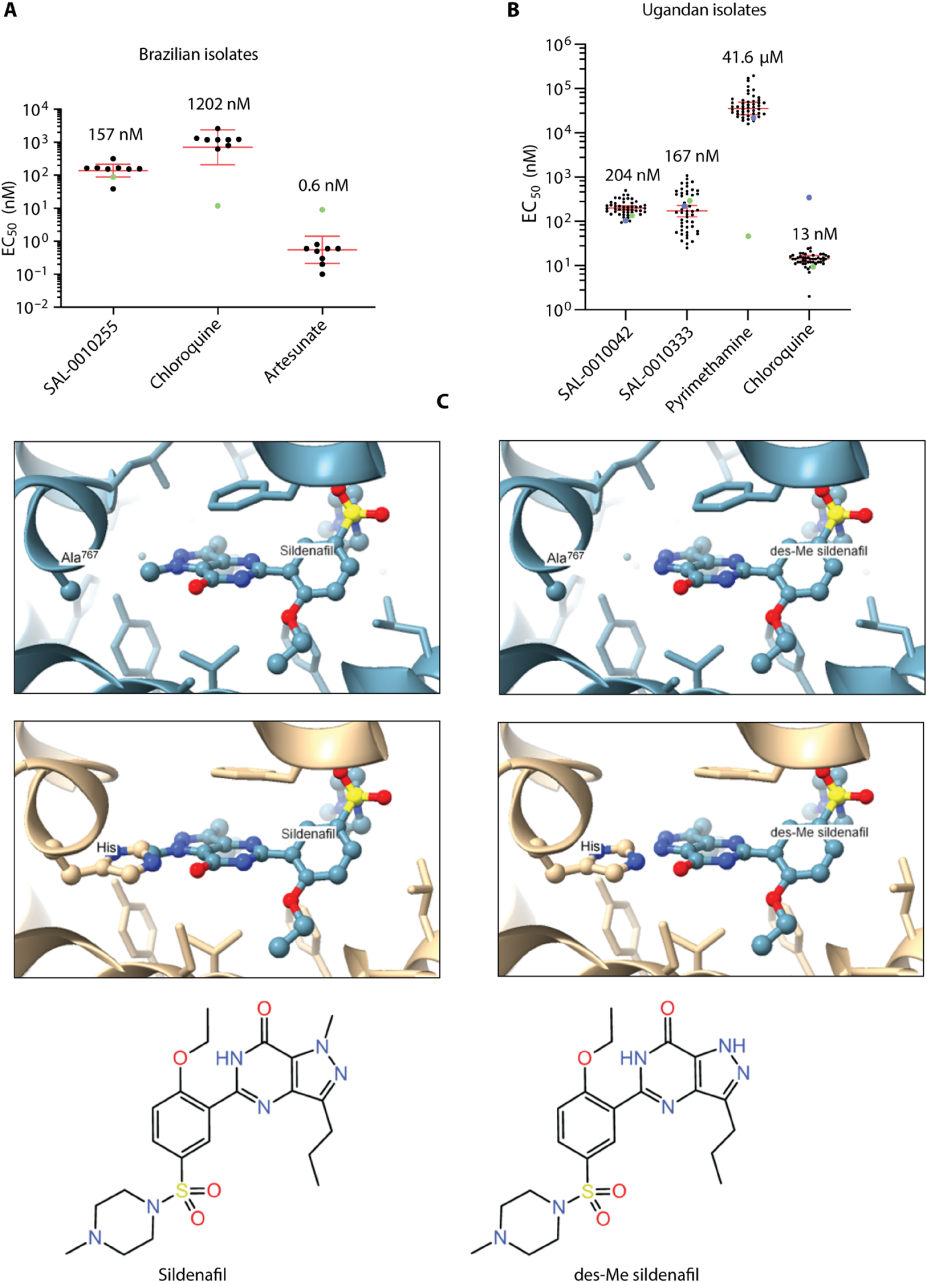
Activity of the inhibitor series against clinical isolates and selectivity against human PDE isoforms. (**A**) SAL-0010255 (2-alkyl) was tested for ex vivo activity against eight *P. falciparum* clinical isolates from Porto Velho, Rondônia in the Brazilian Amazon (black dots) and against the *P. falciparum* 3D7 control line (green dots). Chloroquine and artesunate were also tested in parallel. Red bars indicate the geometric mean EC_50_ values, and the error bars depict the 95% confidence interval for the clinical isolates. The values of the geometric means are shown for each compound. (**B**) SAL-0010042 (5-benzyl) and SAL-0010333 (5-aryl) and two control antimalarials were tested for activity against 47 clinical isolates from the Tororo and Busia Districts of Uganda. Black dots represent individual isolate EC_50_ values of the clinical isolates; enlarged blue dots indicate mean Dd2 control EC_50_ values; enlarged green dots indicate mean 3D7 control EC_50_ values. The red bars indicate the geometric mean, and the error bars depict the 95% confidence interval. The values of the geometric means are shown for each compound. (**C**) In light blue (top), a high-resolution crystal structure of the catalytic site of human PDE5 (*66*) (code 1TBF) is shown bound to sildenafil. des-Me sildenafil is visualized assuming the same coordinates as for sildenafil in 1TBF. The atoms of sildenafil and the side chain of Ala^767^ are shown with ball and stick. Other atoms are depicted as stick or cartoon. In beige (middle), the sequence of PDEβ has been modeled onto 1TBF using Phyre2 (*67*). The structures of sildenafil and des-Me sildenafil are shown in the bottom panels.

To explore their potential therapeutic relevance in malaria-endemic regions, we tested representative compounds from the three subseries against *P. falciparum* isolates either from eastern Uganda or from Porto Velho, Rondônia in the Brazilian Amazon. SAL-0010255 (2-alkyl) was tested for activity against eight Brazilian clinical isolates and had a median EC_50_ value of 157 nM with a range of 39 to 321 nM in the individual isolates. For the 3D7 clone, the EC_50_ value was 89 nM, which was comparable to the patient isolates (Fig. 3A, fig. S9, and table S1A). Artesunate and chloroquine were assayed in parallel and had median EC_50_ values of 0.6 and 1202 nM, respectively. These isolates all exhibited very high levels of chloroquine resistance because the IC_50_ with chloroquine-sensitive clone 3D7 was 12 nM (Fig. 3A and fig. S9).

SAL-0010042 (5-benzyl) and SAL-0010333 (5-aryl) and two control antimalarials were tested for activity against 47 Ugandan clinical isolates (Fig. 3B and table S1B). SAL-0010042 had a geometric mean EC_50_ value of 204 nM (range 94 to 499 nM), and SAL-0010333 had a geometric mean EC_50_ value of 167 nM (range 25 to 1072 nM). Although the mean values were comparable to data obtained with the 3D7 clone, with the latter compound, there was evidence of solubility or stability problems as indicated by the range and temporal clustering of high EC_50_ values that likely reflected decreased compound stability or solubility over time. All of the 47 clinical isolates evaluated were pyrimethamine resistant (table S1B), and none appeared chloroquine resistant.

Selected compounds were also tested for activity against the multidrug-resistant laboratory isolate Dd2 [originally derived from isolate W2 (*27*)], which is resistant to chloroquine, quinine, mefloquine, pyrimethamine, and sulfadoxine. All showed EC_50_ values similar to those obtained with 3D7 (Table 1), confirming that the underlying multidrug-resistant genotype of Dd2 does not confer cross-resistance to the PDE inhibitors and is consistent with the findings with the Ugandan isolates (table S1B).

### Two of the subseries have good selectivity against human PDE5 and PDE6

Since our chemical start points were originally designed to inhibit human PDE5, we tested all newly synthesized compounds against recombinant human PDE5 and the related human PDE6 to monitor the extent of selectivity (Table 3). The catalytic domain of PDEβ (Tyr^846^-Phe^1055^) has 27.6% identity (and 23.3% similarity) to human PDE5 and 26.7% identity (and 23.8% similarity) to human PDE6. Compounds in the 2-alkyl series had the best selectivity window when comparing activity against native PDEβ and recombinant human PDE5 or PDE6 (Table 3). For example, SAL-0010028 had the best selectivity against human PDE5 (773-fold), and SAL-0010039 had the best selectivity against human PDE6 (338-fold). Some compounds in the 5-benzyl series also had good levels of selectivity against human PDE5 (59- to 110-fold) but generally much poorer against PDE6 (3.5- to 10.8-fold). Most of the compounds in the 5-aryl series had poor selectivity against PDE5 and PDE6, and some were more potent against human PDE6 than against PDEβ; therefore, selectivity presents a major issue with this subseries (Table 3).

Despite many attempts by several groups, there are no high-resolution protein crystal structures of *Plasmodium* PDE enzymes. However, homology with PDEs from other species is at a level where structural models can be generated with high confidence. Models of malarial PDEs constructed from crystal structures of human PDE5, PDE6, and PDE9 all place the analogous active site residues in the same regions of the active site. While we believe that the homology models produced are not accurate enough for quantitative prediction of binding energy, qualitative assessments of structure-activity relationship (SAR) and hypotheses driving design can be made. A simple but startling example of this is illustrated in Fig. 3C. While many key residues in the binding pockets of human PDE5 and *P. falciparum* PDEβ are identical, an example of a difference is highlighted. In the human PDE5 crystal structure, the *N*-methyl group of sildenafil attached to the pyrazole ring points almost directly at the methyl group of Ala^767^ (Fig. 3C) [as numbered previously (*30*)]. In PDEβ, this residue is replaced with His. Not surprisingly, the much larger His^1011^ residue affects the binding of ligands to PDEβ. It is a remarkable observation that removing this *N*-methyl group from sildenafil changes the compound from essentially inactive against PDEβ (IC_50_ > 10 μM) to a compound with an IC_50_ of 36.7 nM and a difference of >250-fold. This translates to respective EC_50_ values against ABS parasites of >25 μM for sildenafil and 415 nM for desmethyl (des-Me) sildenafil and a difference of >60-fold. Together, these findings suggest that it should be feasible to deliver a selective *Plasmodium* PDE inhibitor.

### In vitro resistance selections yielded parasites with mutations in protein kinases that operate downstream of PDEβ to mediate cAMP signaling

To determine whether resistant parasites could be selected under drug pressure, we incubated a representative of each of the three inhibitor subseries with 2 × 10^5^ ABS parasites (Dd2-B2 clone) per well ×96 wells, at a concentration of 3× EC_90_ for 60 days. Cultures were checked for recrudescent parasites three times per week by flow cytometry. Parasites first appeared on days 13 to 17, and shifts in EC_50_ values of between 4.8- and 9.2-fold were measured (Table 4, fig. S10A, and table S2A).

**Table 4.** A summary of the amino acid substitutions selected in vitro with the six PDEβ inhibitors used in these studies. The mean fold shift in EC_50_ values (±SD) of the selected parasite lines compared to the Dd2-B2 parental line are also shown (see table S6).

Compound	Substitutions selected in vitro			Mean fold change in EC_50_
	PKAc	PKAr	PDK1	
SAL-0010355	P305S			6.1 ± 0.7
SAL-0010419	P305S			5.4 ± 0.6
SAL-0010003			N324I	7.6 ± 2.0
NPD-2958		F375I	P314T; N315Y	8.5 ± 1.4
NPD-3518	V304L	G376V	P314T; N315Y	12.2 ± 0.7
NPD-3738	A198S			11.3 ± 3.7

Three examples of an additional compound series developed independently [NPD-2958, NPD-3518 (*31*), and NPD-3738; fig. S1B] were also tested for in vitro resistance generation by incubating each compound with 2.5 × 10^6^ and 3 × 10^7^ ABS parasites at a concentration of 3× IC_90_ for 60 days. In these experiments, parasites first appeared on days 15 to 25, and shifts in EC_50_ values of between 6.9- and 14.7-fold were measured (Table 4, fig. S10B, and table S2B).

To identify the genetic loci underlying these changes in inhibitor susceptibility, we carried out whole-genome sequencing of samples using Illumina MiSeq. For both the 5-aryl (SAL-0010355) and 2-alkyl (SAL-0010419) compounds, a single-nucleotide change (Cca/Tca) in the gene encoding the catalytic subunit of the cAMP-dependent protein kinase (PKAc; PF3D7_0934800) was identified (table S3, A and B). This mutation introduced a proline-to-serine substitution at position 305; the N-terminal proline within a PxxP motif, conserved among protein kinases, and is critical for interlobe movement of the N- and C-lobes of the catalytic domain (fig. S11, top). For the 5-benzyl compound (SAL-0010003), a single-nucleotide change (aAt/aTt) in the gene encoding the 3-phosphoinositide–dependent protein kinase 1 (PDK1; PF3D7_1121900) was detected (table S3C). This introduced an asparagine-to-isoleucine substitution at position 324, which is immediately downstream of the activation loop. This loop contains a highly conserved threonine residue that is phosphorylated to allow activation of the kinase. In PDK1, this process is thought to be an autophosphorylation event, whereas in other mammalian AGC kinases, this threonine is phosphorylated by PDK1 itself. Figure S11 (middle) shows two conserved motifs within the activation loop, the activation loop threonine, and an unusual ~120–amino acid insert in this region that occurs in *Plasmodium* parasite PDK1 orthologs. It has recently been confirmed that PDK1 is required for activation of PKA in *P. falciparum*. In that study, five mutations in PDK1 were selected in transgenic clones that survived the lethal effects of PKAc overexpression (*14*).

Two mutations in both the PKAc and PDK1 loci were also selected in parasites with the NPD series (table S4 and fig. S1B). In PKAc, these were A198S (Gct/Tct) that is within the canonical APE motif at the N terminus of the activation loop and V304L (Gtt/Ctt) that is immediately adjacent to the P305S mutation (within the PxxP motif) selected with the 2-alkyl and 5-aryl series, respectively (fig. S11, top). In PDK1, the substitutions were P314T (Cct/Act) and N315Y (Aat/Tat), which are just upstream of the C-terminal boundary of the activation loop (fig. S11, middle).

Interestingly, two single-nucleotide changes in the gene encoding the regulatory subunit of PKA (PKAr; PF3D7_1223100) were also selected with the NPD series. These introduced a phenylalanine-to-isoleucine substitution at position 375 (Ttt/Att) or a glycine-to-valine substitution at position 376 (gGa/gTa). These residues are at the N terminus of a critical signature motif within one of the two predicted cAMP-binding domains of PKAr (fig. S11, bottom).

**Fig. 4.**
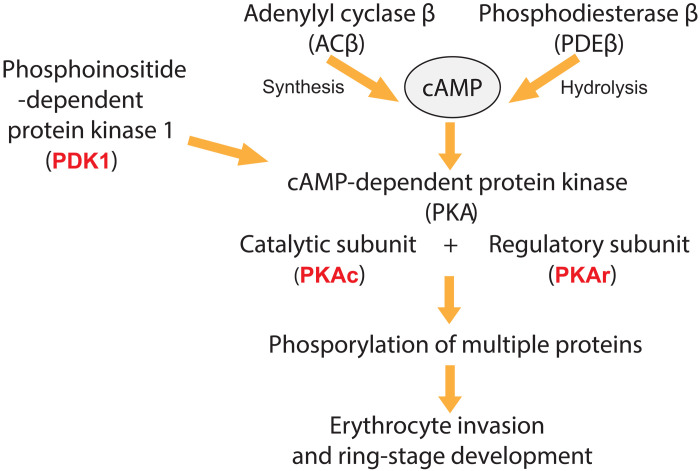
In vitro selection of parasites with mutations in three effectors of cAMP signaling that operate downstream of PDEβ. Schematic showing the key players in the *P. falciparum* cAMP signaling pathway with red indicating pathway components that had mutations selected under drug pressure.

We therefore hypothesize that the mutations (in PKAc, PKAr, or PDK1) selected under drug pressure reduce the activity of PKA to counteract the effects of elevated cellular cAMP levels caused by inhibition of PDEβ (Fig. 4). Table 4 summarizes the mutations selected with all compound series.

These experiments provide strong additional evidence that the target of the three subseries in ABSs is PDEβ and that ABS parasite killing results from elevated cAMP levels and PKA hyperactivation. The results suggest that mutations in the target itself (PDEβ) cannot readily be selected in vitro. Analysis of a database containing the available whole-genome sequences from 6769 *P. falciparum* isolates (mainly from the MalariaGEN Pf3k project) showed that there were only 10 nonsynonymous single-nucleotide polymorphisms in the PDEβ catalytic domain (comprising 215 amino acids). None of these were at positions highly conserved between PDE enzymes, and the frequency of these changes was quite low. This analysis indicates that the integrity of the catalytic domain is important and supports the notion that mutations in PDEβ may be difficult to select under drug pressure.

Analysis of the MalariaGEN database also revealed that there are no preexisting mutations in the available >6000 *P. falciparum* genomes that correspond to those selected in PKAc and PDK1 using PDE inhibitors, implying that parasite populations now circulating would be fully susceptible to PDEβ inhibitors.

### Analysis of transgenic parasites engineered to express the selected mutations in PKAc and PDK1 confirmed their causal role in mediating resistance

Although whole-genome sequencing strongly implicated mutations in PKAc and PDK1 in the resistance phenotype, it was important to rule out a possible contribution of additional genetic changes in the selected parasites. To address this, we generated transgenic parasites expressing the selected PKAc Cca/Tca (P305S) or PDK1 aAt/aTt (N324I) mutations using CRISPR-Cas9 gene editing in the parental Dd2 background. Plasmid maps and oligonucleotides used to generate these transgenic lines are shown in fig. S12 and table S5. Determination of EC_50_ values for the three subseries using cloned edited parasite lines confirmed that resistance was caused by either of these mutations. Fold increases in EC_50_ values observed in these reverse genetic clones, compared to parental Dd2 parasites, were similar to or higher than those of the forward genetic cloned lines originally selected under drug pressure (fig. S13A). These experiments also demonstrated that either of these PKAc and PDK1 mutations could mediate cross-resistance between the three PDE inhibitor series. For example, the PDK1 mutation selected by the 5-benzyl compound (SAL-0010003) also mediated resistance to the 5-aryl and 2-alkyl compounds. Similarly, the PKAc mutations selected by the 5-aryl (SAL-0010355) and 2-alkyl (SAL-0010419) compounds also mediated resistance to the 5-benzyl compound (fig. S13A). Consistent with these results, the PKAc (P305S) and PDK1 (N324I) mutations in the edited parasites also mediated cross-resistance to the independently developed NPD inhibitor series, with similar EC_50_ shifts (fig. S13A). The converse experiments showed that mutations in the resistant clones selected with the NPD series mediated cross-resistance to the 2-alkyl, 5-aryl, and 5-benzyl series (fig. S13B). Table S6 summarizes the changes in inhibitor susceptibility of lines selected under drug pressure compared to the parental line. Together, these data strongly support our hypothesis that the selected mutations in PKAc, PKAr, or PDK1 can compensate for elevated cAMP levels stimulated by inhibition of PDEβ.

## DISCUSSION

Here, we report three chemical subseries that target *P. falciparum* PDEs and have potent activity against both ABS and sexual-stage parasites. The compounds have a fast to moderate speed of kill against ABS parasites where their mechanism of action is through inhibition of cAMP and cGMP hydrolysis. In mature gametocytes, their mechanism of action is through inhibition of cGMP hydrolysis only. We found that many of our compounds had good levels of selectivity against human PDE5 and PDE6, the human PDE isoforms most relevant to our structures. Representative compounds are also active against drug-resistant *P. falciparum* clinical isolates from Africa and South America and against Dd2, a multidrug-resistant laboratory isolate, indicating that cross-resistance in existing parasite populations should not be an issue with this novel mechanism of action. In vitro resistance selection studies yielded parasites with reduced susceptibility (~5- to 15-fold) to these PDE inhibitor series. Whole-genome sequencing found no mutations in PDEβ but, instead, revealed mutations in three genes involved in cAMP signaling, providing additional strong evidence that PDEβ, the only PDE isoform expressed in ABS parasites that can hydrolyze cAMP, is the target of our compounds. These data suggest that ABS killing may rely more heavily on inhibition of cAMP hydrolysis (causing elevated cAMP levels and hyperactivation of PKA) than on inhibition of cGMP hydrolysis. Future work will investigate this further.

### PDEs as antimalarial drug targets

There is a strong precedent for targeting PDEs safely to treat multiple human health conditions. For instance, the human PDE5 inhibitors, sildenafil and tadalafil, are available “over-the-counter” drugs for noninfectious conditions (*17*). Human PDE4 inhibitors are also approved for human use (*32*); roflumilast is used to treat chronic obstructive pulmonary disease (*33*), and apremilast is used to treat psoriatic arthritis and plaque psoriasis (*34*). Inhibitors of several of the 11 human PDE subfamilies are in clinical trials to treat a range of diseases (*35*, *36*).

Reverse genetics studies have revealed the essential nature of three of the four *Plasmodium* PDEs. PDEβ is essential for ABS development (*18*), PDEδ is essential for sexual development (*19*, *22*, *24*), and PDEγ is essential for sporozoite motility that is necessary for transmission from mosquitos to humans (*23*). Thus, *Plasmodium* PDEs are interesting targets for antimalarial drug discovery. It has been known for some time that several human PDE inhibitors (such as caffeine, 3-isobutyl-1-methylxanthine, rolipram, and theophylline) are inactive against malaria parasites and with IC_50_ values of >100 μM (*21*, *37*) against PDE activity in cell extracts. The human PDE5 inhibitors sildenafil and tadalafil also have poor activity against malaria parasites, with IC_50_ values in the mid-to-high micromolar range (*37*, *38*). Another human PDE5/PDE6 inhibitor, zaprinast, has more potent activity against malaria parasites and *Plasmodium* PDEs and has served as a valuable research tool to investigate apicomplexan biology. The reported median effective dose value for zaprinast in a *P. falciparum* ABS growth inhibition assay was 35 μM (*21*).

A previous homology modeling approach determined that *P. falciparum* PDEs were the most similar to human PDE1 and PDE9. This led to the finding that human PDE1/PDE9 inhibitors have better potency against malaria parasites than zaprinast. That work identified a compound, 5-benzyl-3-isopropyl-1*H*-pyrazolo[4,3-d]pyrimidin-7(6*H*)-one (BIPPO) (*39*, *40*), not only with an EC_50_ value in a *P. falciparum* ABS growth inhibition assay of 400 nM but also with high potency against recombinant human PDE9 (30 nM). This molecule has also proved to be an important research tool to investigate the role of cyclic nucleotide signaling in apicomplexan parasites. Both zaprinast and BIPPO have been shown to have activity against PDEα (*20*, *21*, *39*), and we have measured an IC_50_ of 9 nM for BIPPO against PDEβ. A human PDE inhibitor repurposing project found that a tadalafil analog had an EC_50_ of 500 nM against *P. falciparum* ABS growth (*41*). These analogs retained high levels of activity against human PDE5, but the situation was improved following additional synthesis (*42*). Before our study, BIPPO and the tadalafil analogs were the reported PDE inhibitors with the most potency against malaria parasites, although a very recent medicinal chemistry study has generated more potent analogs of BIPPO (*31*) that are also likely to be PDE inhibitors.

### The mechanism of action of the compounds in ABS parasites is through inhibition of PDEβ

In vitro drug pressure experiments performed with PDE inhibitors did not select parasites with mutations in PDEβ, and so it is possible that mutations in the inhibitor-binding pocket, which is also the cyclic nucleotide–binding pocket, cannot be tolerated. However, our in vitro experiments were able to select parasites with ~5- to 15-fold reduced susceptibility to the PDE inhibitors. Whole-genome sequence analysis identified mutations in three genes encoding proteins known to be involved in cAMP signaling: PKAc, PKAr, and PDK1. This leads to the hypotheses that elevated cAMP levels, caused by inhibition of PDEβ and consequent hyperactivation of PKA, are responsible for ABS parasite killing and that mutations in any of these three genes compensate for the effects of the inhibitors in vitro. This hypothesis is supported by a recent study on *P. falciparum* PDK1, which showed that overexpression of PKAc led to selection of parasites with mutations in PDK1 (*14*). This and targeted mutagenesis identified PDK1 as a critical regulator of PKA activation, through phosphorylation of the activation loop threonine (Thr^189^) of PKAc, a known mechanism for PKA regulation in higher eukaryotes (*43*). These findings also demonstrated that attenuating mutations (close to the adenosine triphosphate–binding pocket) in PDK1 reduced the lethal effects of PKA overexpression (*14*), allowing survival. The lethal effects of PDE inhibitors are therefore similar to those of PKAc overexpression.

Whether the mutations selected under PDE inhibitor pressure confer a fitness cost in ABS parasites and whether parasites with these mutations can form gametocytes and transmit to mosquitoes will be the subject of future work to further evaluate the likelihood of these mutations being selected in a clinical setting. Note that gametocytes expressing the selected PKA mutations (which confer reduced susceptibility to PDEβ inhibitors in ABS parasites) are predicted to remain fully susceptible to PDE inhibitors additionally targeting PDEδ because killing at this life cycle stage is through elevated cGMP levels and premature PKG activation. The distinct mode of action of PDE inhibitors in ABS parasites and gametocytes has important implications for the potential spread of resistance.

In vitro drug selection experiments have been previously used successfully to identify the targets of novel *P. falciparum* ABS inhibitor series (*2*–*4*). This raises the question of why PDEβ mutants were not selected in these experiments if PDEβ is the target of the inhibitors. One explanation, as mentioned above, is that these mutants cannot be readily selected because of the necessity of maintaining the integrity of the highly conserved cyclic nucleotide–binding pocket for ABS parasite survival. The three genes (*PKAr*, *PKAc*, and *PDK1*) in which mutations were selected in the presence of PDE inhibitors are not the targets of the inhibitors as the observed effects on cyclic nucleotides are upstream of the cognate proteins. Rather, they encode downstream effectors of cAMP signaling and carry mutations that likely partly compensate for PDEβ inhibition. There is a precedent for an in vitro drug selection experiment that was unable to generate parasites with a mutation in the compound target, PKG (*44*). In that study, a mutation in a distinct protein (TKL3) mediated low-level resistance but was not itself a target of the inhibitor. Presumably, the selected mutation partly compensates for the effects of the PKG inhibitor. It has also been reported that in vitro drug pressure with Hesperadin, which inhibits the human aurora kinase Ark1, led to selection of a mutation in a nontarget kinase, Nek1 (*45*).

### Inhibition of cGMP hydrolysis is also likely to contribute to the mechanism of action

The strong correlation observed for the three chemical series between activity against ABS parasites and against cAMP hydrolysis by PDEβ in schizont extracts, along with the selection of parasites harboring mutations in downstream effectors of cAMP signaling under drug pressure, demonstrates that PDEβ is the primary target of the inhibitors. However, since PDEβ can hydrolyze both cAMP and cGMP, this leaves unanswered the question of what the contribution of inhibition of cGMP hydrolysis is to ABS parasite killing. No mutations were selected under drug pressure in downstream effectors of cGMP signaling. It is very likely that interfering with cGMP signaling contributes to the ABS killing mechanism of PDE inhibitors, but this may be secondary to the effects of interfering with cAMP signaling. We have shown that conditional knockout of PDEβ leads to a 70% reduction of *P. falciparum* blood-stage merozoite invasion, and those parasites that could invade were found to die at the early ring stage (*18*). Chemical genetics and conditional knockout of PKG, on the other hand, lead to a block in ABS egress (*10*, *11*). Furthermore, the potent effects of PKG inhibitors on ABS egress are also well documented (*5*, *10*, *12*, *44*). It cannot be ruled out, however, that compensatory mutations in PKG, the only known downstream effector of cGMP signaling, cannot be tolerated by the parasite and that inhibition of cGMP hydrolysis makes an important contribution to ABS parasite killing. In support of this possibility, it has been reported that it is relatively difficult to generate parasites that are resistant to PKG inhibitors in vitro (*5*, *44*). While the effects of PDE inhibitors on cAMP signaling can easily be attributed to inhibition of PDEβ (as it is the only ABS PDE that can hydrolyze cAMP), the situation regarding the effects of PDE inhibitors on cGMP signaling is more complicated. This is not only because PDEβ can hydrolyze both cAMP and cGMP but also because a second enzyme, PDEα that is expressed in ABS parasites, can also hydrolyze cGMP. IC_50_ values for our compounds for inhibition of cAMP and cGMP hydrolysis in ABS parasite extracts were generally similar, suggesting that they inhibit both PDEβ and PDEα. This conclusion is supported by our measurement of elevated levels of both cAMP and cGMP in inhibitor-treated ABS parasites. PDEα has previously been shown to be dispensable for *P. falciparum* ABSs (*20*), presumably due to the presence of PDEβ in the mutant parasites. In summary, it is likely that the effects of PDE inhibitors on ABS parasites are mediated by interference with both cAMP and cGMP hydrolysis, although the relative contributions are difficult to dissect.

In gametocytes, our data indicate the mechanism of action being through inhibition of cGMP hydrolysis. First, elevated cGMP levels can be measured in inhibitor-treated gametocytes with no change in the levels of cAMP. In addition, previous transcriptome data have shown that PDEδ and PDEα are the only isoforms expressed in gametocytes (https://plasmodb.org), and published work using recombinant expression of PDEα and knockout of PDEα and PDEδ have pointed to them both being cGMP specific (*19*–*22*, *24*). A recent paper, however, reported that the catalytic domain of the *P. falciparum* PDEδ expressed in *Escherichia coli* was able to hydrolyze both cAMP and cGMP (*25*). Currently, we do not know the explanation for the difference in findings on the cyclic nucleotide specificity of PDEδ between the studies. It is possible that when the catalytic domain of PDEδ is expressed in *E. coli* in the absence of its N-terminal regulatory domain, it is able to hydrolyze cAMP, although we have found no evidence that the full-length protein in its cellular context can do this. Another possibility is that the levels of cAMP in mature gametocytes are low and difficult to detect. However, using immunoprecipitated HA-tagged PDEδ, we provide strong evidence suggesting that PDEδ can only hydrolyze cGMP.

In addition to the effects on gamete formation, the PDE inhibitors also affect the development of stage IV and V gametocytes. A drug would have a far greater likelihood of transmission blocking activity if it kills gametocytes in the human bloodstream, rather than only acting against gamete formation in the mosquito. Demonstration of activity of the 5-benzyl and 5-aryl series in gametocyte viability assays indicates that PDE activity is required in stage IV and V gametocytes to regulate cGMP levels to prevent premature activation of PKG and stimulation of gamete formation when gametocytes are still in the human host. This is supported by our finding that PDE inhibitors not only lead to elevated cGMP levels but also stimulate gametogenesis in a PKG-dependent manner, in the absence of the other known stimulators XA and reduced temperature. The addition of PDE inhibitors effectively bypasses the requirement for these environmental stimulants. For transmission to be successful, gamete formation must occur only after the gametocytes enter the mosquito during a blood meal, and so it is likely that PDE inhibitors would disrupt the role of PDEδ/α as the “off switch” that prevents PKG activation in the human host to keep gametocytes “dormant.”

### Selectivity against human PDE5 and PDE6 has been improved by chemical synthesis

The chemical start points in our study were derived from Pfizer chemistry programs aiming to optimize activity against human cGMP-specific PDEs. It was therefore expected that they would have high levels of activity against their intended target. Unexpectedly, some of the initial human PDE5 inhibitors that we tested were more potent inhibitors of malaria parasite PDEβ activity than the human PDE isoform against which they were designed. This, combined with the marked amino acid sequence differences between human and parasite PDE isoforms at key positions in the catalytic domain (*18*), gave optimism that good levels of selectivity might be achievable. Some of the compounds developed during this study have a selectivity window of >100 over human PDE5 and/or PDE6. Two over-the-counter drugs (sildenafil and tadalafil) that target human PDE5 have considerable off-target activity against PDE6 (<10-fold selectivity) and PDE11 (~5-fold selectivity), respectively (*46*).

In summary, we report on compounds with potent activity against *P. falciparum* ABS and sexual-stage parasites, mediated by inhibition of stage-specific PDE enzymes. Some of the compounds exhibit excellent levels of selectivity against human PDE isoforms with the promise of a good safety profile. In vitro selections yielded parasites with mutations in protein kinases that operate downstream of PDEβ to mediate cAMP signaling, but parasites with mutations in PDEβ itself, the target in ABS, were not selected. Future work will aim to further improve the properties of our hit compounds to progress a PDE inhibitor further along the drug discovery pipeline.

## MATERIALS AND METHODS

### Chemistry strategy summary

Before this study, a high-throughput screen of a diverse subset of Pfizer’s compound collection of approximately 150,000 was carried out using a blood-stage *P. falciparum* growth inhibition assay at the Eskitis Institute for Drug Discovery, Griffith University, Nathan, QLD 4111, Australia. Counterscreening of activities was carried out in human embryonic kidney–293 cells. Some of the hits were known human PDE inhibitors or had similar structures to known human PDE inhibitors. Some analogs were synthesized to improve potency against *P. falciparum* and improve other drug-like properties. Following on from this initial work, we demonstrated that the series and a diverse set of structural analogs from Pfizer’s compound collection had activity against *P. falciparum* PDEβ. Of the large number of different human PDE inhibitor chemotypes screened, only three subseries had marked activity against *P. falciparum* PDEs. The most promising hits were then used as the start points for the present study.

The chemistry start points for this work were SAL-0010042 (example 76 in WO2003037899, 5-benzyl), SAL-0010043 (CAS 155879-55-3, 5-aryl), and SAL-0010034 (CAS 335076-55-6, 2-alkyl) (fig. S1A). Full details of the medicinal chemistry will be published in a separate paper (in preparation). Some characteristics of an ideal *P. falciparum* PDE inhibitor would include potent activity against PfPDEβ and PfPDEδ, excellent selectivity over human PDEs, and properties commensurate with a single low human therapeutic dose cure of malaria such as good solubility, absorption, and metabolic stability.

The addition of a para-F to SAL-0010042 (5-benzyl series) gave SAL-001003 (Table 1 and fig. S2), a compound with very similar properties and the potential for improved metabolic stability through (putative) metabolism blocking. A more substantial structural change is present in SAL-0010007 where the conformation of the 5-benzyl substituent has been constrained by cyclization. The activity of this analog is similar in the schizont cAMP hydrolysis inhibition assay and 3D7 ABS growth inhibition assay. It also shows markedly increased selectivity over human PDE6 compared to the uncyclized analogs. Activity in the gametocyte cGMP hydrolysis inhibition assay is reduced.

The starting point for the 5-aryl series (the name we give for 2*H*, 5-aryl compounds) is a pyridopyrimidinone. However, from other SAR, we knew that the pyridyl ring could be exchanged for the pyrazole ring present in the other leads with benefits in synthetic ease and metabolic stability, so we focused on that ring system. des-Me sildenafil contains that ring switch and demonstrates equal activity in the 3D7 growth inhibition assay. The compounds containing larger alkoxy chains present in SAL-0010203 and SAL-0010243 demonstrate improved potency against the enzyme and parasite. The 5-aryl ring can be phenyl or pyridyl (α to the alkoxy substituent) and tolerates a range of substitution para to the alkoxy substituent (sulfonamides, amides of various lengths, and bromine). Unfortunately, these changes do not confer any substantial level of selectivity over human PDE5 or PDE6. This series is less active against PfPDEδ/PfPDEα in gametocytes, being approximately 10-fold less active, or weaker, than against PfPDEβ in schizonts.

With similar substituents at the 5-aryl position, substitution at the 2-position decreases activity slightly against PfPDEβ and in the 3D7 ABS parasite growth inhibition assay (compare, for example, the pairs SAL-0010203 5-aryl with SAL-0010039 2-alkyl and SAL-0010301 5-aryl with SAL-0010031 2-alkyl) (Table 1 and fig. S2). However, the 2-alkyl series show pronounced selectivity over human PDE5 and PDE6 (and very little activity against PfPDEδ/PfPDEα in gametocytes). Analytical data are shown in data S1.

### EC_50_ determination for growth inhibition of *P. falciparum* using an LDH-based assay

*P. falciparum* isolates (3D7/Dd2) were obtained from BEI Resources and were cultured using standard procedures as described elsewhere (*47*). Briefly, *P. falciparum* was grown in RPMI 1640 medium (10.3 g/liter) supplemented with l-glutamine (2 mM), hypoxanthine (150 μM), Hepes (25 mM), d-glucose (12 mM), sodium bicarbonate (25.7 mM), AlbuMAX II (0.5% or 5 g/liter) (pH 7.4), and 2% RBC (type O+, freshly prepared). Cultures were replenished with fresh medium and RBCs every other day. For RBC preparation, whole blood was centrifuged at 415*g* at room temperature for 12 min. After that, the plasma and buffy coat were removed, and packed RBCs were washed three times with 3 vol of RPMI 1640. Last, RBCs were resuspended in 1:1 vol of complete RPMI 1640 medium (containing 0.5% AlbuMAX II).

The *P. falciparum* 3D7 and Dd2 LDH growth inhibition assay was carried out as described elsewhere (*48*) with minor modification of the parasitological conditions (10 to 15% parasitemia with ≥80% rings). Data were normalized to percent growth inhibition with respect to positive (0.2% DMSO as 0% inhibition) and negative (for 3D7, a mixture of 100 nM chloroquine and 100 nM atovaquone as 100% inhibition; for Dd2, a mixture of 200 nM DHA and 200 nM atovaquone as 100% inhibition) controls.

### Cyclic nucleotide hydrolysis assay

The cyclic nucleotide hydrolytic activity of membrane-associated parasite PDE enzymes was measured by a scintillation proximity assay (SPA) (*18*) using lysates of mature *P. falciparum* schizonts or stage IV/V gametocytes. Briefly, *P. falciparum* 3D7 schizonts or stage IV/V 3D7/GDV1 (*49*) gametocytes in culture were collected by purification in 70% Percoll, followed by saponin lysis, resuspension in 5 mM tris-HCl (with EDTA-free protease inhibitors), and centrifugation at 16,000*g* for 10 min at 4°C to remove the RBCs until the supernatant was clear. The pellets were then resuspended in 250 μl of PDE lysis buffer [10 mM tris-HCl (pH 7.5), 150 nM NaCl, 0.5% NP-40, and EDTA-free protease inhibitors] per 50 μl of sample and incubated on ice for 30 min with occasional mixing. Supernatants containing the membrane lysates were collected after 20 min of centrifugation at 16,000*g* at 4°C and diluted in PDE assay buffer [10 mM tris-HCl (pH 7.4), 10 mM MgCl_2_, bovine serum albumin (0.1 mg/ml), and 0.05% Tween 20]. For the PDE SPA assay, a dilution of the lysate sufficient to obtain the 30% of the maximum hydrolytic activity was used. The IC_50_ values for the compounds were determined using threefold serial dilutions in duplicate. The assays were carried out in flexible 96-well plates (PerkinElmer, 1450-401) by incubating lysates and compounds with 5 μl of 200 nM cNMP dilution [[^3^H]-cNMP tracer (PerkinElmer, cAMP-NET275250UC and cGMP-NET337250UC) in PDE assay buffer] for 1 hour at 37°C to a total volume of 50 μl. The reactions were terminated by addition of 25 μl of PDE SPA beads (PerkinElmer, RPNQ0150), and after 20 min of incubation at room temperature, scintillation was measured using a Wallac 1450 Microbeta Counter (PerkinElmer) for 30 s. Dose-response curves were fitted in a four-parameter logistic regression model to obtain the IC_50_ values for each compound.

### Immunoprecipitation of PDEβ-HA

A transgenic PDEβ-HA tag line of *P. falciparum* 3D7 generated previously (*18*) was used to pull-down PDEβ-HA using anti-HA affinity beads (Pierce Anti-HA Magnetic Beads, 88836). Briefly, schizont pellets were obtained, and membrane lysates were prepared as describe above. A total of 25 μl of anti-HA affinity beads were washed with PDE lysis buffer to equilibrate them. The lysate was added to the equilibrated anti-HA affinity beads and incubated for 2 hours in a rotor at room temperature. After incubation, the samples were placed on a magnet to remove the supernatant and washed three times with ice-cold dilution buffer. Last, the beads were resuspended in 500 μl of PDE assay buffer and used for the PDE assay.

### Measurement of intracellular cyclic nucleotide levels

cGMP and cAMP levels in *P. falciparum* 3D7 ring stages, mature schizonts, and gametocytes were measured using an ELISA-based high-sensitivity direct cGMP and cAMP colorimetric assay kits (Enzo Life Sciences). Mature schizonts were Percoll purified from synchronous *P. falciparum* 3D7 cultures, followed by resuspension and lysis in 0.1 M HCl solution. All samples were pelleted at 10,000*g*, and the supernatant was collected and stored at −80°C until required. For ring-stage measurements, mature schizonts were Percoll purified and returned to culture flasks containing fresh erythrocytes in complete medium with continuous shaking for 4 hours to allow for reinvasion. Newly formed ring stages were purified by saponin lysis, and the phosphate-buffered saline–washed pellets were lysed and stored similarly to schizonts. *P. falciparum* 3D7/GDV1 parasites were used for gametocyte production (*49*). GDV1 expression in ring-stage parasites were induced with Shield-1, and after reinvasion, cultures were treated with 50 mM *N*-acetyl-d-glucosamine for 8 days to eliminate asexual parasites. On day 14, late stage IV/V gametocytes were treated for 15 min with 10× IC_50_ of one compound from each subseries or 100 μM XA. Gametocytes were then harvested and Percoll purified, followed by saponin lysis to eliminate the RBC. Pellets were resuspended and lysed in 0.1 M HCl solution. To perform the ELISA, samples and standards were acetylated to improve detection sensitivity according to the manufacturer’s instructions. Standards and samples were run in triplicate on the same plate and absorbance measured at 405-nm read with a SpectraMax iD5 plate reader. The standard was fitted to a sigmoidal curve and used to determine cyclic nucleotide concentrations in parasite samples.

### Determination of the effects of SAL-0010042 on *P. falciparum* ABS parasite growth

*P. falciparum* 3D7 ring-stage parasites synchronized to a 2-hour invasion window were adjusted to a 1% parasitemia and 1% hematocrit suspension and dispensed in triplicate into a 24-well plate for each of the five treatment conditions. Wells were treated with 0.1 μM SAL-0010042 in triplicate at the specified time points, and triplicate wells were also treated with the equivalent volume of DMSO at 2 hours after invasion. Samples of 100 μl were collected from wells at 44 hours after invasion and fixed with 4% formaldehyde 0.2% glutaraldehyde in phosphate-buffered saline. Fixed samples were stained with SYBR green (Thermo Fisher Scientific) and analyzed by flow cytometry on an Attune NxT flow cytometer (Thermo Fisher Scientific). Schizont parasitemia was determined by gating high-signal SYBR-positive cells.

### PRR assay

The in vitro killing profile of compounds was determined by monitoring the ability of parasites to recover following treatment with a compound of interest using a previously reported approach (*50*), which allowed calculation of the PRR (*51*). EC_50_ values for the compounds were first determined using a *P. falciparum* 72-hour LDH assay as previously described (*50*). *P. falciparum* (clone 3D7) cultures were then incubated with an example of the three PDE inhibitor subseries (5-benzyl, SAL-0010003; 2-alkyl, SAL-0010031; 5-aryl, SAL-0010301) at 10× EC_50_ for 120 days. Fresh compound was added daily; parasite samples were taken after 0, 24, 48, 72, 96, and 120 hours; and after washing out the compound, the samples were cultured in 96-well plates with fresh human RBCs, in the absence of compound. To quantify the number of viable parasites following treatment, four independent sets of threefold serial dilutions were performed on each sample, which were assayed for the presence or absence of parasites after 28 days using the LDH assay, and the number of viable parasites was determined by the number of wells showing growth. PRR and PCT_99.9%_ were calculated as previously reported (*50*).

### Induction of gametogenesis

Mature (>90% stage V) gametocytes were exposed to short-term treatment with two of the 5-benzyl series compounds, SAL-0010042 and SAL-0010003. In the absence of any other stressors (drop in temperature, pH increase, and XA), mature gametocytes were treated at 1× IC_50_ of either compound, and samples were taken at 15, 30, and 60 min. Giemsa-stained slides were prepared at these time points, and the parasite populations were evaluated morphologically using light microscopy. Male and female gametes were identified or distinguished on the basis of rounding up, shape, and size. Statistical evaluation was performed with paired, two-tailed *t* test (GraphPad Prism 6.0).

### Exflagellation inhibition assay

The exflagellation inhibition assay was performed by capturing movement of exflagellation centers over time by video microscopy [adapted from a reported protocol (*52*)]. For this carry-over format, mature stage V gametocytes were treated with 2 μM of each compound for 48 hours before induction of exflagellation in the presence of compound. To evaluate compound dose response, gametocytes were treated similarly at a set of concentrations representing a fourfold serial dilution. Mature gametocyte cultures (>95% stage V; 500 μl) were centrifuged at 3500 rpm for 30 s, and the pellet was resuspended in 30 μl of ookinete medium [RPMI 1640 medium containing l-glutamine (Sigma-Aldrich, R6504), gentamycin (0.024 mg/ml; HyClone, SV30080.01), 202 μM hypoxanthine (Sigma-Aldrich, H9636), 25 mM Hepes (Sigma-Aldrich, H4034), 0.2% glucose (Sigma-Aldrich, G6152), and 0.5% (w/v) AlbuMAX II (Invitrogen, Paisley, UK), supplemented with 100 μM XA and 20% human serum]. The activated culture (10 μl) was introduced into a Neubauer chamber and placed on the microscope platform to settle homogenously. Time was noted as time zero (T0), and the chamber was incubated at room temperature. Movement was recorded by video microscopy using a Carl Zeiss NT 6V/10W Stab microscope, fitted with a MicroCapture camera at 10× magnification, and then quantified by a semiautomated method, a modification of the method described previously (*53*). A series of 15 videos of 8 to 10 s each were captured at random locations between 15 and 22.5 min after incubation. Each video was analyzed using Icy bioimage analysis software to quantify the number of exflagellating centers. Compound IC_50_ values were determined with nonlinear curve fitting (GraphPad Prism 6), normalized to maximum and minimum inhibition.

### Female gamete formation inhibition assay

Mature gametocytes (>95% stage V) were treated with compounds (2 μM) for 48 hours before stimulating female gamete formation. To evaluate compound dose response, gametocytes were treated similarly at set concentrations representing a fourfold serial dilution. Female gamete activation was induced by both a temperature drop and the addition of 100 μM XA. Monoclonal anti-Pfs25 antibody (BEI Resources, catalog number MRA-28; 1:1000 dilution) conjugated to fluorescein isothiocyanate was used to detect female gametes. Image acquisition was performed using a Zeiss Axio Lab.A1 epifluorescence microscope with a 100/1.4 numerical aperture oil immersion objective and a Zeiss Axiocam 202 mono digital camera. Using a 100× objective, 30 images were taken per sample and analyzed manually. The number, size, roundness, and intensity of fluorescence of activated female gametocytes were evaluated. Compound IC_50_ values were determined with nonlinear curve fitting (GraphPad Prism 6), normalized to maximum and minimum inhibition.

### LDH-based gametocyte viability assay

The pLDH assay was performed on late (>95% stage IV/V) gametocytes. Compound dilutions were placed in triplicate in 96-well plates in a final volume of 100 μl per well. DHA and MMV048 were used as positive drug controls. The gametocyte cultures (100 μl per well) were added to the 96-well plates to achieve a final gametocytemia and hematocrit of 2 and 1%, respectively, in a total incubation volume of 200 μl. Plates were incubated at 37°C for 72 hours, followed by the replacement of spent medium with drug-free culture medium (75% medium change) (*54*, *55*). Plates were then incubated for a further 72 hours before assessing viability by measuring pLDH activity. Gametocyte viability was determined spectrophotometrically by measuring the activity of pLDH, according to a modified version of a published protocol (*56*). Briefly, 100 μl of Malstat reagent [0.21% (v/v) Triton X-100, 222 mM l-(+)-lactic acid, 54.5 mM tris, 0.166 mM APAD (Sigma-Aldrich, A5251), adjusted to pH 9 with 1 M NaOH] was transferred into a clean 96-well plate. A fixed volume of 20 μl of parasite suspension per well was added to the Malstat plate, followed by the addition of 25 μl of PES/NBT (0.239 mM phenazine ethosulfate and 1.96 mM nitro blue tetrazolium chloride). Absorbance was measured with a Multiskan Ascent 354 multiplate scanner (Thermo LabSystems, Finland) at 620 nm.

### Initial SMFA and sporozoite production

*P. falciparum* NF54 gametocytes were prepared for blood-feeding to mosquitoes according to the method described (*57*), with some slight modifications. Briefly, gametocyte cultures seeded at 2% overall parasitemia and 4% hematocrit were maintained at 37°C for 16 days with daily medium changes. Gametocyte functional viability was assessed by an exflagellation assay at day 14 after induction, observed by light microscopy. *Anopheles stephensi* (SD500 strain) were reared as described (*58*). Gametocyte aliquots from a single NF54 culture, produced as above, were incubated either with SAL-0010003 or SAL-0010042 in two separate experiments at 100 nM, 500 nM or 1 μM, 1 μM methylene blue (full-block control), or culture medium only (no-drug control) from day 14 to day 16 when SMFA was carried out. Fresh drug-containing medium was provided each day during this exposure. Pots containing 70 to 80 female *A. stephensi* mosquitoes, aged 2 to 5 days old, were allowed to feed on 500 μl of the respective culture plus drug mixture, presented to each pot in a preheated three-dimensional printed water channel membrane feeder (*59*), until fully fed. Mosquitoes were placed in an incubator at standard conditions. Seven to 8 days after the feed, 10 to 15 mosquitoes were removed and kept under standard conditions for salivary gland dissection on day 14 after feeding. The remaining mosquito midguts were dissected on day 9 or day 7, respectively, after the feed in 0.25% mercurochrome stain to determine oocyst counts by light microscopy. Salivary gland dissections were conducted on day 14 after the feed, and the presence of sporozoites was noted for each dissected mosquito by light microscopy.

### SMFA dose response

Compounds were diluted in DMSO and then in culture medium (RPMI 1640 supplemented with 367 μM hypoxanthine, 25 mM Hepes, 25 mM sodium bicarbonate, and 10% human type A serum) to achieve a final DMSO concentration of 0.1%. Compounds were incubated with stage V gametocytes from the *P. falciparum* NF54-HGL reporter strain (*60*) for 48 hours at 37°C in Eppendorf tubes. Subsequently, parasites were pelleted by centrifugation (20 s at 20,000*g*) and resuspended in human type A serum containing diluted compound (final DMSO concentration of 0.1%) and human type O RBCs to achieve a hematocrit of 50%. This blood meal was then fed to 3- to 5-day-old *A. stephensi* mosquitoes [Sind-Kasur Nijmegen strain (*61*)] for 10 min. Following feeding, unfed mosquitoes were removed, and mosquitoes were maintained at 26°C and 70 to 80% relative humidity. Eight days after feeding, oocyst intensity and prevalence were assessed by luminescence measurement of individual mosquitoes as described (*60*). All conditions were tested in duplicate, and for each replicate, a total of 24 mosquitoes were analyzed. IC_50_ values were estimated by fitting a four-parameter Hill model to the data using least squares to find the best fit. Controls included vehicle (0.1% DMSO) and 10 μM DHA.

### Ex vivo isolates from Tororo, Uganda

This study received approval from UCSF-CHR (16-19084), SBS-REC (SBS-341), and UNCST (HS 2018) dated 23 March 2016; isolate collection dates were October 2020 to January 2021. Clinical isolates were from the Tororo District of Uganda, located in the central-eastern part of the county near the Kenya border. Tororo and surrounding areas have high-intensity, year-round malaria transmission. Parasite samples were obtained from individuals aged 6 months or older presenting to the Tororo District Hospital in the Tororo District (or Masafu Hospital in the Busia District), with clinical symptoms suggestive of malaria and a positive Giemsa-stained blood film for *P. falciparum*. Patients reporting use of antimalarial treatment within the previous 30 days or with evidence of an infection with other *Plasmodium* species were excluded. Informed consent was obtained from patients and/or primary care givers (depending on age); children aged 8 to 17 years provided assent. Two to 5 ml of blood was collected by venipuncture in a heparin tube. Samples were delivered to the adjacent IDRC laboratory at Tororo District Hospital or transported by car from Masafu Hospital on the day of collections for storage of blood spots on filter paper (for isolation of nucleic acids) and initiation of ex vivo culture of malaria parasites. Treatment involved a standard therapeutic regimen for uncomplicated falciparum malaria, which was a 3-day course of the artemisinin-based combination therapy (ACT), artemether-lumefantrine. In Uganda, at the time of sampling, there was widespread resistance to the antifolate drugs due to mutant *pfdhfr*/*pfdhps* alleles. SAL-0010042 and SAL-0010333 were tested for ex vivo potency against fresh clinical *P. falciparum* isolates using a standard 72-hour microplate assay, alongside the two standard antimalarial compounds supplied by Medicines for Malaria Venture (MMV): Pyrimethamine was prepared as a 50 mM stock in DMSO. Chloroquine was prepared at 10 mM in water. Stock solutions were stored at −20°C. Working solutions were freshly prepared from a single aliquot within 4 hours of the susceptibility test and stored at 4°C.

### Ex vivo drug susceptibility assay

Ugandan clinical isolates with a *P. falciparum* ABS parasitemia of ≥0.3% were evaluated for drug susceptibility in an ex vivo 72-hour microplate IC_50_ assay using SYBR Green detection. Samples were delivered to the adjacent IDRC laboratory at Tororo District Hospital or transported by car from Masafu Hospital on the day of collection for storage of blood spots on filter paper (for isolation of nucleic acids) and initiation of ex vivo culture of malaria parasites. Blood was washed three times by centrifugation at 2000 rpm for 10 min with RPMI 1640 medium (Thermo Fisher Scientific) at 37°C. Plasma and buffy coat were removed, and the erythrocyte pellet was resuspended in complete medium consisting of RPMI 1640 supplemented with 25 mM Hepes, 24 mM NaHCO_3_, 0.1 mM hypoxanthine, gentamicin (10 μg/ml), and 0.5% AlbuMAX II (Thermo Fisher Scientific) to produce a hematocrit of 50%. Before assay, 10 mM antimalarial stock compounds were diluted as necessary in DMSO and stored at −20°C. On the day of the assay, 2 μl of DMSO stock drugs were diluted in 498 μl of complete RPMI 1640 medium. Diluted compounds were not stored for more than 24 hours. Compounds were serially diluted threefold in a 96-well assay plate in complete medium to a final volume of 50 μl, in columns 1 to 10, with concentrations optimized to capture full dose-response curves. Column 11 contained compound-free controls, while column 12 contained uninfected human RBC controls. Parasite culture (150 μl) was added to columns 1 to 11 of the assay plate with a final volume of 200 μl per well, 0.2% parasitemia, and 2% hematocrit. Plates were incubated for 72 hours in a humidified modular incubator (Billups-Rothenberg) under a blood gas mixture (95% N_2_, 3% CO_2_, and 2% O_2_) at 37°C. After 72 hours, plates were removed and frozen. Thawed plates were incubated with SYBR Green lysis buffer for 1 hour in the dark as previously reported (*62*). Fluorescence was quantified using a BMG Fluostar Optima plate reader at an excitation of 485 nm and an emission of 530 nm, and data were plotted using GraphPad Prism 7. The data were curve fit with a variable slope function to estimate IC_50_ values. For each isolate, a *Z*′ factor to assess assay quality was calculated from positive controls (eight drug-free wells) and negative controls (eight parasite-free, red cell control wells). *Z*′ values over 0.5 were considered good assays, but each curve was also examined by eye for suitability. Some assays with *Z*′ below 0.5 may be considered valid depending on factors such as the SE of the curve-fit IC_50_. Laboratory control *P. falciparum* Dd2 (MRA-156) and 3D7 (MRA-102) strains (BEI Resources) were maintained in culture, synchronized with a magnetic column (Miltenyi Biotec), and assayed (beginning at the ring stage) once a month.

### Ex vivo isolates from Porto Velho, Brazil

This study was approved by the Centro de Pesquisa em Medicina Tropical (CEPEM) Rondônia ethics committee (CAAE 58738416.1.0000.0011). All participants signed a written informed consent before blood collection by a trained nurse.

*P. falciparum* clinical isolates were collected on March and April 2021 from patients recruited at the Centre of Malaria Control (CEPEM) in the city of Porto Velho, state of Rondônia, in the Brazilian Western Amazon. Only monoinfected patients with *P. falciparum* with parasitemia between 2000 and 80,000 parasites/μl and with at least 70% of ring-stage parasites were recruited. Patients who had used an antimalarial in the previous month and/or presented with symptoms of severe malaria were excluded from the study. The study cohort comprised 24 patients living in this highly endemic area. A peripheral venous blood sample (5 ml) was collected from each individual by venipuncture in heparin-containing tubes and immediately used in the ex vivo drug susceptibility assay using preprepared plates containing diluted antimalarial compounds. The 2-alkyl test compound (SAL-0010255) and the control drugs (artesunate and chloroquine) were prepared from a stock solutions in DMSO at 2 mM (made by dilution of solution A). Next, the compounds were diluted in assay medium, 20,000- and 40-fold, to prepare the initial drug solution of 0.1 μM artesunate and 50 μM chloroquine and SAL-0010255, and then a twofold serial dilution was made in assay medium from this stock. Last, 20 μl of each dilution was transferred into the ex vivo assay plate and diluted 10-fold into the final assay medium containing parasites. For the infected blood preparation, 5 ml of whole blood was centrifuged at 800*g* for 10 min, and the plasma and buffy coat were removed. The RBC pellet was then washed with culture medium (RPMI 1640 medium) and diluted 1× (50% hematocrit) before filtration through a CF11 cellulose column (*63*). After the CF11 cellulose column step, the blood was centrifuged, and the packed RBCs with the parasites [infected red blood cells (iRBC)] were diluted to a 2% hematocrit, using complete medium RPMI 1640 medium plus 20% human serum. Control assays on a 3D7 laboratory isolate were performed with medium supplemented with human serum (20%). The iRBC (180 μl per well) were distributed in the predosed drug plate. For the maturation of parasites, from rings to schizonts, the plates were maintained in a hypoxia incubator chamber (containing 5% O_2_, 5% CO_2_, and 90% N_2_) at 37°C, as described, for 40 to 47 hours. Control wells containing drug-free iRBCs were cultured with complete medium. The parasite-drug incubation was terminated when 40% of the ring stages reached the schizont stage (at least three distinct nuclei per parasite) in the drug-free control wells. Thick blood films were then made from each well, dried, stained with 5% Giemsa solution for 30 min, and examined microscopically. The number of schizonts per 200 asexual-stage parasites was determined for each drug concentration and then normalized by comparing with the schizont number in the drug-free control wells [considered as 100% (*64*)]. The half-maximal drug inhibitory response (EC_50_) was estimated by curve fitting using software from the OriginLab Corporation, Northampton, MA, USA and comparing with parasite growth in the drug-free controls.

### Human PDE5 and PDE6 selectivity assays

The selectivity of the compounds on the parasite PDEs over the two human isoforms PDE5A1 and PDE6C was measured through a dose-response assay of their hydrolytic activity in presence of the compounds. Similarly to the parasite lysates, an SPA was carried out with the recombinant purified human glutathione *S*-transferase–tagged PDE5A1 or PDE6C (BPS Bioscience, BPS-60050 and BPS-60062) prepared in PDE assay buffer [10 mM tris-HCl (pH 7.4), 10 mM MgCl_2_, bovine serum albumin (0.1 mg/ml), and 0.05% Tween 20] at a concentration of 648 pg of enzyme per 50 μl of total volume per well. To determine the IC_50_ of the compounds, threefold serial dilutions in duplicate were performed. Briefly, the assays were carried out in flexible 96-well plates (PerkinElmer, 1450-401) by incubating the enzyme with the compounds and 5 μl of 200 nM cGMP dilution [[^3^H]-cNMP tracer (PerkinElmer, cGMP-NET337250UC) in PDE assay buffer] to a total volume of 50 μl for 45 min at 37°C. The reactions were terminated by addition of 25 μl of PDE SPA beads (PerkinElmer, RPNQ0150), and after 20 min of incubation at room temperature, scintillation was measured using a Wallac 1450 Microbeta Counter (PerkinElmer) for 30 s. Dose-response curves were fitted in a four-parameter logistic regression model to obtain the IC_50_ values for each compound. The selectivity of the compounds was expressed as the fold change in the IC_50_ obtained for PDE5A1 or PDE6C and the IC_50_ for the *P. falciparum* PDEβ schizont lysates.

### In vitro selection of drug-resistant parasites

Stocks of SAL-0010003 (5-benzyl), SAL-0010355 (5-aryl), and SAL-0010419 (2-alkyl) were made at 10 and 1 mM in DMSO. Aliquots in use were stored at −20°C, and long-term storage was at −80°C. All in vitro studies were done such that the final DMSO concentration was <0.5%. *P. falciparum* ABS parasites were cultured at 3% hematocrit in human O+ RBCs in RPMI 1640 medium, supplemented with 25 mM Hepes, 50 mg l-hypoxanthine, 2 mM l-glutamine, 0.225% sodium bicarbonate, 0.5% (w/v) AlbuMAX II (Invitrogen) and gentamycin (10 μg/ml), in modular incubator chambers (Billups-Rothenberg) at 5% O_2_, 5% CO_2_, and 90% N_2_ at 37°C. Dd2-B2 (obtained from T. Wellems, National Institute of Allergy and Infectious Diseases, National Institutes of Health) is a genetically homogenous line that was cloned from Dd2 by limiting dilution. To define the EC_50_ and EC_90_ values of ABS parasites, Dd2-B2 ring-stage cultures at 0.2% parasitemia and 1% hematocrit were exposed for 72 hours to a range of 10 drug concentrations that were twofold serially diluted in duplicates along with drug-free controls. Parasite survival was assessed by flow cytometry on an iQue flow cytometer (Sartorius) using SYBR Green and MitoTracker Deep Red FM (Life Technologies) as nuclear stain and vital dyes, respectively. After determination of the EC_50_ and EC_90_ values for the three compounds with the *P. falciparum* Dd2-B2 clone, one single-step selection was set up using 2 × 10^5^ parasites in each well of a 96-well plate at a starting concentration of 3× EC_90_. Parasites were cleared from the culture rapidly, and cultures were screened three times weekly by flow cytometry and counted as positive for recrudescence when the overall parasitemia reached 0.3% and the parasites were visible on a blood smear. DNA was extracted from recrudescent lines using a QIAGEN QIAamp Blood Mini Kit, and libraries were prepared with the Illumina DNA Prep kit for whole-genome sequencing on the Illumina MiSeq using a V3 2 × 300–base pair flow cell.

### Generation and analysis of CRISPR-Cas9–based mutant lines

To test whether the PKAc (PF3D7_0934800) P305S mutation was driving resistance to SAL-0010355 (5-aryl) and SAL-0010419 (2-alkyl), CRISPR-Cas9 was used to introduce this mutation into the Dd2 parental line (*65*) to create the Dd2^PKAc_P305S^ parasite line. The parental Dd2, expressing the endogenous allele, was used as a control line. Similarly, the contribution of the PDK1 (PF3D7_1121900) N324I mutation in driving resistance to SAL-0010003 (5-benzyl) was tested by creating the Dd2^PDK1_N324^ parasite line. The Dd2 parental line, as well as two clones for each of the Dd2^PKAc_P305S^ and Dd2^PDK1_N324^ parasite lines, was tested for susceptibility to the three compounds to determine whether they conferred resistance and/or cross-resistance. Both Dd2^PKAc_P305S^ and Dd2^PDK1_N324I^ parasites were also profiled against a second independent series of predicted PDE inhibitors (NPD3518 and NPD3738) to assess for cross-resistance. The converse set of experiments was also carried out, whereby parasites containing mutations in PKAc, PDK1, and PKAr (PF3D7_1223100) selected using NPD3515 and NPD3738, were profiled for cross-resistance against the three original PDE inhibitors (SAL-0010355, SAL-0010419, and SAL-0010003).

## References

[1] “World malaria report 2023” (Geneva License: CC BY-NC-SA 3.0 IGO., 2023).

[2] B. Baragana, I. Hallyburton, M. C. Lee, N. R. Norcross, R. Grimaldi, T. D. Otto, W. R. Proto, A. M. Blagborough, S. Meister, G. Wirjanata, A. Ruecker, L. M. Upton, T. S. Abraham, M. J. Almeida, A. Pradhan, A. Porzelle, T. Luksch, M. S. Martinez, T. Luksch, J. M. Bolscher, A. Woodland, S. Norval, F. Zuccotto, J. Thomas, F. Simeons, L. Stojanovski, M. Osuna-Cabello, P. M. Brock, T. S. Churcher, K. A. Sala, S. E. Zakutansky, M. B. Jimenez-Diaz, L. M. Sanz, J. Riley, R. Basak, M. Campbell, V. M. Avery, R. W. Sauerwein, K. J. Dechering, R. Noviyanti, B. Campo, J. A. Frearson, I. Angulo-Barturen, S. Ferrer-Bazaga, F. J. Gamo, P. G. Wyatt, D. Leroy, P. Siegl, M. J. Delves, D. E. Kyle, S. Wittlin, J. Marfurt, R. N. Price, R. E. Sinden, E. A. Winzeler, S. A. Charman, L. Bebrevska, D. W. Gray, S. Campbell, A. H. Fairlamb, P. A. Willis, J. C. Rayner, D. A. Fidock, K. D. Read, I. H. Gilbert, A novel multiple-stage antimalarial agent that inhibits protein synthesis. Nature 522, 315–320 (2015). 26085270 10.1038/nature14451 PMC4700930

[3] C. W. McNamara, M. C. Lee, C. S. Lim, S. H. Lim, J. Roland, O. Simon, B. K. Yeung, A. K. Chatterjee, S. L. McCormack, M. J. Manary, A. M. Zeeman, K. J. Dechering, T. S. Kumar, P. P. Henrich, K. Gagaring, M. Ibanez, N. Kato, K. L. Kuhen, C. Fischli, A. Nagle, M. Rottmann, D. M. Plouffe, B. Bursulaya, S. Meister, L. Rameh, J. Trappe, D. Haasen, M. Timmerman, R. W. Sauerwein, R. Suwanarusk, B. Russell, L. Renia, F. Nosten, D. C. Tully, C. H. Kocken, R. J. Glynne, C. Bodenreider, D. A. Fidock, T. T. Diagana, E. A. Winzeler, Targeting *Plasmodium* PI(4)K to eliminate malaria. Nature 504, 248–253 (2013). 24284631 10.1038/nature12782 PMC3940870

[4] M. Rottmann, C. McNamara, B. K. Yeung, M. C. Lee, B. Zou, B. Russell, P. Seitz, D. M. Plouffe, N. V. Dharia, J. Tan, S. B. Cohen, K. R. Spencer, G. E. Gonzalez-Paez, S. B. Lakshminarayana, A. Goh, R. Suwanarusk, T. Jegla, E. K. Schmitt, H. P. Beck, R. Brun, F. Nosten, L. Renia, V. Dartois, T. H. Keller, D. A. Fidock, E. A. Winzeler, T. T. Diagana, Spiroindolones, a potent compound class for the treatment of malaria. Science 329, 1175–1180 (2010). 20813948 10.1126/science.1193225 PMC3050001

[5] D. A. Baker, L. B. Stewart, J. M. Large, P. W. Bowyer, K. H. Ansell, M. B. Jimenez-Diaz, M. El Bakkouri, K. Birchall, K. J. Dechering, N. S. Bouloc, P. J. Coombs, D. Whalley, D. J. Harding, E. Smiljanic-Hurley, M. C. Wheldon, E. M. Walker, J. T. Dessens, M. J. Lafuente, L. M. Sanz, F. J. Gamo, S. B. Ferrer, R. Hui, T. Bousema, I. Angulo-Barturen, A. T. Merritt, S. L. Croft, W. E. Gutteridge, C. A. Kettleborough, S. A. Osborne, A potent series targeting the malarial cGMP-dependent protein kinase clears infection and blocks transmission. Nat. Commun. 8, 430 (2017). 28874661 10.1038/s41467-017-00572-x PMC5585294

[6] P. Favuzza, M. de Lera Ruiz, J. K. Thompson, T. Triglia, A. Ngo, R. W. J. Steel, M. Vavrek, J. Christensen, J. Healer, C. Boyce, Z. Guo, M. Hu, T. Khan, N. Murgolo, L. Zhao, J. S. Penington, K. Reaksudsan, K. Jarman, M. H. Dietrich, L. Richardson, K. Y. Guo, S. Lopaticki, W. H. Tham, M. Rottmann, T. Papenfuss, J. A. Robbins, J. A. Boddey, B. E. Sleebs, H. J. Sabroux, J. A. McCauley, D. B. Olsen, A. F. Cowman, Dual plasmepsin-targeting antimalarial agents disrupt multiple stages of the malaria parasite life cycle. Cell Host Microbe. 27, 642–658 e12 (2020). 32109369 10.1016/j.chom.2020.02.005 PMC7146544

[7] D. A. Baker, L. G. Drought, C. Flueck, S. D. Nofal, A. Patel, M. Penzo, E. M. Walker, Cyclic nucleotide signalling in malaria parasites. Open Biol. 7, 331–339 (2017). 10.1098/rsob.170213 PMC5746546 29263246

[8] A. J. Perrin, A. Patel, C. Flueck, M. J. Blackman, D. A. Baker, cAMP signalling and its role in host cell invasion by malaria parasites. Curr. Opin. Microbiol. 58, 69–74 (2020). 33032143 10.1016/j.mib.2020.09.003

[9] A. C. Balestra, K. Koussis, N. Klages, S. A. Howell, H. R. Flynn, M. Bantscheff, C. Pasquarello, A. J. Perrin, L. Brusini, P. Arboit, O. Sanz, L. P. Castano, C. Withers-Martinez, A. Hainard, S. Ghidelli-Disse, A. P. Snijders, D. A. Baker, M. J. Blackman, M. Brochet, Ca^2+^ signals critical for egress and gametogenesis in malaria parasites depend on a multipass membrane protein that interacts with PKG. Sci. Adv. 7, eabe5396 (2021). 33762339 10.1126/sciadv.abe5396 PMC7990342

[10] C. R. Collins, F. Hackett, M. Strath, M. Penzo, C. Withers-Martinez, D. A. Baker, M. J. Blackman, Malaria parasite cGMP-dependent protein kinase regulates blood stage merozoite secretory organelle discharge and egress. PLoS Pathog. 9, e1003344 (2013). 23675297 10.1371/journal.ppat.1003344 PMC3649973

[11] K. Koussis, C. Withers-Martinez, D. A. Baker, M. J. Blackman, Simultaneous multiple allelic replacement in the malaria parasite enables dissection of PKG function. Life Sci. Alliance 3, e201900626 (2020). 32179592 10.26508/lsa.201900626 PMC7081069

[12] H. M. Taylor, L. McRobert, M. Grainger, A. Sicard, A. R. Dluzewski, C. S. Hopp, A. A. Holder, D. A. Baker, The malaria parasite cyclic GMP-dependent protein kinase plays a central role in blood-stage schizogony. Eukaryot. Cell 9, 37–45 (2010). 19915077 10.1128/EC.00186-09 PMC2805293

[13] A. Dawn, S. Singh, K. R. More, F. A. Siddiqui, N. Pachikara, G. Ramdani, G. Langsley, C. E. Chitnis, The central role of cAMP in regulating *Plasmodium falciparum* merozoite invasion of human erythrocytes. PLoS Pathog. 10, e1004520 (2014). 25522250 10.1371/journal.ppat.1004520 PMC4270784

[14] E. Hitz, N. Wiedemar, A. Passecker, B. A. S. Graca, C. Scheurer, S. Wittlin, N. M. B. Brancucci, I. Vakonakis, P. Maser, T. S. Voss, The 3-phosphoinositide-dependent protein kinase 1 is an essential upstream activator of protein kinase A in malaria parasites. PLoS Biol. 19, e3001483 (2021). 34879056 10.1371/journal.pbio.3001483 PMC8687544

[15] A. Patel, A. J. Perrin, H. R. Flynn, C. Bisson, C. Withers-Martinez, M. Treeck, C. Flueck, G. Nicastro, S. R. Martin, A. Ramos, T. W. Gilberger, A. P. Snijders, M. J. Blackman, D. A. Baker, Cyclic AMP signalling controls key components of malaria parasite host cell invasion machinery. PLoS Biol. 17, e3000264 (2019). 31075098 10.1371/journal.pbio.3000264 PMC6530879

[16] M. L. Wilde, T. Triglia, D. Marapana, J. K. Thompson, A. A. Kouzmitchev, H. E. Bullen, P. R. Gilson, A. F. Cowman, C. J. Tonkin, Protein kinase A is essential for invasion of *Plasmodium falciparum* into human erythrocytes. mBio 10, e01972-19 (2019). 31594816 10.1128/mBio.01972-19 PMC6786871

[17] A. Samidurai, L. Xi, A. Das, R. C. Kukreja, Beyond erectile dysfunction: cGMP-specific phosphodiesterase 5 inhibitors for other clinical disorders. Annu. Rev. Pharmacol. Toxicol. 63, 585–615 (2023). 36206989 10.1146/annurev-pharmtox-040122-034745

[18] C. Flueck, L. G. Drought, A. Jones, A. Patel, A. J. Perrin, E. M. Walker, S. D. Nofal, A. P. Snijders, M. J. Blackman, D. A. Baker, Phosphodiesterase beta is the master regulator of cAMP signalling during malaria parasite invasion. PLoS Biol. 17, e3000154 (2019). 30794532 10.1371/journal.pbio.3000154 PMC6402698

[19] C. J. Taylor, L. McRobert, D. A. Baker, Disruption of a *Plasmodium falciparum* cyclic nucleotide phosphodiesterase gene causes aberrant gametogenesis. Mol. Microbiol. 69, 110–118 (2008). 18452584 10.1111/j.1365-2958.2008.06267.x PMC2615252

[20] L. Wentzinger, S. Bopp, H. Tenor, J. Klar, R. Brun, H. P. Beck, T. Seebeck, Cyclic nucleotide-specific phosphodiesterases of *Plasmodium falciparum*: PfPDEα, a non-essential cGMP-specific PDE that is an integral membrane protein. Int. J. Parasitol. 38, 1625–1637 (2008). 18590734 10.1016/j.ijpara.2008.05.016

[21] K. Yuasa, F. Mi-Ichi, T. Kobayashi, M. Yamanouchi, J. Kotera, K. Kita, K. Omori, PfPDE1, a novel cGMP-specific phosphodiesterase from the human malaria parasite *Plasmodium falciparum*. Biochem. J. 392, 221–229 (2005). 16038615 10.1042/BJ20050425 PMC1317681

[22] R. M. Kuehnel, E. Ganga, A. C. Balestra, C. Suarez, M. Wyss, N. Klages, L. Brusini, B. Maco, N. Brancucci, T. S. Voss, D. Soldati, M. Brochet, A *Plasmodium* membrane receptor platform integrates cues for egress and invasion in blood forms and activation of transmission stages. Sci. Adv. 9, eadf2161 (2023). 37327340 10.1126/sciadv.adf2161 PMC10275601

[23] V. Lakshmanan, M. E. Fishbaugher, B. Morrison, M. Baldwin, M. Macarulay, A. M. Vaughan, S. A. Mikolajczak, S. H. Kappe, Cyclic GMP balance is critical for malaria parasite transmission from the mosquito to the mammalian host. mBio 6, e02330 (2015). 25784701 10.1128/mBio.02330-14 PMC4453516

[24] R. W. Moon, C. J. Taylor, C. Bex, R. Schepers, D. Goulding, C. J. Janse, A. P. Waters, D. A. Baker, O. Billker, A cyclic GMP signalling module that regulates gliding motility in a malaria parasite. PLoS Pathog. 5, e1000599 (2009). 19779564 10.1371/journal.ppat.1000599 PMC2742896

[25] M. E. N’Dri, T. A. Tavella, L. Royer, F. Dupuy, L. Bedault, F. Verdier, C. Lavazec, Phosphodiesterase delta governs the mechanical properties of erythrocytes infected with *Plasmodium falciparum* gametocytes. Microbes. Infect. 25, 105102 (2023). 36708871 10.1016/j.micinf.2023.105102

[26] A. Nzila, L. Mwai, In vitro selection of *Plasmodium falciparum* drug-resistant parasite lines. J. Antimicrob. Chemother. 65, 390–398 (2010). 20022938 10.1093/jac/dkp449 PMC2818104

[27] P. K. Rathod, T. McErlean, P. C. Lee, Variations in frequencies of drug resistance in *Plasmodium falciparum*. Proc. Natl. Acad. Sci. U.S.A. 94, 9389–9393 (1997). 9256492 10.1073/pnas.94.17.9389 PMC23200

[28] L. McRobert, C. J. Taylor, W. Deng, Q. L. Fivelman, R. M. Cummings, S. D. Polley, O. Billker, D. A. Baker, Gametogenesis in malaria parasites is mediated by the cGMP-dependent protein kinase. PLoS Biol. 6, e139 (2008). 18532880 10.1371/journal.pbio.0060139 PMC2408617

[29] W. J. Stone, T. S. Churcher, W. Graumans, G. J. van Gemert, M. W. Vos, K. H. Lanke, M. G. van de Vegte-Bolmer, R. Siebelink-Stoter, K. J. Dechering, A. M. Vaughan, N. Camargo, S. H. Kappe, R. W. Sauerwein, T. Bousema, A scalable assessment of *Plasmodium falciparum* transmission in the standard membrane-feeding assay, using transgenic parasites expressing green fluorescent protein-luciferase. J. Infect. Dis. 210, 1456–1463 (2014). 24829466 10.1093/infdis/jiu271

[30] G. Chen, H. Wang, H. Robinson, J. Cai, Y. Wan, H. Ke, An insight into the pharmacophores of phosphodiesterase-5 inhibitors from synthetic and crystal structural studies. Biochem. Pharmacol. 75, 1717–1728 (2008). 18346713 10.1016/j.bcp.2008.01.019 PMC2409583

[31] Y. Zheng, A. Matheeussen, L. Maes, G. Caljon, G. J. Sterk, R. Leurs, Structural optimization of BIPPO analogs as potent antimalarials. Molecules 28, 4939 (2023). 37446602 10.3390/molecules28134939 PMC10343887

[32] C. Lugnier, The complexity and multiplicity of the specific cAMP phosphodiesterase family: PDE4, open new adapted therapeutic approaches. Int. J. Mol. Sci. 23, 10616 (2022). 36142518 10.3390/ijms231810616 PMC9502408

[33] A. Hatzelmann, E. J. Morcillo, G. Lungarella, S. Adnot, S. Sanjar, R. Beume, C. Schudt, H. Tenor, The preclinical pharmacology of roflumilast—A selective, oral phosphodiesterase 4 inhibitor in development for chronic obstructive pulmonary disease. Pulm. Pharmacol. Ther. 23, 235–256 (2010). 20381629 10.1016/j.pupt.2010.03.011

[34] L. Fala, Otezla (apremilast), an oral PDE-4 inhibitor, receives FDA approval for the treatment of patients with active psoriatic arthritis and plaque psoriasis. Am. Health Drug Benefits 8, 105–110 (2015). 26629274 PMC4665061

[35] M. G. Matera, J. Ora, F. Cavalli, P. Rogliani, M. Cazzola, New avenues for phosphodiesterase inhibitors in asthma. J. Exp. Pharmacol. 13, 291–302 (2021). 33758554 10.2147/JEP.S242961 PMC7979323

[36] J. Prickaerts, P. R. A. Heckman, A. Blokland, Investigational phosphodiesterase inhibitors in phase 1 and phase 2 clinical trials for Alzheimer’s disease. Expert Opin. Investig. Drugs 26, 1033–1048 (2017). 10.1080/13543784.2017.1364360 28772081

[37] G. Ramdani, B. Naissant, E. Thompson, F. Breil, A. Lorthiois, F. Dupuy, R. Cummings, Y. Duffier, Y. Corbett, O. Mercereau-Puijalon, K. Vernick, D. Taramelli, D. A. Baker, G. Langsley, C. Lavazec, cAMP-signalling regulates gametocyte-infected erythrocyte deformability required for malaria parasite transmission. PLoS Pathog. 11, e1004815 (2015). 25951195 10.1371/journal.ppat.1004815 PMC4423841

[38] G. Bouyer, D. Barbieri, F. Dupuy, A. Marteau, A. Sissoko, M. E. N’Dri, G. Neveu, L. Bedault, N. Khodabux, D. Roman, S. Houze, G. Siciliano, P. Alano, R. M. Martins, J. J. Lopez-Rubio, J. Clain, R. Duval, S. Egee, C. Lavazec, *Plasmodium falciparum* sexual parasites regulate infected erythrocyte permeability. Commun. Biol. 3, 726 (2020). 33262483 10.1038/s42003-020-01454-7 PMC7708629

[39] B. L. Howard, K. L. Harvey, R. J. Stewart, M. F. Azevedo, B. S. Crabb, I. G. Jennings, P. R. Sanders, D. T. Manallack, P. E. Thompson, C. J. Tonkin, P. R. Gilson, Identification of potent phosphodiesterase inhibitors that demonstrate cyclic nucleotide-dependent functions in apicomplexan parasites. ACS Chem. Biol. 10, 1145–1154 (2015). 25555060 10.1021/cb501004q

[40] B. L. Howard, P. E. Thompson, D. T. Manallack, Active site similarity between human and *Plasmodium falciparum* phosphodiesterases: Considerations for antimalarial drug design. J. Comput. Aided. Mol. Des. 25, 753–762 (2011). 21766240 10.1007/s10822-011-9458-5

[41] T. B. Beghyn, J. Charton, F. Leroux, G. Laconde, A. Bourin, P. Cos, L. Maes, B. Deprez, Drug to genome to drug: Discovery of new antiplasmodial compounds. J. Med. Chem. 54, 3222–3240 (2011). 21504142 10.1021/jm1014617

[42] T. B. Beghyn, J. Charton, F. Leroux, A. Henninot, I. Reboule, P. Cos, L. Maes, B. Deprez, Drug-to-genome-to-drug, step 2: Reversing selectivity in a series of antiplasmodial compounds. J. Med. Chem. 55, 1274–1286 (2012). 22204607 10.1021/jm201422e

[43] A. E. Leroux, J. O. Schulze, R. M. Biondi, AGC kinases, mechanisms of regulation and innovative drug development. Semin. Cancer Biol. 48, 1–17 (2018). 28591657 10.1016/j.semcancer.2017.05.011

[44] M. Vanaerschot, J. M. Murithi, C. F. A. Pasaje, S. Ghidelli-Disse, L. Dwomoh, M. Bird, N. Spottiswoode, N. Mittal, L. B. Arendse, E. S. Owen, K. J. Wicht, G. Siciliano, M. Bosche, T. Yeo, T. R. S. Kumar, S. Mok, E. F. Carpenter, M. J. Giddins, O. Sanz, S. Ottilie, P. Alano, K. Chibale, M. Llinas, A. C. Uhlemann, M. Delves, A. B. Tobin, C. Doerig, E. A. Winzeler, M. C. S. Lee, J. C. Niles, D. A. Fidock, Inhibition of resistance-refractory *P. falciparum* kinase PKG delivers prophylactic, blood stage, and transmission-blocking antiplasmodial activity. Cell Chem. Biol. 27, 806–816 e808 (2020). 32359426 10.1016/j.chembiol.2020.04.001 PMC7369637

[45] B. J. Morahan, C. Abrie, K. Al-Hasani, M. B. Batty, V. Corey, A. N. Cowell, J. Niemand, E. A. Winzeler, L. M. Birkholtz, C. Doerig, J. F. Garcia-Bustos, Human Aurora kinase inhibitor Hesperadin reveals epistatic interaction between *Plasmodium falciparum* PfArk1 and PfNek1 kinases. Commun. Biol. 3, 701 (2020). 33219324 10.1038/s42003-020-01424-z PMC7679417

[46] P. Pantziarka, V. Sukhatme, S. Crispino, G. Bouche, L. Meheus, V. P. Sukhatme, Repurposing drugs in oncology (ReDO)-selective PDE5 inhibitors as anti-cancer agents. Ecancermedicalscience 12, 824 (2018). 29743944 10.3332/ecancer.2018.824 PMC5931815

[47] W. Trager, J. B. Jensen, Human malaria parasites in continuous culture. Science 193, 673–675 (1976). 781840 10.1126/science.781840

[48] F. J. Gamo, L. M. Sanz, J. Vidal, C. de Cozar, E. Alvarez, J. L. Lavandera, D. E. Vanderwall, D. V. Green, V. Kumar, S. Hasan, J. R. Brown, C. E. Peishoff, L. R. Cardon, J. F. Garcia-Bustos, Thousands of chemical starting points for antimalarial lead identification. Nature 465, 305–310 (2010). 20485427 10.1038/nature09107

[49] M. Filarsky, S. A. Fraschka, I. Niederwieser, N. M. B. Brancucci, E. Carrington, E. Carrio, S. Moes, P. Jenoe, R. Bartfai, T. S. Voss, GDV1 induces sexual commitment of malaria parasites by antagonizing HP1-dependent gene silencing. Science 359, 1259–1263 (2018). 29590075 10.1126/science.aan6042 PMC6219702

[50] L. M. Sanz, B. Crespo, C. De-Cozar, X. C. Ding, J. L. Llergo, J. N. Burrows, J. F. Garcia-Bustos, F. J. Gamo, *P. falciparum* in vitro killing rates allow to discriminate between different antimalarial mode-of-action. PLOS ONE 7, e30949 (2012). 22383983 10.1371/journal.pone.0030949 PMC3285618

[51] N. J. White, Assessment of the pharmacodynamic properties of antimalarial drugs in vivo. Antimicrob. Agents Chemother. 41, 1413–1422 (1997). 9210658 10.1128/aac.41.7.1413 PMC163932

[52] A. K. Ghosh, R. R. Dinglasan, H. Ikadai, M. Jacobs-Lorena, An improved method for the in vitro differentiation of *Plasmodium falciparum* gametocytes into ookinetes. Malar. J. 9, 194 (2010). 20615232 10.1186/1475-2875-9-194 PMC2909250

[53] A. Ruecker, D. K. Mathias, U. Straschil, T. S. Churcher, R. R. Dinglasan, D. Leroy, R. E. Sinden, M. J. Delves, A male and female gametocyte functional viability assay to identify biologically relevant malaria transmission-blocking drugs. Antimicrob. Agents Chemother. 58, 7292–7302 (2014). 25267664 10.1128/AAC.03666-14 PMC4249523

[54] S. D’Alessandro, Y. Corbett, D. P. Ilboudo, P. Misiano, N. Dahiya, S. M. Abay, A. Habluetzel, R. Grande, M. R. Gismondo, K. J. Dechering, K. M. Koolen, R. W. Sauerwein, D. Taramelli, N. Basilico, S. Parapini, Salinomycin and other ionophores as a new class of antimalarial drugs with transmission-blocking activity. Antimicrob. Agents Chemother. 59, 5135–5144 (2015). 26055362 10.1128/AAC.04332-14 PMC4538477

[55] S. D’Alessandro, F. Silvestrini, K. Dechering, Y. Corbett, S. Parapini, M. Timmerman, L. Galastri, N. Basilico, R. Sauerwein, P. Alano, D. Taramelli, A *Plasmodium falciparum* screening assay for anti-gametocyte drugs based on parasite lactate dehydrogenase detection. J. Antimicrob. Chemother. 68, 2048–2058 (2013). 23645588 10.1093/jac/dkt165

[56] M. T. Makler, D. J. Hinrichs, Measurement of the lactate dehydrogenase activity of *Plasmodium falciparum* as an assessment of parasitemia. Am. J. Trop. Med. Hyg. 48, 205–210 (1993). 8447524 10.4269/ajtmh.1993.48.205

[57] M. J. Delves, U. Straschil, A. Ruecker, C. Miguel-Blanco, S. Marques, A. C. Dufour, J. Baum, R. E. Sinden, Routine in vitro culture of *P. falciparum* gametocytes to evaluate novel transmission-blocking interventions. Nat. Protoc. 11, 1668–1680 (2016). 27560172 10.1038/nprot.2016.096

[58] M. Kristan, S. G. Thorburn, J. C. Hafalla, C. J. Sutherland, M. C. Oguike, Mosquito and human hepatocyte infections with *Plasmodium ovale curtisi* and *Plasmodium ovale wallikeri*. Trans. R Soc. Trop. Med. Hyg. 113, 617–622 (2019). 31162595 10.1093/trstmh/trz048

[59] K. Witmer, E. Sherrard-Smith, U. Straschil, M. Tunnicliff, J. Baum, M. Delves, An inexpensive open source 3D-printed membrane feeder for human malaria transmission studies. Malar. J. 17, 282 (2018). 30075783 10.1186/s12936-018-2436-9 PMC6076392

[60] M. W. Vos, W. J. Stone, K. M. Koolen, G. J. van Gemert, B. van Schaijk, D. Leroy, R. W. Sauerwein, T. Bousema, K. J. Dechering, A semi-automated luminescence based standard membrane feeding assay identifies novel small molecules that inhibit transmission of malaria parasites by mosquitoes. Sci. Rep. 5, 18704 (2015). 26687564 10.1038/srep18704 PMC4685452

[61] A. M. Feldmann, T. Ponnudurai, Selection of *Anopheles stephensi* for refractoriness and susceptibility to *Plasmodium falciparum*. Med. Vet. Entomol. 3, 41–52 (1989). 2519646 10.1111/j.1365-2915.1989.tb00473.x

[62] M. Smilkstein, N. Sriwilaijaroen, J. X. Kelly, P. Wilairat, M. Riscoe, Simple and inexpensive fluorescence-based technique for high-throughput antimalarial drug screening. Antimicrob. Agents Chemother. 48, 1803–1806 (2004). 15105138 10.1128/AAC.48.5.1803-1806.2004 PMC400546

[63] K. Sriprawat, S. Kaewpongsri, R. Suwanarusk, M. L. Leimanis, U. Lek-Uthai, A. P. Phyo, G. Snounou, B. Russell, L. Renia, F. Nosten, Effective and cheap removal of leukocytes and platelets from *Plasmodium vivax* infected blood. Malar. J. 8, 115 (2009). 19490618 10.1186/1475-2875-8-115 PMC2694833

[64] K. H. Rieckmann, F. J. Lopez Antunano, Chloroquine resistance of *Plasmodium falciparum* in Brazil detected by a simple in vitro method. Bull. World Health Organ. 45, 157–167 (1971). 4945631 PMC2427915

[65] J. M. Murithi, C. Pascal, J. Bath, X. Boulenc, N. F. Gnadig, C. F. A. Pasaje, K. Rubiano, T. Yeo, S. Mok, S. Klieber, P. Desert, M. B. Jimenez-Diaz, J. Marfurt, M. Rouillier, M. H. Cherkaoui-Rbati, N. Gobeau, S. Wittlin, A. C. Uhlemann, R. N. Price, G. Wirjanata, R. Noviyanti, P. Tumwebaze, R. A. Cooper, P. J. Rosenthal, L. M. Sanz, F. J. Gamo, J. Joseph, S. Singh, S. Bashyam, J. M. Augereau, E. Giraud, T. Bozec, T. Vermat, G. Tuffal, J. M. Guillon, J. Menegotto, L. Salle, G. Louit, M. J. Cabanis, M. F. Nicolas, M. Doubovetzky, R. Merino, N. Bessila, I. Angulo-Barturen, D. Baud, L. Bebrevska, F. Escudie, J. C. Niles, B. Blasco, S. Campbell, G. Courtemanche, L. Fraisse, A. Pellet, D. A. Fidock, D. Leroy, The antimalarial MMV688533 provides potential for single-dose cures with a high barrier to *Plasmodium falciparum* parasite resistance. Sci. Transl. Med. 13, eabg6013 (2021). 34290058 10.1126/scitranslmed.abg6013 PMC8530196

[66] K. Y. Zhang, G. L. Card, Y. Suzuki, D. R. Artis, D. Fong, S. Gillette, D. Hsieh, J. Neiman, B. L. West, C. Zhang, M. V. Milburn, S. H. Kim, J. Schlessinger, G. Bollag, A glutamine switch mechanism for nucleotide selectivity by phosphodiesterases. Mol. Cell 15, 279–286 (2004). 15260978 10.1016/j.molcel.2004.07.005

[67] L. A. Kelley, M. J. Sternberg, Protein structure prediction on the web: A case study using the Phyre server. Nat. Protoc. 4, 363–371 (2009). 19247286 10.1038/nprot.2009.2

