# Supplementary material for: Inhibitors of malaria parasite cyclic nucleotide phosphodiesterases block asexual blood-stage development and mosquito transmission
Source: Sci Adv. 2024 Dec 6;10(49):eadq1383. doi: 10.1126/sciadv.adq1383 (PMC11623267; doi:10.1126/sciadv.adq1383)
Supplement: Supplementary file 1 — Figs. S1 to S13 Tables S1 to S6 Data S1 [file sciadv.adq1383_sm.pdf]

Science Advances
AAAS

Supplementary Materials for

Inhibitors of malaria parasite cyclic nucleotide phosphodiesterases block asexual blood-stage development and mosquito transmission

Paula-Josefina Gomez-Gonzalez *et al.*

Corresponding author: David A. Baker, david.baker@lshtm.ac.uk; Mark Gardner, mark.gardner@salvensis.org

Sci. Adv. **10**, eadq1383 (2024)
DOI: 10.1126/sciadv.adq1383

This PDF file includes:

Figs. S1 to S13
Tables S1 to S6
Data S1

A

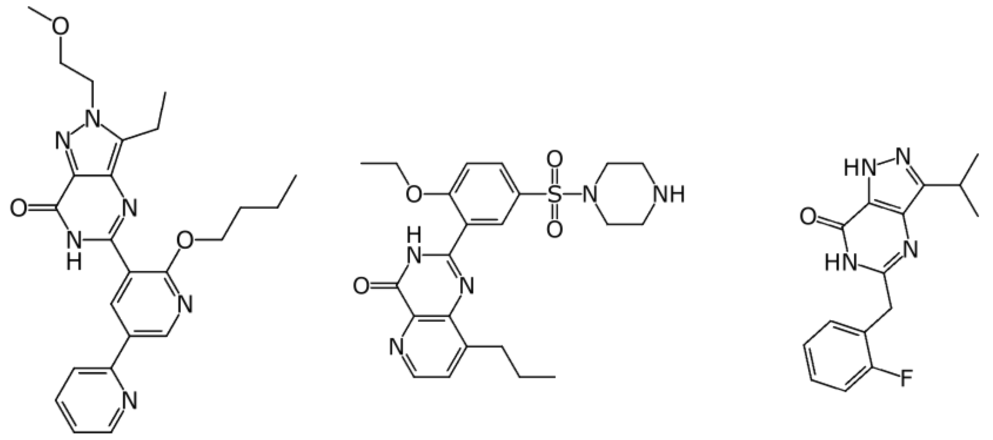

SAL-0010034 (2-alkyl) SAL-0010043 (5-aryl) SAL-0010042 (5-benzyl)

B

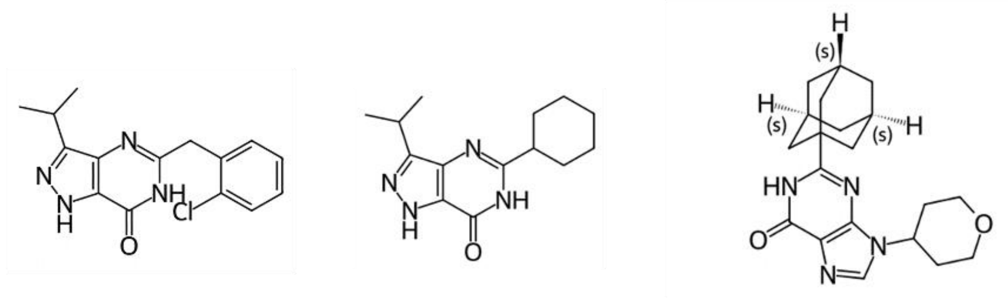

NPD-2958 NPD-3518 NPD-3738

Figure S1. Structures of chemistry start points of the three subseries and independent NPD inhibitor series. (A) Structures of the chemistry start points for each of the three PDEβ inhibitor subseries (2-alkyl, 5-aryl and 5-benzyl). **(B)** Structures of the independent NPD inhibitor series.

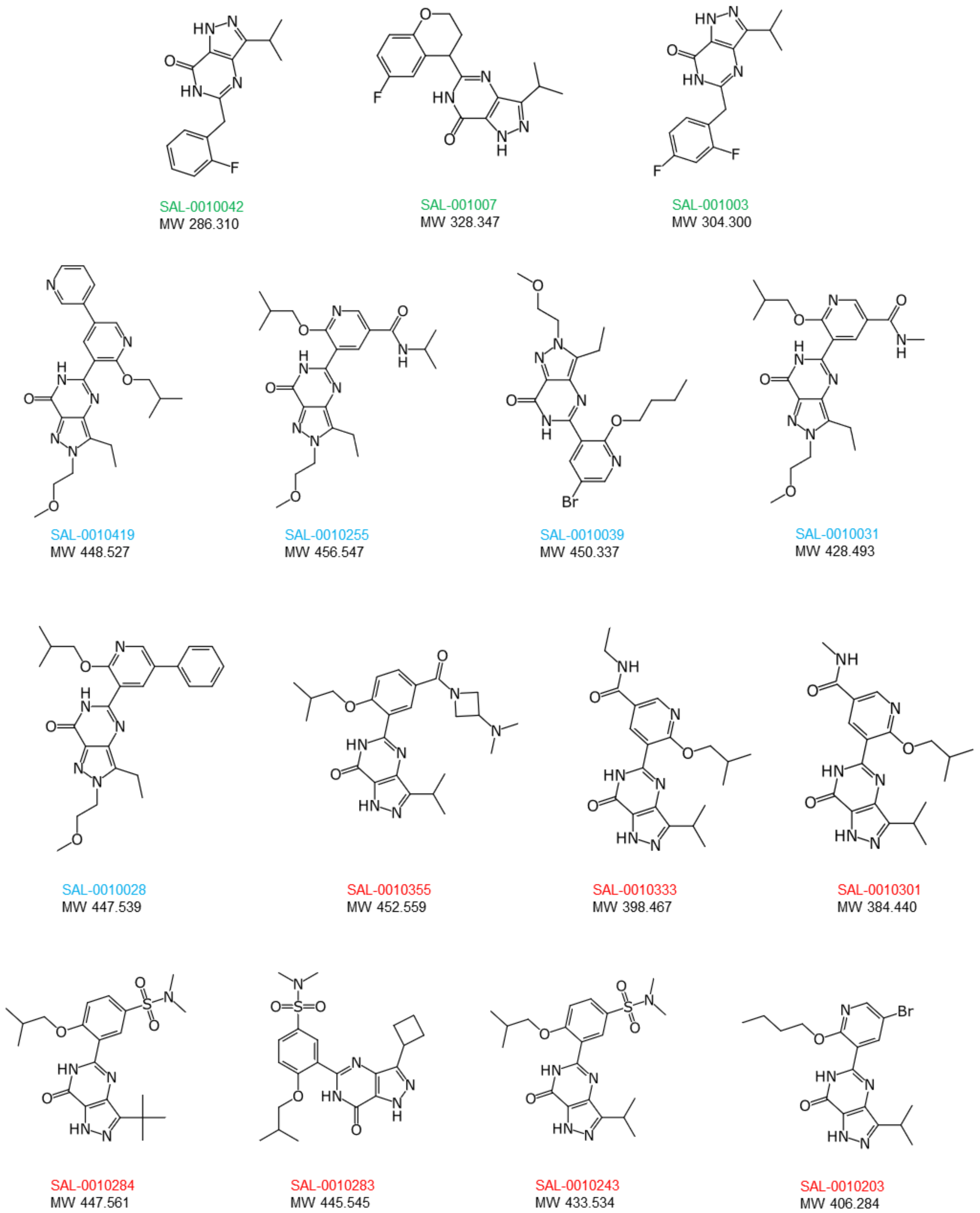

Figure S2. Chemical structures of the 15 compounds which were the main focus of this study. Chemical structures are shown for examples of the 5-benzyl (green), 2-alkyl (blue) and 5-aryl (red) that were used in one or more measurements in this study are shown with their corresponding molecular weights.

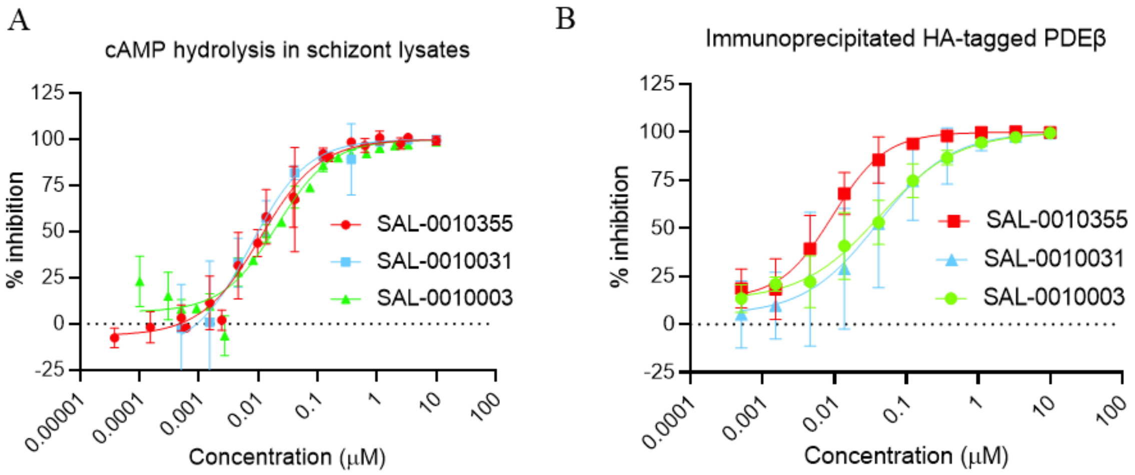

Figure S3. Measurement of cAMP hydrolysis in asexual blood stage parasites. (A) Example dose response curves showing measurement of cAMP hydrolysis in *P. falciparum* blood stage schizont lysates for each of the three chemical sub-series. Green is 5-benzyl, red is 5-aryl and blue is 2-alkyl. **(B)** Example dose response curves showing measurement of cAMP hydrolysis by immunoprecipitated HA-tagged PDEβ from schizont preparations for each of the three subseries.

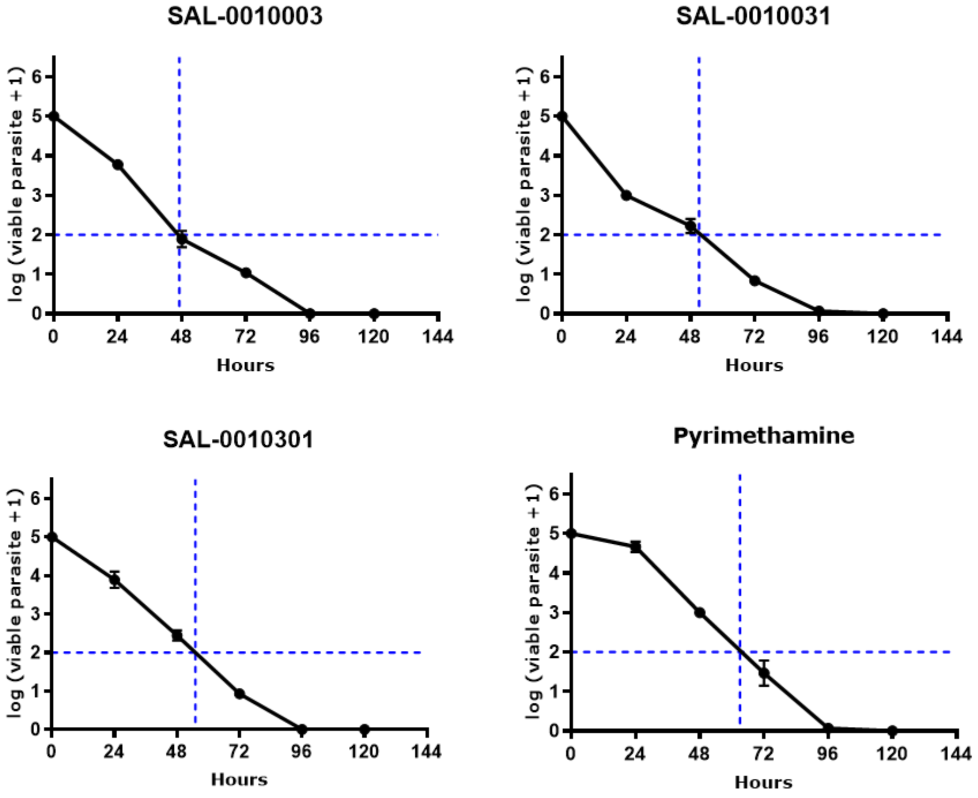

Figure S4. Parasite Reduction Ratio (PRR) *P. falciparum* blood stage killing profiles for examples of the three sub-series. PRR killing profile plots obtained by incubating parasites at a concentration of 10× EC_{50} for an example of each inhibitor sub-series: SAL-0010003 (5-benzyl), SAL-0010031 (2-alkyl) and SAL-001301 (5-aryl) and the pyrimethamine control.

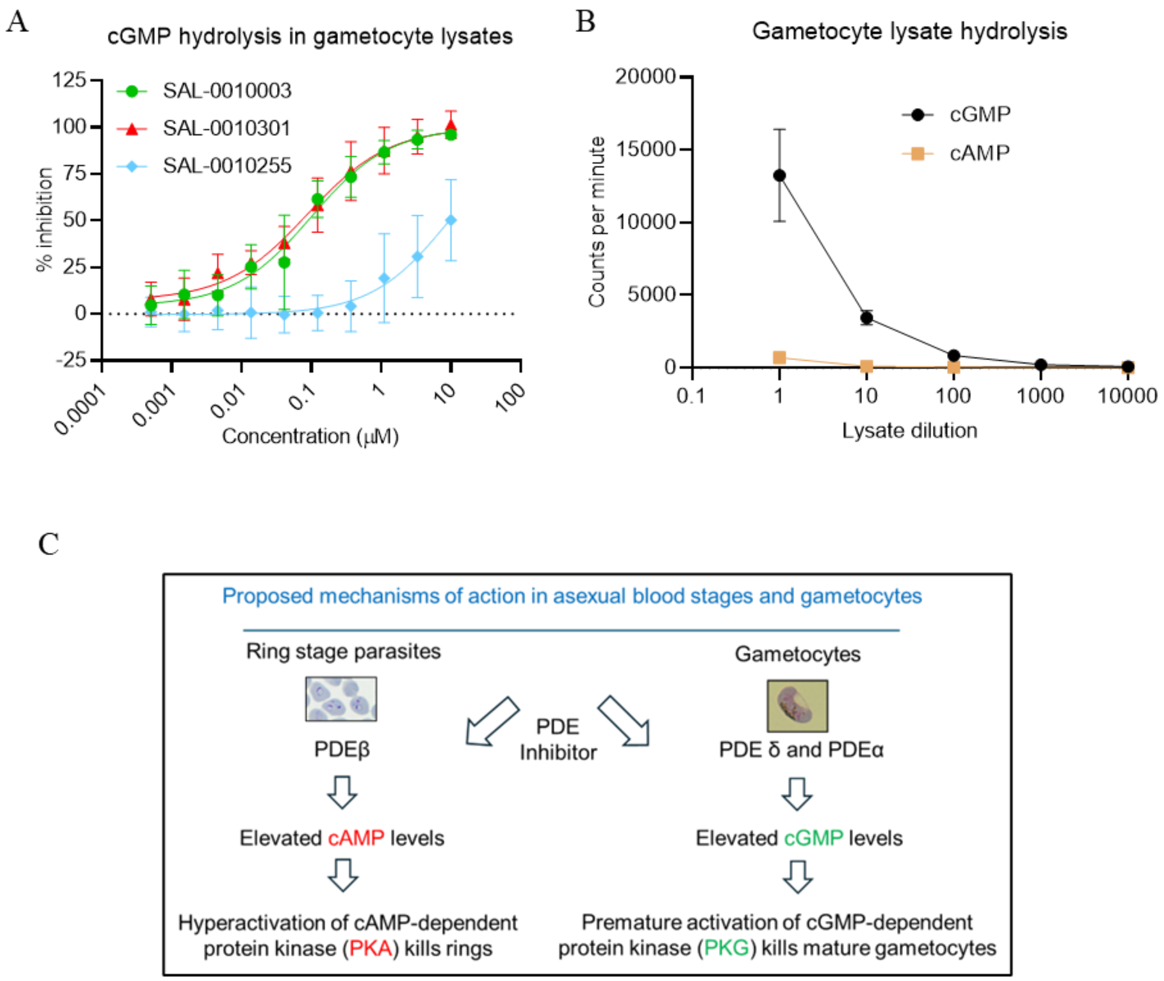

Figure S5. Activity of compounds against gametocytes and the proposed targets at this life cycle stage. (A) Examples of dose response curves for measurement of cGMP hydrolysis for each of the three chemical subseries. Green is 5-benzyl, red is 5-aryl and blue is 2-alkyl. **(B)** Measurement of cAMP and cGMP hydrolysis in stage IV/V gametocyte lysates (n=2, error bars are SD). **(C)** Schematic showing the mechanisms of action of the compounds in asexual blood stages and gametocytes and the respective targets.

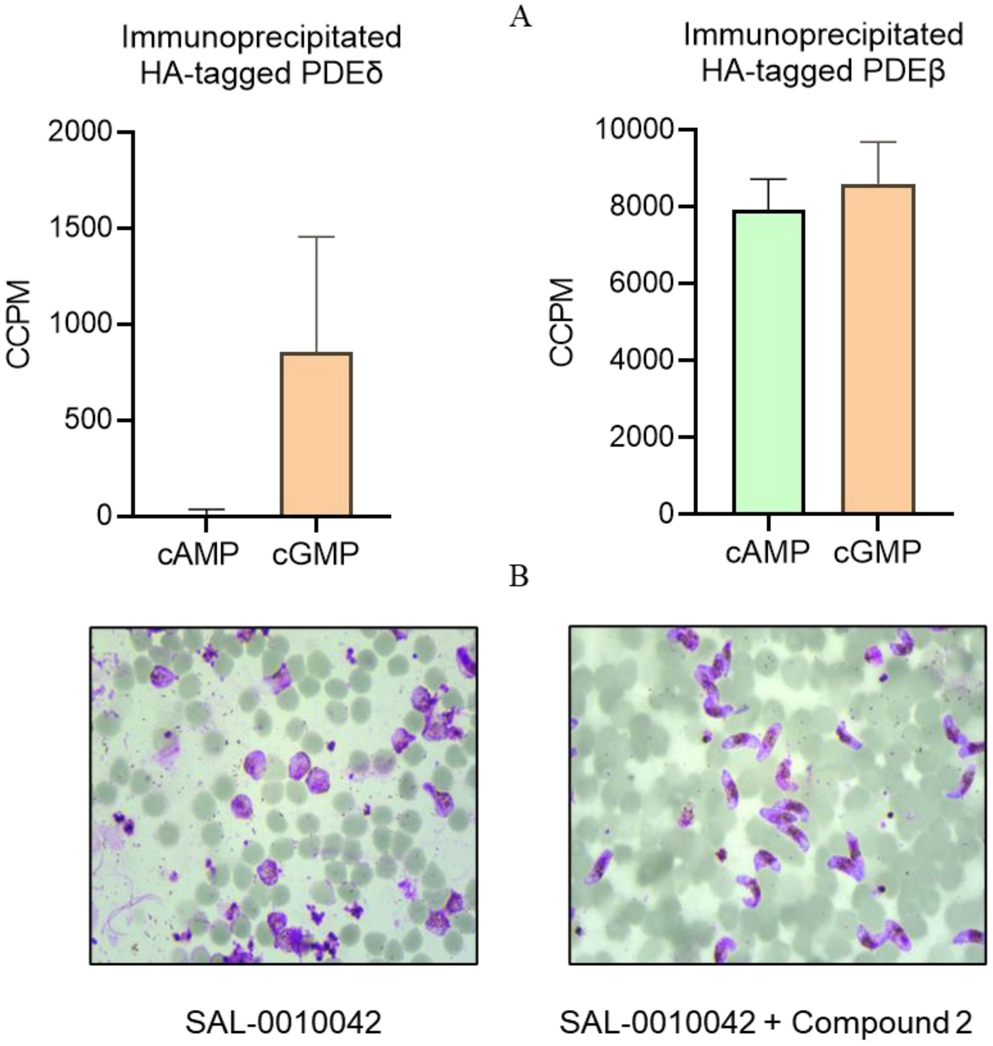

Figure S6. Substrate specificity of PDEδ and PDEβ and ablation of the effects of a compound by a PKG inhibitor. (A) Measurement of cyclic nucleotide specificity of immunoprecipitated HA-tagged PDEδ (left) and HA-tagged PDEβ (right) assayed in parallel. N=4, error bars represent SD. CCPM is corrected counts per minute. **(B)** Giemsa-stained blood films showing the effects of SAL-0010042 (5-benzyl) on mature gametocytes in the absence (left) and presence of a parasite-specific PKG inhibitor, Compound 2.

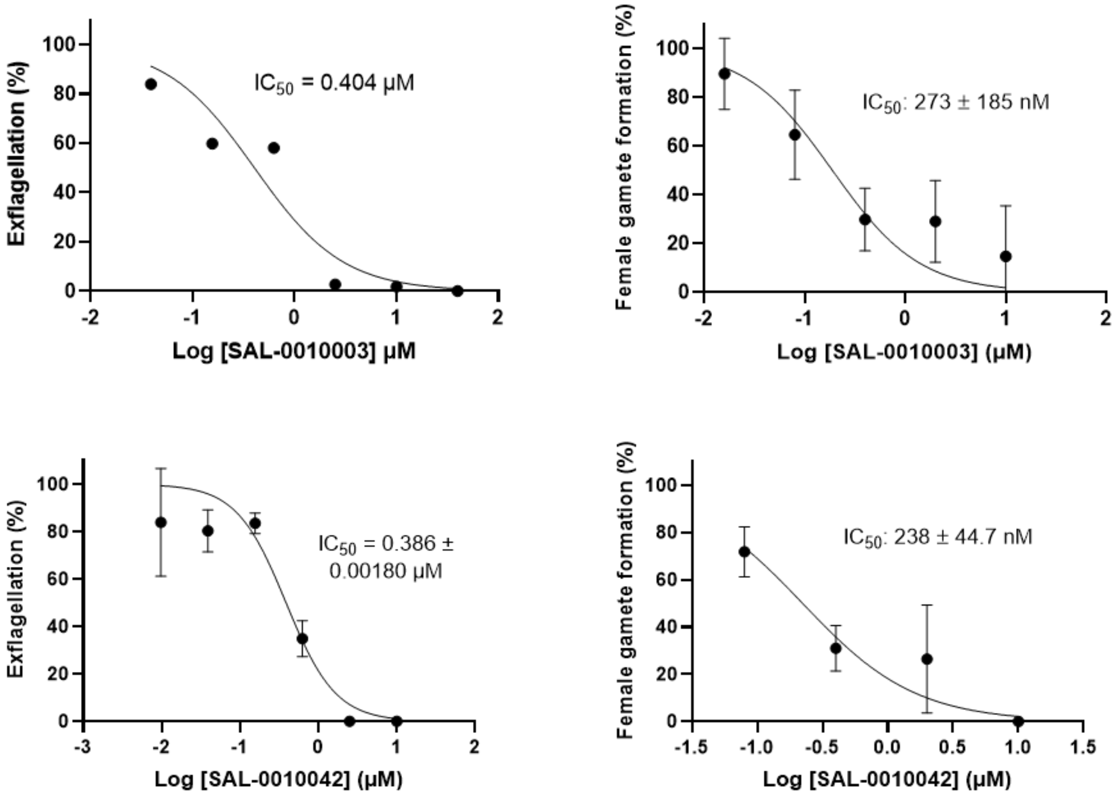

Figure S7. IC_{50} curves for exflagellation and female gamete formation. IC_{50} curves for two 5-benzyl compounds tested for inhibition of both male gamete formation (exflagellation) and female gamete formation. For experiments carried out on three independent biological replicates, error bars are SD.

A

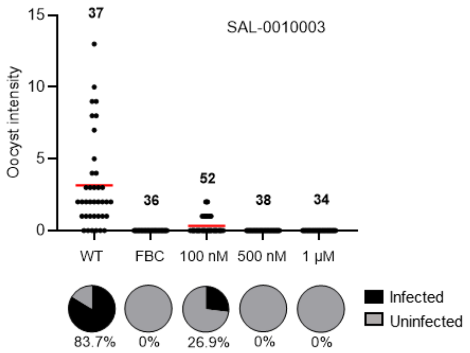

	Total Dissected	Total Infected	Oocyst Prevalence (%)	Oocyst Intensity (per midgut)	Sporozoite Prevalence (%)
WT	37	31	83.7	3.2	61.5
FBC	36	0	0	0	0
100 nM	52	14	26.9	0.3	46.2
500 nM	38	0	0	0	0
1 µM	34	0	0	0	0

B

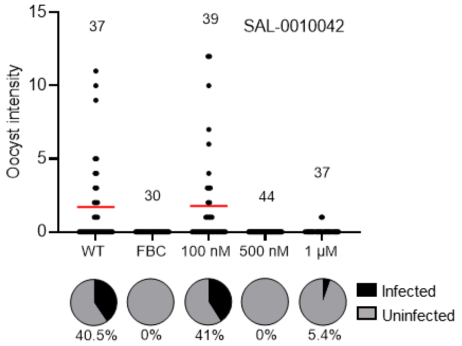

	Total Dissected	Total Infected	Oocyst Prevalence (%)	Oocyst Intensity (per midgut)	Sporozoite Prevalence (%)
WT	37	15	40.5	1.7	63.6
FBC	30	0	0	0	0
100 nM	39	16	41	1.8	50
500 nM	44	0	0	0	0
1 µM	37	2	5.4	0	0

Figure S8. Initial SMFA testing for effects on oocysts and sporozoites. The plots show the effects of increasing **(A)** SAL-0010003 and **(B)** SAL-0010042 inhibitor concentration on the infection intensity. The number of mosquitoes dissected from each feed is indicated. The mean intensity is indicated by a red bar. Methylene blue (at a concentration of 1 µM) was used as a 'full block' control, FBC. The pie charts below show the effects on infection prevalence. The tables below indicate the numbers of mosquitoes dissected and infected, as well as the infection prevalence and intensity for oocysts and the infection prevalence for sporozoites.

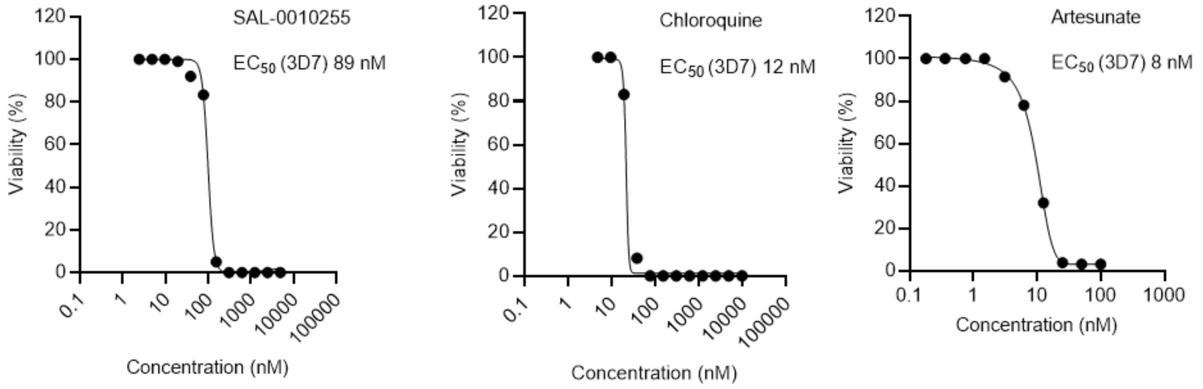

Figure S9. EC_{50} curves for compounds tested *ex vivo* against clinical isolates and lab isolates. EC_{50} curves for SAL-0010255 (2-alkyl) and two control antimalarials using *P. falciparum* clone 3D7 tested in parallel with clinical isolates from Porto Velho, RO in the Brazilian Amazon.

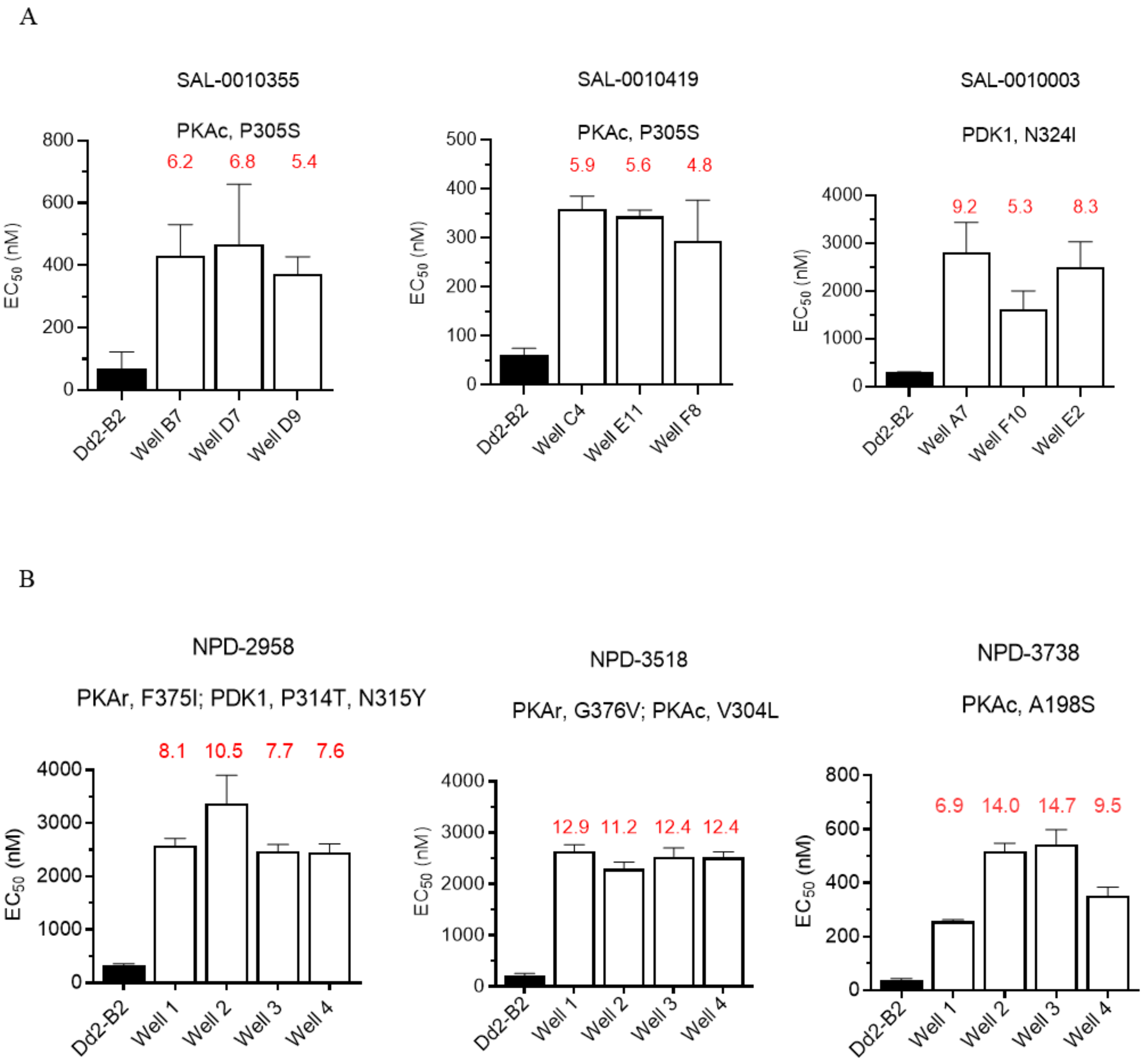

Figure S10. Changes in inhibitor susceptibility in parasites selected under drug pressure *in vitro*. (A) The changes in EC_{50} values of parasites selected with an example of the three PDEβ inhibitor subseries: SAL-0010355 (5-aryl), SAL-0010419 (2-alkyl) and SAL-0010003 (5-benzyl). The fold change in EC_{50} value compared to the parental Dd2B2 line is shown in red, with the mutations indicated above **(B)** The changes in EC_{50} values of parasites selected with three examples of the independent PDEβ inhibitor NPD series. The fold change in EC_{50} value, compared to the Dd2B2 parental line is shown in red, and the mutations selected are indicated above.

PKAc

MQFIKNLQLNKKKDSDSSEQVLTNKKNKMKYEDFNFIRTLGTGSFGRVILATYKNGNYPPVAIKRFEKCKIIRQKQVDHV

FSERKILNYINHPFCVNLHGSFKDDSYLYLVLEFVIGGEFFTFLRRNKRFPNDVGCFYAAQIVLIFEYLQSLNIVYRDLK

PENLLLDKDGFIKMTDFGFAKIVETRTYTLCGTPEYIAPEILLNVGHGKAADWWTLGIFIYEILVGCPPFYANEPLLIYQ

KILEGIIYFPKFLDNNCKHLMKKLLSHDLTKRYGNLKKGAQNVKEHPWFSNIDWVNLLNKNVEVPYKPKYKNIFDSSNFE

RVQEDLTIADKITNENDPFYDW

PDK1

MKKGFLLNKNIYDIEENVINVEKNNTNKKYCKDDFEIYMHIGTGNFSDVFMVKLKNDPSKKYALKIFKKEKVNKMNKVND

VLTEKNVMSKLNTPGHANVIKLIETFKDKENVYLLYEYADYDLWEFLKIRSVGVNEKITFNIILQMVHALIYIHNKNIIH

RDLKCENFLINKDGTIKMIDFGSSKDLDNISIKTNNEEDTINHEELSKFVLKKNNNNNSNEDLKNANEFKNNDSLNGEEI

NNNDLNNTNFQKSDKNIKDQNSNKCVLETLKNNESYEFNNQILSSFKSNDYAADANKHNSYKKKKTFENYVGSPNFIPPE

ALINKCSGKARDFWSLGCTIYQLVTCTVPFDGSTEWFIYNKIKRRELKYPSIIPSELIDLIEKLTTMNPEERLGFNGGCE

EILEHLYFQKYNYNKLNFILPEVSELEKLYTTIINKYHIYINEKRKLRQNNNSTEENINNVEVLKKNLLNLINSDTLVCA

EEEYESITLKKKIFKSINFMLEEFDKQEIKEMEEASKWLERYQGT

PKAr

MGNVCTWRQGKEKAGDDNSQVIKDKELQNEFKTFEQKMRSNKKNAHEGDMNNDGEDDRYKFSRGFSLSKKPSKTKIPITK

TDSEILDGLDYSEMSKQVLMTLNKKNILNDDGSSDGNDTDVHSMFDRKEIERKVLDLESIHFIQKKRLSVSAEAYGDWNK

KIDNFIPKVYKKDEKEKAKIREALNESFLFNHLNKKEFEIIVNAFFDKNVEKGVNIINEGDYGDLLYVIDQGEVEIYKTK

ENNKKEVLTVLKSKDVFGELALLYNSKRAATATALTKCHLWALDRESFTYIIKDMVAKKRKMYEDILSHVNILKDMDPYE

RCKVADCLKSKSYNDGEIIIKEGEEGDTFFILIDGNAVASKDNKVIKTYTKGDYFGELALLKNKPRAATIKAQNFCQVVY

LDRKSFKRLLGPIEDILHRNVENYKKVLNELGLDTTCIDEN

Figure S11. Amino acid sequences of proteins in which mutations were selected under drug pressure. Top panel, amino acid sequence of the catalytic domain of the *P. falciparum* cAMP-dependent protein kinase (PKAc; PF3D7_0934800). The positions of the amino acids that are changed by mutations selected under drug pressure are coloured red. Key sequence motifs adjacent to the selected sequence changes are boxed and highly-conserved amino acids within the motifs are in bold. Middle panel, amino acid sequence of the *P. falciparum* 3-phosphoinositide-dependent protein kinase (PDK1; PF3D7_1121900). The positions of the amino acids that are changed by mutations selected under drug pressure are coloured red. Key sequence motifs adjacent to the selected sequence changes are boxed and highly conserved amino acids within the motifs are in bold. The hatched box indicates a malaria parasite-specific sequence insert that interrupts the kinase domain. Bottom panel, amino acid sequence of the regulatory domain of the *P. falciparum* cAMP-dependent protein kinase (PKAc; PF3D7_0934800). The positions of the amino acids that are changed by mutations selected under drug pressure are coloured red. The predicted cAMP-binding domains are boxed, and highly-conserved amino acids are in bold.

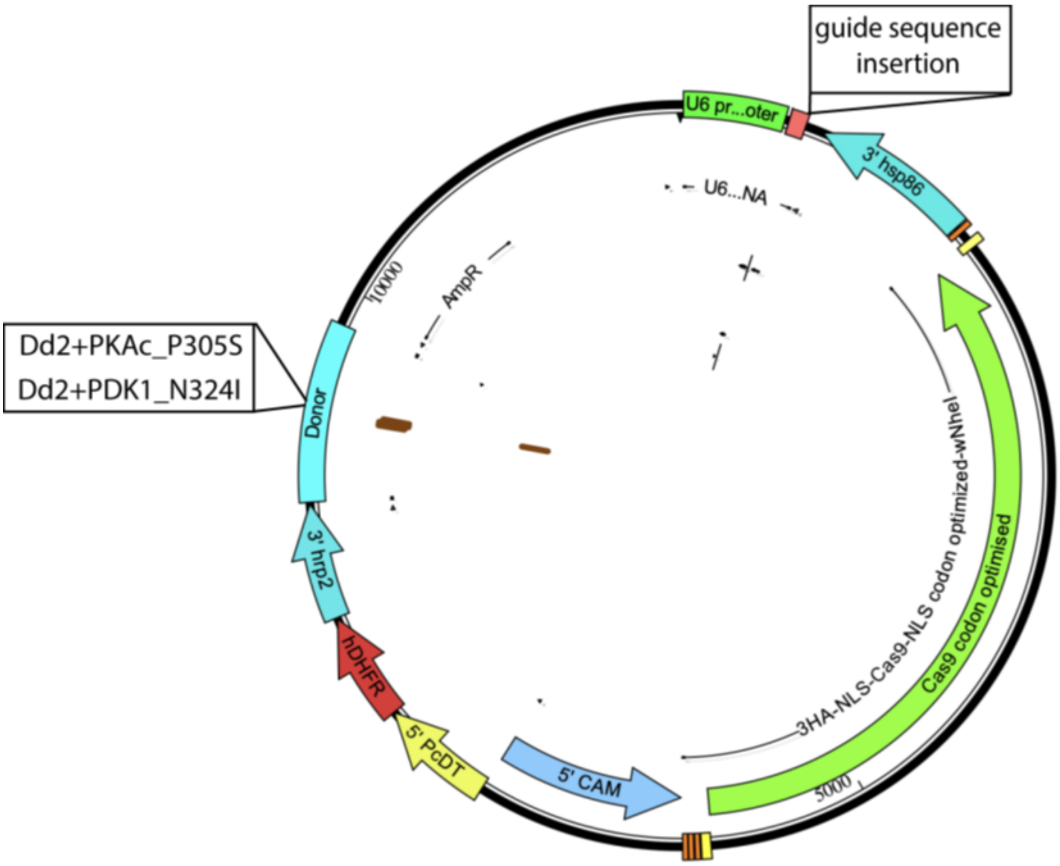

Figure S12. Plasmid map of constructs used to introduce in vitro-selected mutations into parental parasites by CRISPR-based gene editing. All-in-one plasmid map of the constructs used to introduce the PKAc and PDK1 mutations selected *in vitro* using the 5-aryl, 2-alkyl and 5-benyl compounds respectively into the Dd2-B2 parental line. The plasmid contains the donor region, an hDHFR selection cassette, CRISPR/Cas9 enzyme and guide in a single plasmid.

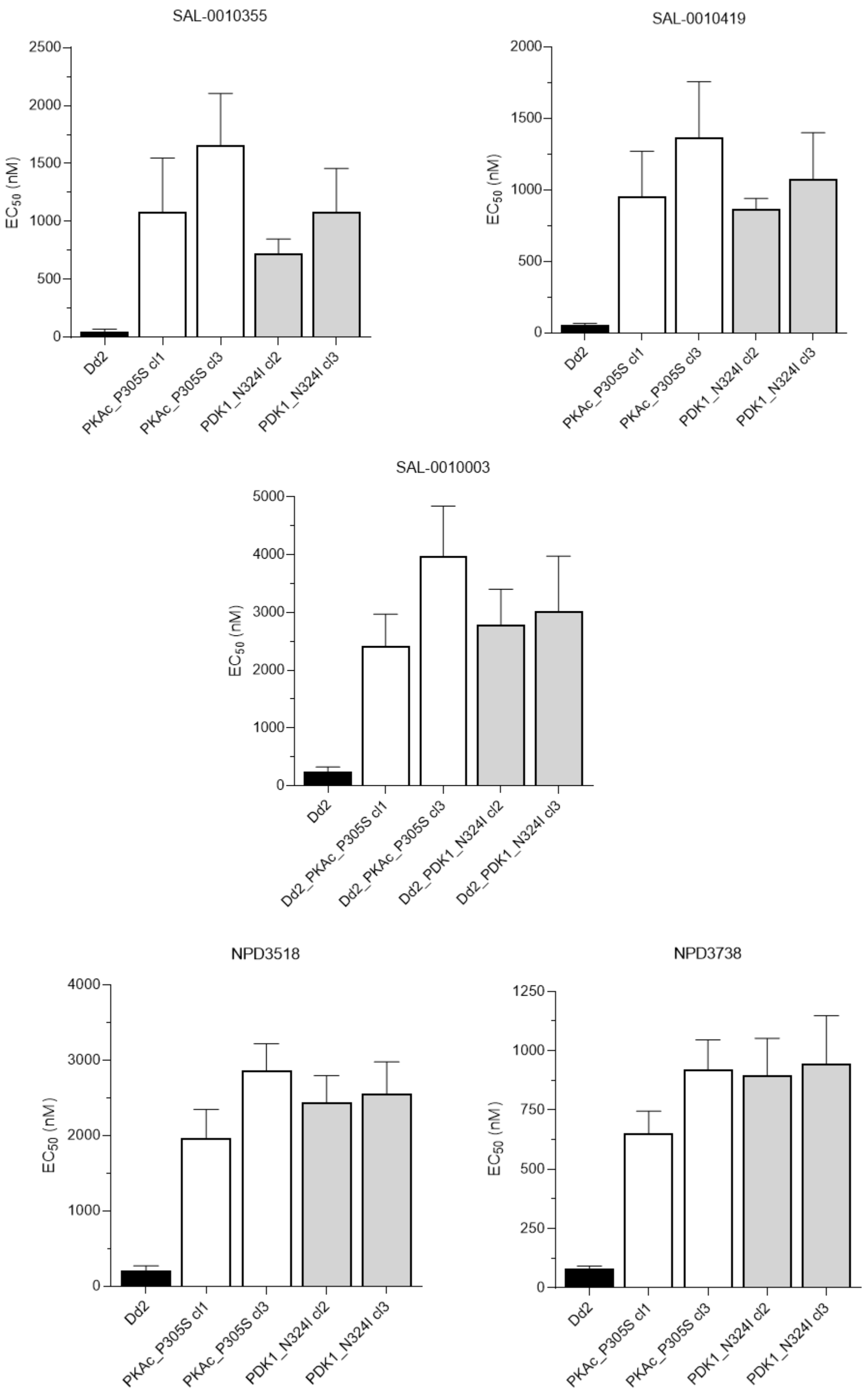

Figure S13A. Changes in inhibitor susceptibility in gene-edited parasites. Changes in EC_{50} values of gene-edited parasites (two independent clones of each) harbouring the PKAc and PDK1 mutants selected with SAL-0010355, SAL-0010419 and SAL-0010003 (see **Table 4**) respectively compared to the Dd2B2 parental line, when tested against an example of the three PDEβ inhibitor subseries and two examples of the independent PDEβ inhibitor NPD series.

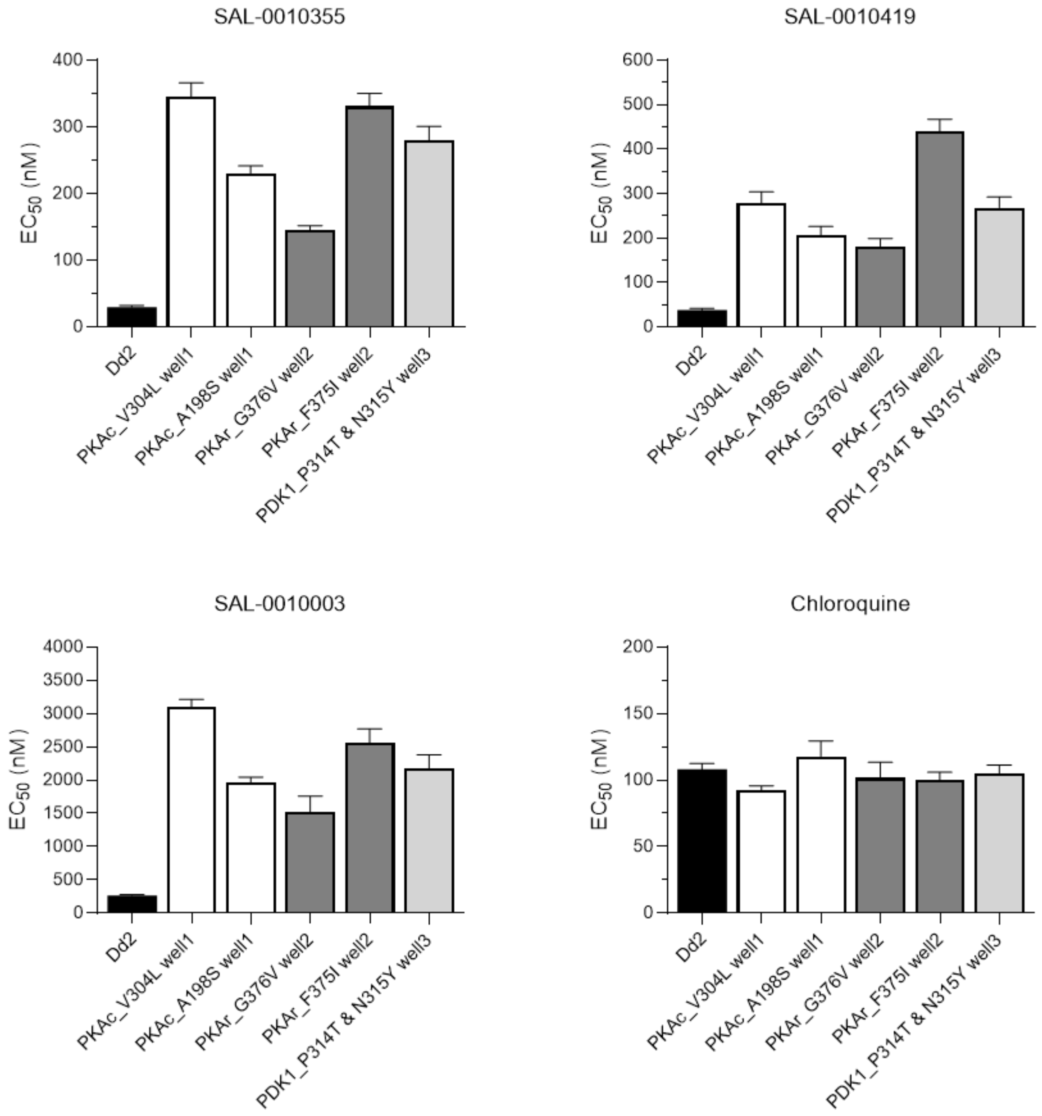

Figure S13B. Changes in inhibitor susceptibility in gene-edited parasites. The changes in EC_{50} values of gene-edited parasites harbouring the PKAc, PKAr and PDK1 mutants selected with the NPD series (see **Table 4**) compared to the Dd2B2 parental line, when tested against an example of the three PDEβ inhibitor subseries and a chloroquine control.

Compound	*P. falciparum* 3D7	*P. falciparum* clinical isolates	
	EC_{50} (nM)	No (% of total)	Median EC_{50} (range)
SAL-0010255	89	8 (100)	157 (39-321)
Artesunate	8	8 (100)	0.6 (0.1-9)
Chloroquine	12	8 (100)	1202 (626-2595)

Table S1A. Activity against Brazilian clinical isolates. Median EC_{50} values obtained with *P. falciparum* clinical isolates from Porto Velho, Brazil and for the *P. falciparum* 3D7 line for SAL-0010255 (2-alkyl) and control antimalarials.

	***P. falciparum* EC$_{50}$ values (nM)**						
	**Lab strains**		**Clinical isolates**				
**Compound**	**3D7 (N^a)**	**Dd2 (N^a)**	**N^a**	**Median**	**Gmean**	**Range**	**H.S. (N)**
SAL-0010042	132 (4)	115 (4)	47	197	204	94-499	2.93 (45)
SAL-0010333	223 (4)	181 (4)	47	160	167	25-1072	2.98 (44)
Pyrimethamine	46 (4)	21678 (4)	47	39300	41591	15870-196000	2.02 (48)
Chloroquine	9.4 (4)	347 (4)	47	14	13	2.0-25	4.51 (35)

Table S1B. Activity against Ugandan clinical isolates. EC$_{50}$ values obtained with clinical isolates from the Tororo district in Uganda (taken between October 2020 and January 2021) and for the 3D7 and Dd2 lines for SAL-0010042 (5-benyl), SAL-0010333 (5-aryl) and control antimalarials. N^a, total number of isolates that have acceptable EC$_{50}$ values in the Z factor and curve-fitting criteria. HS is the mean Hill slope derived from the curve fits (N is the number of isolates for which non-constrained slopes were derived). Gmean is the geometric mean.

	Dd2-B2	SAL-0010355 Well B7	SAL-0010355 Well D7	SAL-0010355 Well D9
EC50 (nM)	69.2	430.3	468.5	371.4
EC_{50} shift (fold)		6.2	6.8	5.4
N	7	4	4	3

	Dd2-B2	SAL-0010419 Well C4	SAL-0010419 Well E11	SAL-0010419 Well F8
EC_{50} (nM)	61.4	359.5	344.0	293.1
EC_{50} shift (fold)		5.9	5.6	4.8
N	7	6	5	2

	Dd2-B2	SAL-0010003 Well A7	SAL-0010003 Well F10	SAL-0010003 Well E2
EC_{50} (nM)	303.9	2800	1619.4	2507.3
EC_{50} shift (fold)		9.2	5.3	8.3
N	3	3	3	3

Table S2A. EC_{50} fold changes in parasites recovered under drug pressure. Fold change in EC_{50} values in mutant parasites selected in vitro under drug pressure with SAL-0010355 (5-aryl), SAL-0010419 (2-alkyl) and SAL-0010003 (5-benzyl), compared to the Dd2-B2 parental line.

	**Dd2-B2**	**NPD-2958 Well 1**	**NPD-2958 Well 2**	**NPD-2958 Well 3**	**NPD-2958 Well 4**
EC_{50} (nM)	319.7	2574	3369	2471	2445
EC_{50} shift (fold)		8.1	10.5	7.7	7.6
N	4	4	4	4	4

	**Dd2-B2**	**NPD-3518 Well 1**	**NPD-3518 Well 2**	**NPD-3518 Well 3**	**NPD-3518 Well 4**
EC_{50} (nM)	203.4	2628	2290	2530	2514
EC_{50} shift (fold)		12.9	11.2	12.4	12.4
N	4	4	4	4	4

	**Dd2-B2**	**NPD-3738 Well 1**	**NPD-3738 Well 2**	**NPD-3738 Well 3**	**NPD-3738 Well 4**
EC_{50} (nM)	37.12	257.1	518.0	544.5	352.9
EC_{50} shift (fold)		6.9	14.0	14.7	9.5
N	4	4	4	4	4

Table S2B. EC_{50} fold changes in parasites recovered under drug pressure. Fold change in EC_{50} values in mutant parasites selected in vitro under drug pressure with three examples for the independently developed PDEβ inhibitor series.

Whole-genome sequencing metrics of samples sequenced on Illumina MiSeq

Sample names		**B7**	**D7**	**D9**
		**Samples**		
Total reads		5,230,593	5,030,493	4,669,799
# Mapped reads		4,859,426	4,669,452	4,319,918
Duplication rate		37.90%	39.25%	37.19%
General error rate		1.75%	1.75%	1.79%
Mean mapping quality (Phred)		56.58	56.66	56.56
Depth of coverage	***mean***	45.89	43.42	40.90
	SD	45.97	37.74	36.64
% of Pf genome with > x no. reads	***1X***	96.17%	96.18%	96.18%
	5X	94.61%	94.60%	94.57%
	10X	93.21%	93.08%	92.99%
	30X	79.41%	77.44%	74.86%

The only homozygous SNP found in PF3D7_0934800 in the three samples which were compared to a Dd2-B2 reference

**CHROM**	**POS**	**REF**	**ALT**	**AMINO ACID CHANGE**	**CODON CHANGE**	**GENE NAME**	**EFFECT / IMPACT**
Pf3D7_09_v3	1363732	C	T	P305S	Cca/Tca	PF3D7_0934800 (cAMP-dependent protein kinase catalytic subunit)	NON SYN CODING / MODERATE

All samples carrying the SNP are shown. Note: 100% represents an alternate allelic balance containing all alternate reads (homozygous alternate) and 0% contains all reference reads (homozygous reference). Anything in between is considered heterozygous.

**GENE NAME**	**AMINO ACID CHANGE**	**CODON CHANGE**	**Resistant clones**		
			**B7**	**D7**	**D9**
Pf3D7_0934800	P305S	Cca/Tca	100%	100%	100%

Table S3A. Summary of the whole-genome sequencing data for cloned parasites selected with SAL-0010355 (5-aryl).

Whole-genome sequencing metrics of samples sequenced on Illumina MiSeq

Sample names		Samples C4	E11
Total reads		4,568,325	5,337,321
# Mapped reads		4,226,051	4,911,172
Duplication rate		37.16%	39.20%
General error rate		1.80%	1.76%
Mean mapping quality (Phred)		56.6	56.57
Depth of coverage	***mean***	39.69	46.66
	SD	36.23	46.24
% of Pf genome with > x no. reads	***1X***	96.14%	96.24%
	5X	94.46%	94.73%
	10X	92.66%	93.35%
	30X	72.67%	79.78%

The only homozygous SNP found in PF3D7_0934800 in the two samples compared to a Dd2-B2 reference

CHROM	POS	REF	ALT	AMINO ACID CHANGE	CODON CHANGE	GENE NAME	EFFECT / IMPACT
Pf3D7_09_v3	1363732	C	T	P305S	Cca/Tca	PF3D7_0934800 (cAMP-dependent protein kinase catalytic subunit)	NON SYN CODING / MODERATE

All samples carrying the SNP are shown. Note: 100% represents an alternate allelic balance containing all alternate reads (homozygous alternate) and 0% contains all reference reads (homozygous reference). Anything in between is considered heterozygous.

GENE NAME	AMINO ACID CHANGE	CODON CHANGE	Resistant clones C4	E11
Pf3D7_0934800	P305S	Cca/Tca	100%	100%

Table S3B. Summary of the whole-genome sequencing data for cloned parasites selected with SAL-0010419 (2-alkyl).

Whole-genome sequencing metrics of samples sequenced on Illumina MiSeq

		Samples			Parent
Sample names		**A7**	**E2**	**F10**	**Dd2-B2**
Total reads		3,437,516	3,764,291	4,412,133	3,318,534
# Mapped reads		2,161,672	3,281,484	3,443,246	2,974,443
Duplication rate		38.98%	36.01%	28.51%	40.81%
General error rate		1.69%	1.65%	1.71%	2.12%
Mean mapping quality (Phred)		56.36	56.79	56.42	56.51
Depth of coverage	***mean***	17.21	29.74	31.39	29.13
	SD	22.24	34.81	49.83	43.85
% of Pf genome with > x no. reads	***1X***	95.34%	95.72%	95.98%	95.68%
	5X	90.39%	93.46%	93.95%	93.72%
	10X	78.16%	89.57%	91.23%	90.56%
	30X	7.52%	50.20%	54.48%	48.80%

This is the only SNP found in PF3D7_1121900

CHROM	POS	REF	ALT	AMINO ACID CHANGE	CODON CHANGE	GENE NAME	EFFECT / IMPACT
Pf3D7_11_v3	836496	T	A	N324I	aAt/aTt	PF3D7_1121900 (serine/threonine protein kinase, putative)	NON SYN CODING / MODERATE

Each of the lines had one homozygous N324I SNP in a serine/threonine protein kinase (PF3D7_1121900). Note: 100% represents an alternate allelic balance containing all alternate reads (homozygous alternate) and 0% contains all reference reads (homozygous reference). Anything in between are considered heterozygous.

GENE NAME	AMINO ACID CHANGE	CODON CHANGE	Resistant clones		
			**A7**	**E2**	**F10**
Pf3D7_1121900	N324I	aAt/aTt	100%	100%	100%

Table S3C. Summary of the whole-genome sequencing data for cloned parasites selected with SAL-0010003 (5-benzyl).

Sample names		**Resistant clones**					
		**NPD2958 Well2**	**NPD2958 Well3**	**NPD3518 Well1**	**NPD3518 Well2**	**NPD3738 Well1**	**NPD3738 Well3**
Total reads		4,249,911	4,463,073	5,110,205	4,888,743	4,914,798	5,168,119
# Mapped reads		3,926,145	4,115,654	4,730,128	4,544,168	4,471,324	4,735,371
Duplication rate		26.86%	27.11%	29.01%	28.04%	27.68%	28.84%
General error rate		1.32%	1.33%	1.27%	1.33%	1.31%	1.30%
Mean mapping quality (Phred)		56.41	56.38	56.4	56.3	56.36	56.45
Depth of coverage	***mean***	**22.41**	**23.55**	**27.21**	**26.13**	**25.58**	**26.91**
	SD	16.76	15.47	16.61	15.16	18.25	18.00
% of PF genome with > x no. reads	***1X***	95.51%	95.64%	95.73%	95.77%	95.67%	95.58%
	5X	93.25%	93.55%	93.80%	93.72%	93.62%	93.60%
	10X	**90.57%**	**91.43%**	**92.07%**	**91.91%**	**91.91%**	**91.98%**
	30X	17.93%	22.25%	41.07%	35.59%	31.79%	39.19%

Table S4. Summary of the whole-genome sequencing data for cloned parasites selected with the independently developed NPD compound series.

Table : List of oligonucleotides used in this study

Name	Nucleotide Sequence (5'-3')	Description
p1	CATATTAAGTATATAATATTATGGTTTAGTAATAT TGATTgtttAagagctaTGCTGgaa	*pf0934800* guide 1 forward
p2	ttcCAGCAtagctctTaaacAATCAATATTACTAAACCA TAATATTATATACTTAATATG	*pf0934800* guide 1 reverse
p3	CATATTAAGTATATAATATTTTGATTCCTCCAATT TTGAGgtttAagagctaTGCTGgaa	*pf0934800* guide 2 forward
p4	ttcCAGCAtagctctTaaacCTCAAAATTGGAGGAATCA AAATATTATATACTTAATATG	*pf0934800* guide 2 reverse
p5	CATATTAAGTATATAATATTGCATTAATAAATAAA TGCAGgtttAagagctaTGCTGgaa	*pf1121900* guide 1 forward
p6	ttcCAGCAtagctctTaaacCTGCATTTATTTATTAATG CAATATTATATACTTAATATG	*pf1121900* guide 1 reverse
p7	CATATTAAGTATATAATATTGCTTCAGGGGGAAT GAAATTgtttAagagctaTGCTGgaa	*pf1121900* guide 2 forward
p8	ttcCAGCAtagctctTaaacAATTTCATTCCCCCTGAAG CAATATTATATACTTAATATG	*pf1121900* guide 2 reverse
p9	GTAAATTTACATGGATCATTCAAAGATGAC	*pf0934800* PCR primer forward
p10	CTACCAATCATAAAATGGATCATTTTC	*pf0934800* PCR primer reverse
p11	CATATACGAAATATTAGTTGG	*pf0934800* sequencing primer
p12	ATGAAGAAAGGATTTTTGTTGAATAAG	*pf1121900* PCR primer forward
p13	CTATGTACCTTGATATCGTTCTAACC	*pf1121900* PCR primer reverse
p14	TGATAGTTTAAATGGCGAAGA	*pf1121900* sequencing primer forward
p15	TCTTCGCCATTTAAACTATCA	*pf1121900* sequencing primer reverse

Table S5. Oligonucleotide primers used to generate CRISPR-based gene edited lines harbouring the PKAc and PDK1 mutations that were selected under drug pressure with the 5-aryl, 2-alkyl and 5-benzyl subseries.

Line/well	Selection compound	Fold shift	Mutation	Locus
Dd2-B2_2958_W2	NPD-2958	10.5	F375I	PKAr
Dd2-B2_2958_W3	NPD-2958	7.7	P314T & N315Y	PDK1
Dd2-B2_3518_W2	NPD-3518	11.2	G376V	PKAr
Dd2-B2_3738_W1	NPD-3738	6.9	A198S	PKAc
Dd2-B2_0003_A7, E2, F10	SAL-0010003	9.2, 8.3, 5.3	N324I	PDK1
Dd2-B2_0355_B7, D7, D9	SAL-0010355	6.2, 6.8, 5.4	P305S	PKAc
Dd2-B2_0419_C4, E11	SAL-0010419	5.9, 5.6	P305S	PKAc

Table S6. **Summary of mutations selected, and of changes in inhibitor susceptibility of lines selected *in vitro* under drug pressure**.

Data S1

Chemistry Analytical data

LCMS Method-A

Water Acquity H Class UPLC attached with Waters SQD 2 mass spectrometer.

Ionisation method: Electro spray, Capillary (kV) 3.50, Cone (V) 25.00, Source Temperature (°C) 150, Desolvation Temperature (°C) 400, Cone Gas Flow (L/Hr) -50, Desolvation Gas Flow (L/Hr) -750, Mass range:100 to 900 Da, DAD Wavelength range (nm): 200 to 400.

Solvent A: 0.05% Formic acid in water and Solvent B: 0.05% HCOOH in ACN: Water (90:10)

Flow rate: 1.2 ml/min, (mobile phase: 90% [0.05% HCOOH in water] and 10% [0.05% HCOOH in ACN: Water (90:10)] held for 0.75 min, then 50% [0.05% HCOOH in water] and 50% [0.05% HCOOH in ACN: Water (90:10)] in 1.0 min, further to 2% [0.05% HCOOH in water] and 98% [0.05% HCOOH in ACN: Water (90:10)] in 2.00 min, held this mobile phase composition up to 2.25 min and finally back to initial condition in 2.60 min and held this composition up to 3.00 min).

**TIME**	**Flow Rate (ml/min)**	**%A (**0.05% HCOOH in water**)**	**% B (**0.05% HCOOH in ACN: Water (90:10) **)**
**0.00**	**1.20**	**90**	**10**
**0.75**	**1.2**	**90**	**10**
**1.00**	**1.2**	**50**	**50**
**2.00**	**1.2**	**2**	**98**
**2.25**	**1.2**	**2**	**98**
**2.60**	**1.2**	**90**	**10**

**3.00**	**1.2**	**90**	**10**

Column Used: YMC Triart C18 (2.1 x 33 mm, 3 micron)

Column Temperature: 45 °C.

Method-B

Agilent 1260 Infinity II UPLC attached with Agilent SQD mass spectrometer.

Ionisation method: Electro spray, Capillary needle voltage was 4.00 kV, source temperature 350 °C. Desolvation gas flow 12 L/Min, Mass range:100 to 900 Da

DAD Wavelength range (nm): 200 to 400, Solvent A: 0.1% HCOOH in water and Solvent B: 0.1% HCOOH in CAN, Flow rate: 1.00 ml/min.

(mobile phase: 95% [0.05% HCOOH in water] and 5% [0.1% HCOOH in ACN] held for 0.50 min, then 99% [0.1% HCOOH in ACN] and 1% [0.1% HCOOH in water] in 3.0 min, held this mobile phase composition up to 4.0 min and finally back to initial condition in 4.10 min).

**TIME**	**Flow Rate (ml/min)**	**%A (**0.05% HCOOH in water**)**	**% B (**0.1% HCOOH in ACN**)**
**0.00**	**1.00**	**95**	**5**
**0.50**	**1.00**	**95**	**5**
**3.00**	**1.00**	**1**	**99**
**4.00**	**1.00**	**1**	**99**
**4.10**	**1.00**	**95**	**5**

**4.50**	**1.00**	**95**	**5**

Column Used: YMC Triart C18 column (3 µm, 33 x 2.1 mm)

Column Temperature: 40 °C.

I) <u>SAL-0010003 (VSAL-0000060)</u>

5-(2,4-difluorobenzyl)-3-isopropyl-1,6-dihydro-7H-pyrazolo[4,3-d]pyrimidin-7-one

<u>LCMS</u>

LCMS (Method A): m/z $[M+H]^+$ = 305.4 (MW calc. 304); R_t = 1.62 min.

<u>NMR</u>

<u>CR436-13381-30-VSAL-0000060</u>

^{1}H NMR (400 MHz, DMSO) δ 13.58 (s, 1H), 12.27 (s, 1H), 7.39 (q, *J* = 8.1 Hz, 1H), 7.22 (t, *J* = 9.3 Hz, 1H), 7.05 (t, *J* = 6.8 Hz, 1H), 3.97 (s, 2H), 3.16 – 3.10 (m, 1H), 1.25 (d, *J* = 7.1 Hz, 6H).

II) <u>SAL-0010042 (VSAL-0000066)</u>

5-(2-fluorobenzyl)-3-isopropyl-1,6-dihydro-7H-pyrazolo[4,3-d]pyrimidin-7-one

<u>LCMS</u>

LCMS (Method B): m/z $[M+H]^+$ = 287.1 (MW calc. 286); R_t = 2.29 min.

<u>NMR</u>

<u>CR436-13569-23-VSAL66</u>

^{1}H NMR (400 MHz, DMSO) δ 13.69 – 13.53 (m, 1H), 12.31 – 12.07 (m, 1H), 7.34 – 7.28 (m, 2H), 7.22 – 7.11 (m, 2H), 3.98 (s, 2H), 3.24 – 3.07 (m, 1H), 1.26 (d, *J* = 6.7 Hz, 6H).

III) **SAL-0010007 (VSAL-0000021)**

5-(6-fluorochroman-4-yl)-3-isopropyl-1,6-dihydro-7H-pyrazolo[4,3-d]pyrimidin-7-one

LCMS

LCMS (Method A): m/z $[M+H]^+$ = 329.3 (MW calc. 328.13); R_t = 1.64 min.

NMR

CR436-13381-90-VSAL-0000021

^{1}H NMR (400 MHz, DMSO) δ 13.62 – 13.56 (m, 1H), 12.20 – 12.09 (m, 1H), 6.99 – 6.94 (m, 1H), 6.89 – 6.78 (m, 3H), 4.46 – 4.40 (m, 1H), 4.19 – 4.15 (m, 2H), 3.17 – 3.13 (m, 1H), 2.30 – 2.25 (m, 1H), 2.21 – 2.17 (m, 1H), 1.26 (d, *J* = 6.9 Hz, 6H).

IV) **SAL-0010031 (VSAL-0000093)**

5-(3-ethyl-2-(2-methoxyethyl)-7-oxo-6,7-dihydro-2H-pyrazolo[4,3-d]pyrimidin-5-yl)-6-isobutoxy-N-methylnicotinamide

LCMS

LCMS (Method A): m/z $[M+H]^+$ = 429.5 (MW calc. 428.22); R_t = 1.52 min.

NMR

CR436-13478-49-VSAL93

^{1}H NMR (400 MHz, DMSO) δ 11.70 (s, 1H), 8.73 (s, 1H), 8.57 (s, 1H), 8.40 (s, 1H), 4.50 – 4.44 (m, 2H), 4.18 (d, *J* = 6.5 Hz, 2H), 3.80 (t, *J* = 5.2 Hz, 2H), 3.23 (s, 3H), 2.97 (q, *J* = 7.6 Hz, 2H), 2.80 (d, *J* = 4.3 Hz, 3H), 2.06 – 1.98 (m, 1H), 1.28 (t, *J* = 7.4 Hz, 3H), 0.96 (d, *J* = 6.0 Hz, 6H).

V) **SAL-0010034 (VSAL-0000100)**

5-(6'-butoxy-[2,3'-bipyridin]-5'-yl)-3-ethyl-2-(2-methoxyethyl)-2,6-dihydro-7H-pyrazolo[4,3-d]pyrimidin-7-one

LCMS

LCMS (Method B): m/z $[M+H]^+$ = 449.4 (MW calc. 448.22); R_t = 2.22 min.

NMR

CR436-13478-60-VSAL100

^{1}H NMR (400 MHz, MeOD) δ 8.90 – 8.83 (m, 2H), 8.67 – 8.61 (m, 1H), 7.97 – 7.88 (m, 2H), 7.38 (q, *J* = 4.7 Hz, 1H), 4.55 – 4.49 (m, 4H), 3.88 (t, *J* = 5.1 Hz, 2H), 3.09 (q, *J* = 7.6 Hz, 2H), 1.91 – 1.79 (m, 2H), 1.60 – 1.46 (m, 2H), 1.37 (t, *J* = 7.6 Hz, 3H), 0.99 (t, *J* = 7.4 Hz, 3H).

VI) **SAL-0010028 (VSAL-0000101)**

3-ethyl-5-(2-isobutoxy-5-phenylpyridin-3-yl)-2-(2-methoxyethyl)-2,6-dihydro-7H-pyrazolo[4,3-d]pyrimidin-7-one

LCMS

LCMS (Method A): m/z $[M+H]^+$ = 448.4 (MW calc. 447.23); R_t = 1.87 min.

NMR

CR436-13478-34-VSAL101

^{1}H NMR (400 MHz, DMSO) δ 11.72 (s, 1H), 8.60 (s, 1H), 8.29 (s, 1H), 7.73 (d, *J* = 7.6 Hz, 2H), 7.50 (t, *J* = 7.5 Hz, 2H), 7.40 (t, *J* = 7.3 Hz, 1H), 4.48 (t, *J* = 5.2 Hz, 2H), 4.18 (d, *J* = 6.5 Hz, 2H), 3.80 (t, *J* = 5.3 Hz, 2H), 3.23 (s, 3H), 2.97 (q, *J* = 7.4 Hz, 2H), 2.10 – 1.99 (m, 1H), 1.27 (t, *J* = 7.5 Hz, 3H), 0.97 (d, *J* = 6.7 Hz, 6H).

VII) **SAL-0010039 (VSAL-0000145)**

5-(5-bromo-2-butoxypyridin-3-yl)-3-ethyl-2-(2-methoxyethyl)-2,6-dihydro-7H-pyrazolo[4,3-d]pyrimidin-7-one

LCMS

LCMS (Method B): m/z $[M+H]^+$ = 450.1 (MW calc. 449.11); R_t = 2.35 min.

NMR

CR436-13478-76-VSAL145

^{1}H NMR (400 MHz, DMSO) δ 11.69 (s, 1H), 8.43 (d, *J* = 2.5 Hz, 1H), 8.19 (d, *J* = 2.5 Hz, 1H), 4.48 (t, *J* = 5.2 Hz, 2H), 4.32 (t, *J* = 6.5 Hz, 2H), 3.79 (t, *J* = 5.1 Hz, 2H), 3.22 (s, 3H), 2.96 (q, *J* = 7.5 Hz, 2H), 1.74 – 1.62 (m, 2H), 1.42 – 1.35 (m, 2H), 1.27 (t, *J* = 7.5 Hz, 3H), 0.88 (t, *J* = 7.4 Hz, 3H).

VIII) SAL-0010203 (VSAL-0000146)

5-(5-bromo-2-butoxypyridin-3-yl)-3-isopropyl-1,6-dihydro-7H-pyrazolo[4,3-d]pyrimidin-7-one

LCMS

LCMS (Method B): m/z $[M+H]^+$ = 406.1 (MW calc. 405.08); R_t = 2.35 min.

NMR

CR436-13478-83-VSAL146-NEW

^{1}H NMR (400 MHz, DMSO) δ 13.79 (s, 1H), 12.14 (s, 1H), 8.44 (s, 1H), 8.20 (d, *J* = 2.7 Hz, 1H), 4.32 (t, *J* = 6.5 Hz, 2H), 1.67 (t, *J* = 7.3 Hz, 2H), 1.36 (d, *J* = 6.8 Hz, 8H), 0.88 (t, *J* = 7.4 Hz, 3H).

IX) SAL-0010243 (VSAL-0000204)

4-isobutoxy-3-(3-isopropyl-7-oxo-6,7-dihydro-1H-pyrazolo[4,3-d]pyrimidin-5-yl)-N,N-dimethylbenzenesulfonamide

LCMS

LCMS (Method A): m/z $[M+H]^+$ = 434.3 (MW calc. 433); R_t = 1.71 min.

NMR

CR436-13569-84-VSAL204-MEOD

^{1}H NMR (400 MHz, MeOD) δ 8.18 (s, 1H), 7.91 (d, *J* = 8.8 Hz, 1H), 7.37 (d, *J* = 8.9 Hz, 1H), 4.01 (d, *J* = 6.4 Hz, 2H), 3.46 – 3.41 (m, 1H), 2.71 (s, 6H), 2.17 – 2.08 (m, 1H), 1.48 – 1.38 (m, 6H), 1.03 (d, *J* = 6.8 Hz, 6H).

X) SAL-0010255 (VSAL-0000224)

5-(3-ethyl-2-(2-methoxyethyl)-7-oxo-6,7-dihydro-2H-pyrazolo[4,3-d]pyrimidin-5-yl)-6-isobutoxy-N-isopropylnicotinamide

LCMS

LCMS (Method B): m/z $[M+H]^+$ = 457.2 (MW calc. 456); R_t = 2.55 min.

NMR

CR436-13774-24-VSAL224

^{1}H NMR (400 MHz, DMSO) δ 11.74 (s, 1H), 8.74 (d, *J* = 2.5 Hz, 1H), 8.41 (d, *J* = 2.5 Hz, 1H), 8.34 (d, *J* = 7.7 Hz, 1H), 4.48 (t, *J* = 5.2 Hz, 2H), 4.17 (d, *J* = 6.5 Hz, 2H), 4.15 – 4.06 (m, 2H), 3.80 (t, *J* = 5.1 Hz, 2H), 3.23 (s, 3H), 2.97 (q, *J* = 7.6 Hz, 2H), 2.04 – 1.97 (m, 1H), 1.27 (t, *J* = 7.5 Hz, 3H), 1.18 (d, *J* = 6.5 Hz, 6H), 0.95 (d, *J* = 6.7 Hz, 6H).

XI) SAL-0010283 (VSAL-0000266)

3-(3-cyclobutyl-7-oxo-6,7-dihydro-1H-pyrazolo[4,3-d]pyrimidin-5-yl)-4-isobutoxy-N,N-dimethylbenzenesulfonamide

LCMS

LCMS (Method B): m/z $[M+H]^+$ = 446.1 (MW calc. 445); R_t = 2.63 min.

NMR

CR436-13897-47-VSAL266-NEW

^{1}H NMR (400 MHz, $CDCl_3$) δ 11.18 (s, 1H), 10.99 (s, 1H), 8.90 (d, *J* = 2.5 Hz, 1H), 7.88 (dd, *J* = 8.6, 2.5 Hz, 1H), 7.18 (d, *J* = 8.7 Hz, 1H), 4.08 (d, *J* = 6.4 Hz, 2H), 2.77 (s, 6H), 2.66 – 2.56 (m, 2H), 2.51 – 2.44 (m, 2H), 2.37 – 2.31 (m, 1H), 2.21 – 2.09 (m, 1H), 2.08 – 1.99 (m, 1H), 1.16 (d, *J* = 6.7 Hz, 6H).

XII) SAL-0010284 (VSAL-0000277)

3-(3-(tert-butyl)-7-oxo-6,7-dihydro-1H-pyrazolo[4,3-d]pyrimidin-5-yl)-4-isobutoxy-N,N-dimethylbenzenesulfonamide

LCMS

LCMS (Method B): m/z $[M+H]^+$ = 448.2 (MW calc. 447); R_t = 2.74 min.

NMR

CR436-13897-52-VSAL277

^{1}H NMR (400 MHz, $CDCl_3$) δ 11.03 – 10.98 (m, 1H), 10.89 – 10.79 (m, 1H), 8.88 (d, *J* = 2.5 Hz, 1H), 7.89 (d, *J* = 8.3 Hz, 1H), 7.18 (d, *J* = 8.5 Hz, 1H), 4.08 (d, *J* = 6.4 Hz, 2H), 2.77 (s, 6H), 2.39 – 2.31 (m, 1H), 1.56 (d, *J* = 3.7 Hz, 9H), 1.17 (d, *J* = 6.6 Hz, 6H).

XIII) **SAL-0010301 (VSAL-0000278)**

6-isobutoxy-5-(3-isopropyl-7-oxo-6,7-dihydro-1H-pyrazolo[4,3-d]pyrimidin-5-yl)-N-methylnicotinamide

LCMS

LCMS (Method A): m/z $[M+H]^+$ = 385.4 (MW calc. 384); R_t = 1.51 min.

NMR

CR436-13993-55-VSAL278

^{1}H NMR (400 MHz, $CDCl_3$) δ 11.11 (s, 1H), 10.52 – 10.44 (m, 1H), 9.12 (d, *J* = 2.5 Hz, 1H), 8.72 (d, *J* = 2.4 Hz,1H), 6.15 (s, 1H), 4.45 (d, *J* = 6.6 Hz, 2H), 3.52 – 3.48 (m, 1H), 3.08 (d, *J* = 4.8 Hz, 3H), 2.31 – 2.27 (m, 1H), 1.48 (d, *J* = 7.0 Hz, 6H), 1.13 (d, *J* = 6.7 Hz, 6H).

XIV) **SAL-0010333 (VSAL-0000321)**

N-ethyl-6-isobutoxy-5-(3-isopropyl-7-oxo-6,7-dihydro-1H-pyrazolo[4,3-d]pyrimidin-5-yl)nicotinamide

LCMS

LCMS (Method A): m/z $[M+H]^+$ = 399.3 (MW calc. 398); R_t = 1.64 min.

NMR

CR436-14266-8-VSAL321-NEW

^{1}H NMR (400 MHz, MeOD) δ 8.75 (d, *J* = 2.5 Hz, 1H), 8.66 (d, *J* = 2.5 Hz, 1H), 4.32 (d, *J* = 6.5 Hz, 2H), 3.43 (q, *J* = 7.2 Hz, 2H), 2.20 – 2.09 (m, 1H), 1.45 (d, *J* = 6.9 Hz, 6H), 1.31 – 1.27 (m, 1H), 1.23 (t, *J* = 7.2 Hz, 3H), 1.04 (d, *J* = 6.7 Hz, 6H).

XV) SAL-0010328 (VSAL-0000405)

5-(2-fluoro-4-(trifluoromethoxy)benzyl)-3-isopropyl-1,6-dihydro-7H-pyrazolo[4,3-d]pyrimidin-7-one

LCMS

LCMS (Method A): m/z $[M+H]^+$ = 371.1 (MW calc. 370); R_t = 1.64 min.

NMR

CR436-14112-69-VSAL405

^{1}H NMR (400 MHz, $CDCl_3$) δ 8.79 – 8.75 (m, 1H), 7.39 (t, *J* = 8.5 Hz, 1H), 7.04 (d, *J* = 9.1 Hz, 2H), 4.06 (s, 2H), 3.40 – 3.36 (m, 1H), 1.42 (d, *J* = 7.0 Hz, 6H).

XVI) SAL-0010355 (VSAL-0000496)

5-(5-(3-(dimethylamino)azetidine-1-carbonyl)-2-isobutoxyphenyl)-3-isopropyl-1,6-dihydro-7H-pyrazolo[4,3-d]pyrimidin-7-one

LCMS

LCMS (Method A): m/z $[M+H]^+$ = 453.22 (MW calc. 452.25); R_t = 1.67 min.

NMR

CR436-14321-64-VSAL496

^{1}H NMR (400 MHz, DMSO) δ 13.71 (s, 1H), 11.93 (s, 1H), 7.95 (d, *J* = 2.3 Hz, 1H), 7.77 (dd, *J* = 8.7, 2.3 Hz, 1H), 7.20 (d, *J* = 8.7 Hz, 1H), 4.36 – 4.32 (m, 1H), 4.15 – 4.10 (m, 1H), 4.08 – 4.04 (m, 1H), 3.92 (d, *J* = 6.3 Hz, 2H), 3.85 – 3.81 (m, 1H), 3.10 – 3.05 (m, 1H), 2.09 (s, 6H), 2.06 – 1.98 (m, 1H), 1.37 (d, *J* = 7.0 Hz, 6H), 0.95 (d, *J* = 6.7 Hz, 6H).

XVII) SAL-0010419 (VSAL-0000589)

3-ethyl-5-(6-isobutoxy-[3,3'-bipyridin]-5-yl)-2-(2-methoxyethyl)-2,6-dihydro-7H-pyrazolo[4,3-d]pyrimidin-7-one

LCMS

LCMS (Method A): m/z $[M+H]^+$ = 449.26 (MW calc. 448.22); R_t = 1.93 min.

NMR

CR436-16294-10-VSAL589

^{1}H NMR (400 MHz, DMSO) δ 11.76 (s, 1H), 8.96 (dd, *J* = 2.4, 0.9 Hz, 1H), 8.68 (d, *J* = 2.5 Hz, 1H), 8.60 (dd, *J* = 4.8, 1.6 Hz, 1H), 8.37 (d, *J* = 2.5 Hz, 1H), 8.19 – 8.12 (m, 1H), 7.55 – 7.48 (m, 1H), 4.48 (t, *J* = 5.2 Hz, 2H), 4.19 (d, *J* = 6.5 Hz, 2H), 3.80 (t, *J* = 5.2 Hz, 2H), 3.23 (s, 3H), 2.97 (q, *J* = 7.5 Hz, 2H), 2.12 – 1.97 (m, 1H), 1.27 (t, *J* = 7.5 Hz, 3H), 0.97 (d, *J* = 6.7 Hz, 6H).

XVIII) SAL-0010145 (VSAL-0000849)

5-(2-ethoxy-5-((4-methylpiperazin-1-yl)sulfonyl)phenyl)-3-propyl-1,6-dihydro-7H-pyrazolo[4,3-d]pyrimidin-7-one

LCMS

LCMS (Method A): m/z $[M+H]^+$ = 461.42 (MW calc. 460.19); R_t = 2.15 min.

NMR

CR656F-21996-10-VSAL849

^{1}H NMR (401 MHz, DMSO) δ 13.69 – 13.30 (m, 1H), 11.70 – 11.40 (m, 1H), 8.03 (d, *J* = 2.5 Hz, 1H), 7.83 (dd, *J* = 8.9, 2.5 Hz, 1H), 7.37 (d, *J* = 8.8 Hz, 1H), 4.29 (q, *J* = 7.0 Hz, 2H), 3.01 (t, *J* = 4.8 Hz, 4H), 2.86 (t, *J* = 7.4 Hz, 2H), 2.39 (t, *J* = 4.9 Hz, 4H), 2.18 (s, 3H), 1.84 – 1.76 (m, 2H), 1.39 (t, *J* = 6.9 Hz, 3H), 0.97 (t, *J* = 7.4 Hz, 3H).
